# Prognostic association supports indexing size measures in echocardiography by body surface area

**DOI:** 10.1038/s41598-023-46183-z

**Published:** 2023-11-08

**Authors:** Angus S. Y. Fung, Dhnanjay Soundappan, Daniel E. Loewenstein, David Playford, Geoffrey Strange, Rebecca Kozor, James Otton, Martin Ugander

**Affiliations:** 1 Kolling Institute, Royal North Shore Hospital, and University of Sydney, Kolling Building, Level 12, St Leonards, Sydney, NSW 2065 Australia; 2 St Vincent’s Clinical School, University of New South Wales, Sydney, Australia; 3 Department of Clinical Physiology, Karolinska University Hospital, and Karolinska Institutet, Stockholm, Sweden; 4 School of Medicine, University of Notre Dame, Fremantle, Australia; 5 Faculty of Medicine and Health, University of Sydney, Sydney, Australia; 6 Department of Cardiology, Liverpool Hospital, University of New South Wales, Liverpool, Australia

**Keywords:** Physiology, Cardiology, Risk factors

## Abstract

Body surface area (BSA) is the most commonly used metric for body size indexation of echocardiographic measures, but its use in patients who are underweight or obese is questioned (body mass index (BMI) < 18.5 kg/m^2^ or ≥ 30 kg/m^2^, respectively). We aim to use survival analysis to identify an optimal body size indexation metric for echocardiographic measures that would be a better predictor of survival than BSA regardless of BMI. Adult patients with no prior valve replacement were selected from the National Echocardiography Database Australia. Survival analysis was performed for echocardiographic measures both unindexed and indexed to different body size metrics, with 5-year cardiovascular mortality as the primary endpoint. Indexation of echocardiographic measures (left ventricular end-diastolic diameter [n = 230,109] and mass [n = 224,244], left atrial volume [n = 150,540], aortic sinus diameter [n = 90,805], right atrial area [n = 59,516]) by BSA had better prognostic performance vs unindexed measures (underweight: C-statistic 0.655 vs 0.647; normal weight/overweight: average C-statistic 0.666 vs 0.625; obese: C-statistic 0.627 vs 0.613). Indexation by other body size metrics (lean body mass, height, and/or weight raised to different powers) did not improve prognostic performance versus BSA by a clinically relevant magnitude (average C-statistic increase ≤ 0.02), with smaller differences in other BMI subgroups. Indexing measures of cardiac and aortic size by BSA improves prognostic performance regardless of BMI, and no other body size metric has a clinically meaningful better performance.

## Introduction

Quantification of the dimensions of the heart and great vessels using echocardiography has both diagnostic and prognostic value for the prediction of morbidity and mortality, which also may help guide treatment in patients^1–8^. Historically, the recommended method for body size indexation of cardiac volumes has been body surface area (BSA)^9^. More recently, recommendations for the indexation of left ventricular mass by an allometric measure of height (raised to the 2.7) have been proposed^10,11^. However, there is heterogeneity in the literature as to the best indexation method, and whether or not indexing of cardiac measures improves their predictive value for cardiovascular events^6,12–14^. In underweight and overweight patients, correction for BSA can overestimate or underestimate the prevalence of left ventricular hypertrophy, and inaccurately normalise or exaggerate indices of cardiac size^12^.

Studies on indexation for prognostic performance have been limited in patient sample size and range of cardiac measures indexed^5,15–18^. Using the large-scale data available in the National Echocardiography Database of Australia (NEDA), the aim of the study was to derive one or more formulae based on height and weight to provide a method of body size indexation of cardiac and aortic measures that will be a better predictor of cardiovascular mortality than current methods based on BSA.

## Methods

### Study design

NEDA is a large observational registry that includes routinely recorded echocardiographic data across 30 centres in Australia. Individual data linkage is used to incorporate health outcomes such as cardiovascular and all-cause mortality. The study cohort consists of patients over the age of 18 who have typically been referred clinically for imaging evaluation of known or suspected cardiovascular disease. Subjects were included following either a retrospective waiver of individual informed consent, or the absence of a prospective decision to opt out of enrolling in the study. The study complies with the Declaration of Helsinki, and the study and its design, including the retrospective waiver of consent, was approved by the lead ethics committee, at the Royal Prince Alfred Hospital (2019/ETH06989). NEDA is registered with the Australian New Zealand Clinical Trials Registry (ACTRN12617001387314). Ethical approval has been obtained from the Human Research Ethics Committees at the respective recruiting sites.

### Study cohort

**Figure 1:**
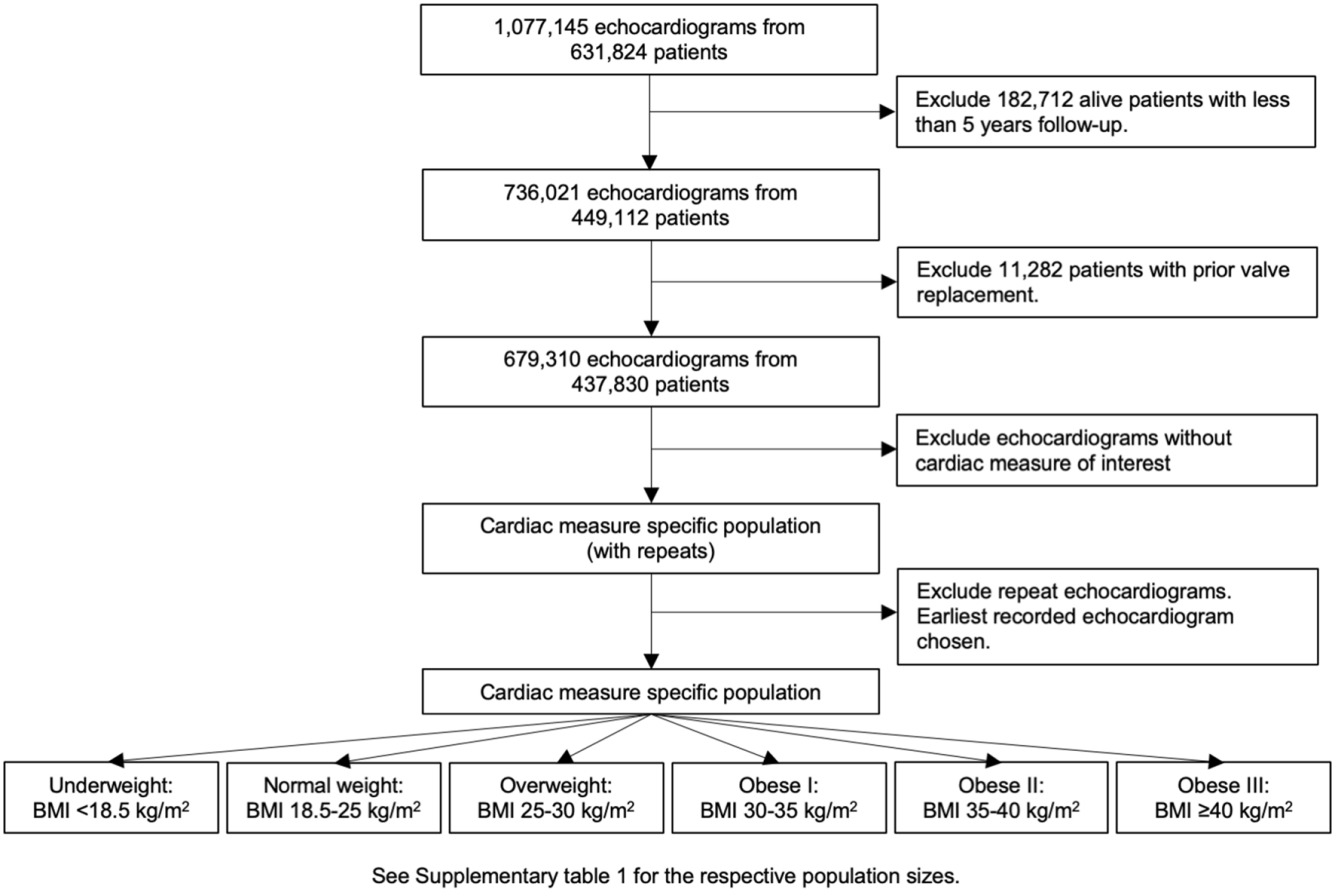
Flowchart describing patient inclusion. Exact numbers for the respective cardiac measure specific populations are given in Supplementary Table 1.

Echocardiographic data and basic patient characteristics were collected from participating centres from 1 January 2000 to 21 May 2019, and were transferred into a central database via an automated data extraction process. Echocardiographic measurements were made in accordance with guidelines from the American Society of Echocardiography^9^. All data was cleaned through the removal of duplicate, inconsistent, and/or impossible measurements, and transformed into a standardized format. Individuals contributing to NEDA were assigned a unique identifier linked to their echocardiograms and their anonymity protected by stringent security protocols. As shown in Fig. 1, 631,824 patients were present in the database. Of these, 182,712 (17%) patients were excluded for having less than 5 years of follow-up time and a further 11,282 (1%) were excluded for prior valve replacement. Echocardiograms with time from echocardiography to census or death, cause of mortality (cardiovascular and all-cause), height, weight, and the cardiac measure of interest were selected. Different populations were individually filtered for each measure of interest and analysed to maximise the number of patients for analysis. For patients with multiple echocardiograms, the earliest recorded echocardiogram was selected. Patients were grouped according to body mass index (BMI) subgroups outlined by the National Institutes of Health and World Health Organisation (underweight: BMI < 18.5 kg/m^2^, normal weight: BMI 18.5–25 kg/m^2^, overweight: BMI 25–30 kg/m^2^, obesity class I: BMI 30–35 kg/m^2^, obesity class II: BMI 35–40 kg/m^2^, or obesity class III: BMI ≥ 40 kg/m^2^)^19^. Patients were classified as having normal echocardiographic measures, or echocardiographic measures with mild or moderate abnormalities, according to values outlined by the American Society of Echocardiography and the European Association of Cardiovascular Imaging (Normal: left ventricular ejection fraction ≥ 55%; left ventricular end-diastolic diameter between 37–56 and 35–51 mm for males and females, respectively; left atrial volume index < 34 ml/m^2^; aortic and pulmonary valve peak velocity ≤ 2 m/s; tricuspid valve peak velocity ≤ 2.8 m/s; mitral E/eʹ < 8; normal or mild mitral, tricuspid, or aortic valve regurgitation; no aortic stenosis; Mild: left ventricular ejection fraction 50–55%; left ventricular end-diastolic diameter between 56–61 and 51–55 mm for males and females, respectively; left atrial volume index 34–38 ml/m^2^; aortic and pulmonary valve peak velocity 2–3 m/s; mitral E/eʹ 8–15; mild aortic stenosis)^20–22^.

### Endpoints

The primary endpoint of interest was cardiovascular mortality. Mortality data was obtained by linkage with the National Death Index^23^. A detailed probability matching process involving patient identifiers obtained at echocardiographic recording was used to link the survival status of individuals up to the study census date of 21 May 2019. Listed causes of death were described using ICD-10 coding, which allowed for cardiovascular death to be defined (range 100–199 ICD-10AM chapter codes)^24^.

### Statistical analysis

NEDA data analyses and reports were generated in agreement with STROBE guidelines^25^. All data used in analyses were provided and no missing data was imputed. Standard procedures for describing grouped data, such as median [interquartile range (IQR)], and proportions according to patient characteristics were applied.

Cox-proportional hazard models with proportional hazards confirmed by visual inspection and numerical analysis of Schoenfeld residuals were used to derive C-statistics and hazard-ratios (with 95% confidence intervals) for the risk of cardiovascular and all-cause mortality for the entirety of the study follow-up and a 5-year follow-up duration. The change in the Akaike information criterion (ΔAIC) was used to interpret the statistical robustness of the body size indexation metrics. Due to the magnification of ΔAIC by large sample sizes, the C-statistic was chosen to dictate the magnitude of difference between metrics and clinical relevance in a sample size independent fashion. Kaplan–Meier curves were constructed to visually inspect differences between indexation measures. An iterative function was coded to derive 50,000 combinations of body size metrics using different height and weight exponents according to the formula where a given body size metric = height^x^⋅weight^y^. Random combinations of x and y were used as the metric for indexation for the respective echocardiographic measures for subsequent Cox-regression analysis. Cox-regression was performed using the echocardiographic measure indexed by the derived body size metric with five-year cardiovascular mortality as the endpoint to obtain the C-statistic. The C-statistic was color-coded in a scatterplot to present differences in the prognostic strength of different body size metrics including BSA as calculated according to Mosteller^26^ and Du Bois^27^, and BSA raised to various powers, and height and/or weight raised to various powers. Lean body mass formulas by Hume^28^, Boer^29^, and James^30^ were also analysed and included in Table 1. All statistical analyses were performed using R 4.0.4 (R Core Team, R Foundation for Statistical Computing, Vienna, Austria)^31^. Significance was accepted at the level of p < 0.05 (two-sided).

**Table 1:** C-statistics for 5-year cardiovascular mortality for representative anatomical measures, both unindexed and indexed by different body size metrics, and their average.

	LV mass			LA volume			RA area			Aortic sinus diameter			RV diameter			LV end-diastolic diameter			Average		
	BMI < 18.5 kg/m^2^	BMI 18.5–30 kg/m^2^	BMI ≥ 30 kg/m^2^	BMI < 18.5 kg/m^2^	BMI 18.5–30 kg/m^2^	BMI ≥ 30 kg/m^2^	BMI < 18.5 kg/m^2^	BMI 18.5–30 kg/m^2^	BMI ≥ 30 kg/m^2^	BMI < 18.5 kg/m^2^	BMI 18.5–30 kg/m^2^	BMI ≥ 30 kg/m^2^	BMI < 18.5 kg/m^2^	BMI 18.5–30 kg/m^2^	BMI ≥ 30 kg/m^2^	BMI < 18.5 kg/m^2^	BMI 18.5–30 kg/m^2^	BMI ≥ 30 kg/m^2^	BMI < 18.5 kg/m^2^	BMI 18.5–30 kg/m^2^	BMI ≥ 30 kg/m^2^
Weight	0.697	0.723	0.664	0.725	0.731	0.670	0.704	0.668	0.591	0.640	0.645	0.562	0.599	0.652	0.656	0.545	0.611	0.583	0.652	0.672	0.621
Weight^1.5^	0.686	0.728	0.660	0.726	0.733	0.667	0.708	0.671	0.589	0.629	0.634	0.553	0.424	0.644	0.643	0.549	0.609	0.573	0.620	0.670	0.614
Height⋅weight	0.701	0.728	0.670	0.724	0.733	0.672	0.705	0.672	0.593	0.627	0.637	0.564	0.594	0.650	0.652	0.552	0.611	0.582	0.651	0.672	0.622
BSA[M]^1.5^	0.707	0.722	0.670	0.724	0.731	0.673	0.702	0.669	0.593	0.635	0.645	0.571	0.609	0.654	0.659	0.550	0.611	0.590	0.655	0.672	0.626
BSA[DB]^1.5^	0.711	0.721	0.671	0.723	0.730	0.674	0.701	0.669	0.594	0.633	0.644	0.574	0.611	0.653	0.660	0.554	0.610	0.592	0.655	0.671	0.628
BSA[M]	0.709	0.710	0.665	0.722	0.726	0.672	0.695	0.661	0.591	0.642	0.646	0.576	0.613	0.650	0.666	0.547	0.603	0.593	0.655	0.666	0.627
BSA[DB]	0.712	0.709	0.666	0.721	0.725	0.673	0.695	0.661	0.592	0.641	0.645	0.579	0.617	0.650	0.668	0.551	0.602	0.596	0.656	0.665	0.629
LBM[H]	0.719	0.715	0.669	0.719	0.726	0.672	0.694	0.663	0.588	0.624	0.628	0.564	0.623	0.644	0.655	0.560	0.597	0.582	0.657	0.662	0.622
LBM[B]	0.719	0.712	0.662	0.719	0.723	0.667	0.695	0.659	0.579	0.626	0.617	0.543	0.621	0.636	0.636	0.561	0.588	0.562	0.657	0.656	0.608
LBM[J]	0.703	0.714	0.659	0.719	0.724	0.666	0.697	0.660	0.577	0.626	0.619	0.546	0.606	0.637	0.635	0.539	0.588	0.562	0.648	0.657	0.607
BMI	0.678	0.693	0.637	0.719	0.719	0.657	0.687	0.647	0.579	0.624	0.615	0.533	0.566	0.632	0.646	0.489	0.581	0.558	0.627	0.648	0.602
Height^2.7^	0.726	0.706	0.672	0.718	0.723	0.677	0.692	0.662	0.594	0.620	0.618	0.580	0.657	0.642	0.662	0.578	0.595	0.596	0.665	0.658	0.630
Height^2.13^	0.724	0.702	0.669	0.719	0.721	0.676	0.690	0.659	0.593	0.628	0.621	0.584	0.666	0.642	0.666	0.574	0.593	0.600	0.667	0.656	0.631
Height^2^	0.724	0.701	0.668	0.718	0.721	0.675	0.690	0.658	0.593	0.630	0.621	0.585	0.668	0.642	0.667	0.573	0.592	0.600	0.667	0.656	0.631
Height^1.5^	0.720	0.695	0.663	0.718	0.719	0.673	0.687	0.653	0.591	0.635	0.618	0.585	0.668	0.640	0.669	0.567	0.586	0.599	0.666	0.652	0.630
Height	0.715	0.688	0.657	0.716	0.715	0.671	0.683	0.648	0.589	0.635	0.607	0.579	0.665	0.634	0.670	0.557	0.577	0.594	0.662	0.645	0.627
Unindexed	0.702	0.670	0.641	0.712	0.707	0.663	0.672	0.635	0.582	0.613	0.568	0.552	0.650	0.617	0.666	0.533	0.553	0.572	0.647	0.625	0.613

*RA* right atrial, *RV* right ventricular, *LA* left atrial, *LV* left ventricular, *BSA* body surface area, *[M]* Mosteller, *[DB]* Du Bois, *LBM* lean body mass, *[H]* Hume, *[B]* Boer, *[J]* James, *BMI* body mass index.

## Results

### Study cohort

Subject characteristics and size of the study cohorts for various cardiac and aortic measures are presented in Supplementary Table 1. Due to the large sample size, differences in baseline characteristics between BMI groups were statistically significant but were not of a clinically meaningful magnitude. Across all cohorts, an average of 27% of patients had echocardiographic measures in the normal range. A further 25% of patients had echocardiographic measures with at most mild abnormalities. The remaining 48% of patients had at least one echocardiographic measure indicating a moderate abnormality.

### Body size metrics and mortality

**Figure 2:**
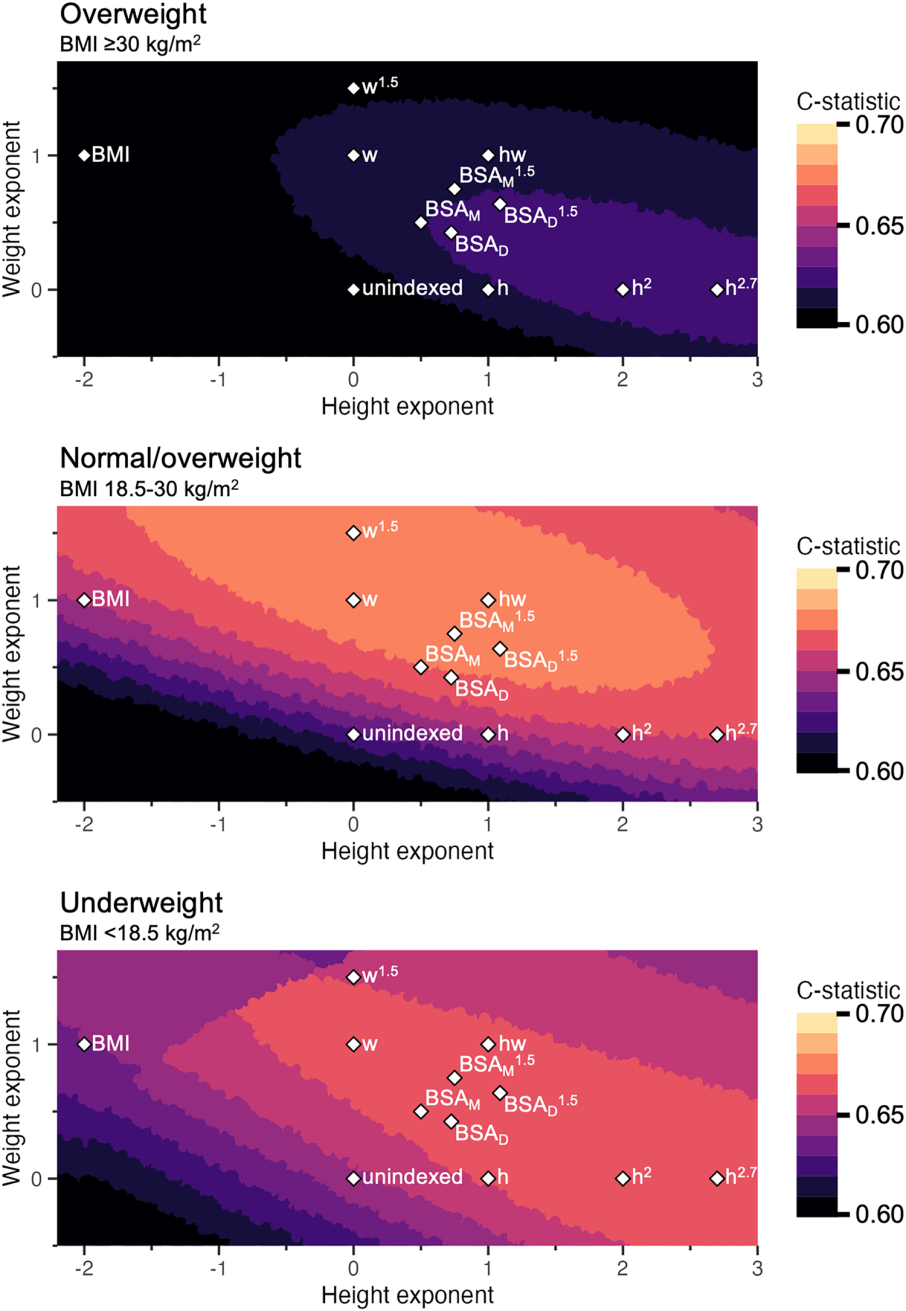
Average prognostic strength (C-statistic) for predicting 5-year cardiovascular mortality when indexing for body size for right atrial area, left atrial volume, left ventricular diameter, left ventricular mass, and aortic sinus diameter. The axes represent the height and weight exponents of a body size indexation metric of the format height^x^⋅weight^y^. The color scale shows the average C-statistic from 0.60 to 0.70, each color increment representing a 1%-point improvement. Existing body size metrics were plotted: *h* height, *w* weight, *hw* height⋅weight, *BMI* body mass index, *BSA* body surface area, *M* Mosteller, *D* DuBois. In the normal weight/overweight group, from unindexed to BSA by Mosteller there is a 4%-point improvement, but further improvement is limited to < 1%-point. Similar trends can be observed in the underweight and obese group. Echocardiographic measures with a limited number of observations were not included. See text for details.

Across the echocardiographic measures of right atrial area (n = 59,516), left atrial volume (n = 150,540), left ventricular end-diastolic diameter (n = 230,109) and mass (n = 224,244), and aortic sinus diameter (n = 90,805), indexation by BSA as calculated by Mosteller had an average C-statistic increase from 0.647 to 0.655 for the underweight cohort, an increase from 0.625 to 0.666 for the normal weight/overweight cohort, and an increase from 0.613 to 0.627 for the obese cohort compared to unindexed measures for the endpoint of 5-year cardiovascular mortality as shown in Table 1^26^. Average C-statistics are provided for composite visualisation of improvements in prognostic value across cardiac measures which followed the same trend. Indexation by other body size metrics (lean body mass, height, and/or weight raised to different powers) yielded a C-statistic increase ≤ 0.02. Further sex-disaggregated analysis did not differ upon visual inspection, and numerical differences between BSA and the best body size indexation metric were not clinically meaningful (Fig. 2, Supplementary Figs. 1–14). Smaller differences in C-statistic between indexation metrics were observed in higher BMI subgroups. Similar results (data not shown) were obtained using long-term cardiovascular mortality not limited to five years, and both long-term and five-year all-cause mortality. Furthermore, similar trends (Supplementary Tables 2–15, Supplementary Figs. 1–14) were observed across indexation of other aortic dimensions (sinotubular junction, ascending, root, arch) and cardiac chamber volumes (left atrial end-systolic diameter and area, left ventricular end-diastolic volume, right ventricular diameter). Across all measures, the 95% confidence interval of the C-statistic did not cross 0.50, thus the C-statistic remains significant for the normal weight/overweight group. Indexation by BSA by Mosteller provides an improvement in prognostic performance compared to the unindexed measure. Indexation metrics which have a better prognostic performance than BSA provide only a marginal improvement. However, indexation never negatively impacts prognostic performance. Kaplan–Meier curves (Supplementary Figs. 15–28) did not show visually appreciable differences between indexation by BSA compared to indexation by height^2.7^ across any measure.

Figure 2 shows how indexation by different body size metrics (based on the formula height^x^⋅weight^y^) performs prognostically presented as the average C-statistic for the echocardiographic measures of right atrial area (n = 59,516), left atrial volume (n = 150,540), left ventricular end-diastolic diameter (n = 230,109) and mass (n = 224,244), and aortic sinus diameter (n = 90,805). The color scale shows increasing C-statistics, where each color change represents one percentage point of C-statistic. Numerical values for selected measures are presented in Table 1. In summary, in the normal weight cohort, indexation by BSA improves prognostic performance compared to unindexed measures by four percentage points of the C-statistic. Further improvement beyond BSA is limited to < 1 percentage point improvement on average. Indexation by BSA yielded smaller improvements in prognostic performance in the underweight and obese cohort, but indexation by any other body size metric did not provide any meaningfully stronger association with survival. Further analyses in obese populations and higher BMI subgroups (BMI 30–35, 35–40 and > 40) showed that BSA performed similarly to height raised to various powers.

### Relative prognostic strength of different cardiac and aortic size measures

**Figure 3:**
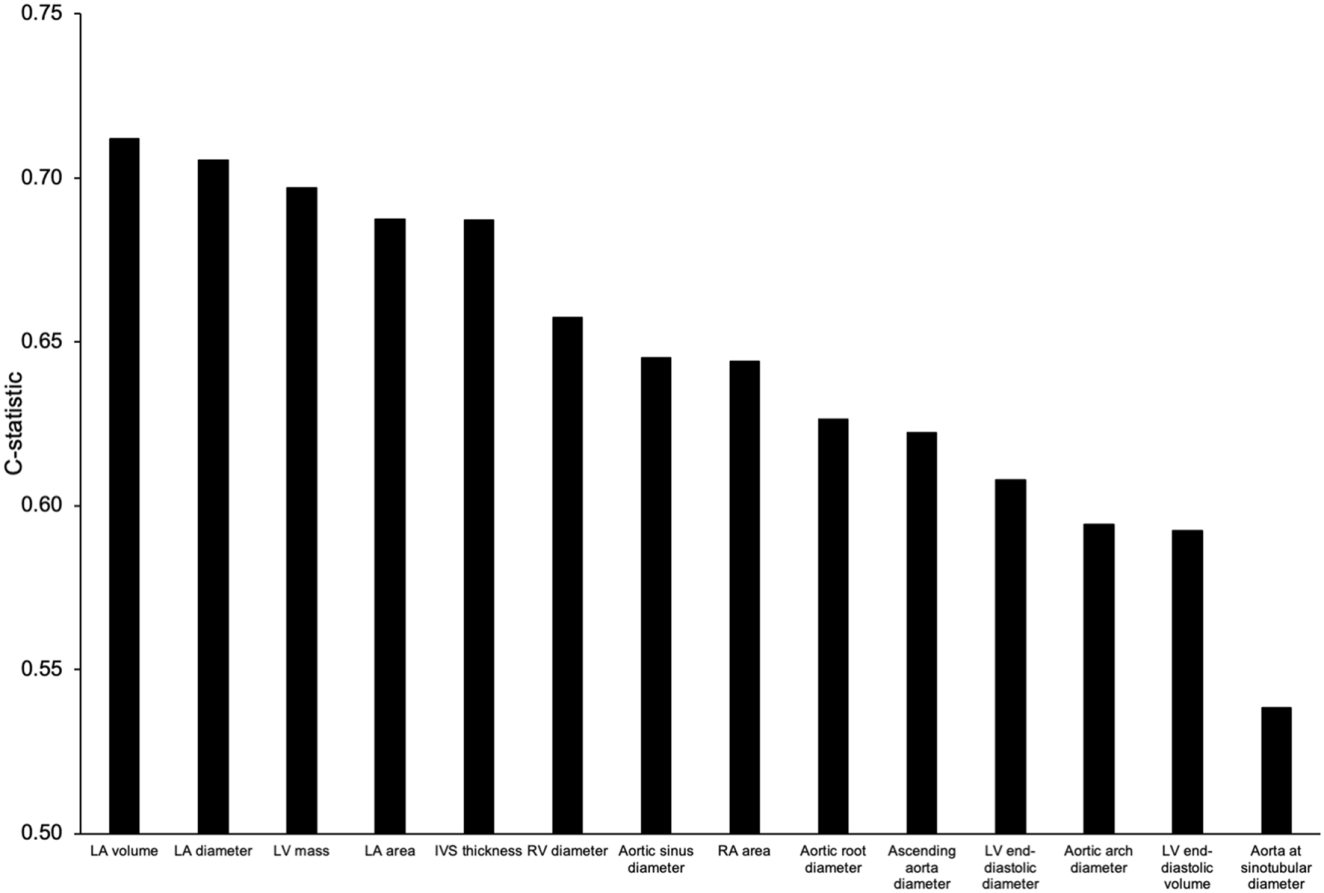
The combined prognostic strength of indexing by body surface area across cardiac measures regardless of BMI for the prediction of 5-year cardiovascular mortality. Left atrial size and left ventricular mass had the highest prognostic strength, with a C-statistic 10%-points higher than left ventricular size and most aortic dimensions. Right atrial and right ventricular sizes had an intermediate prognostic strength. Aortic sinus diameter had the strongest prognostic strength of all aortic measures, and aorta at sinotubular diameter had the weakest prognostic strength. *RA* right atrial, *RV* right ventricular, *LA* left atrial, *LV* left ventricular, *IVS* interventricular septum.

Figure 3 shows the combined prognostic strength of indexing by BSA regardless of BMI across all cardiac and aortic measures. Similar trends were observed in individual BMI groups (data not shown). Confidence intervals are not shown due to the small magnitude (< 2 percentage points for all) due to the large sample sizes. Left atrial size and left ventricular mass had the highest prognostic strength, with a C-statistic 10 percentage points higher than left ventricular size and most aortic dimensions. Right atrial and right ventricular sizes had an intermediate prognostic strength. Aortic sinus diameter had the strongest prognostic strength of all aortic measures, and aorta at sinotubular diameter had the weakest prognostic strength.

## Discussion

**Figure 4:**
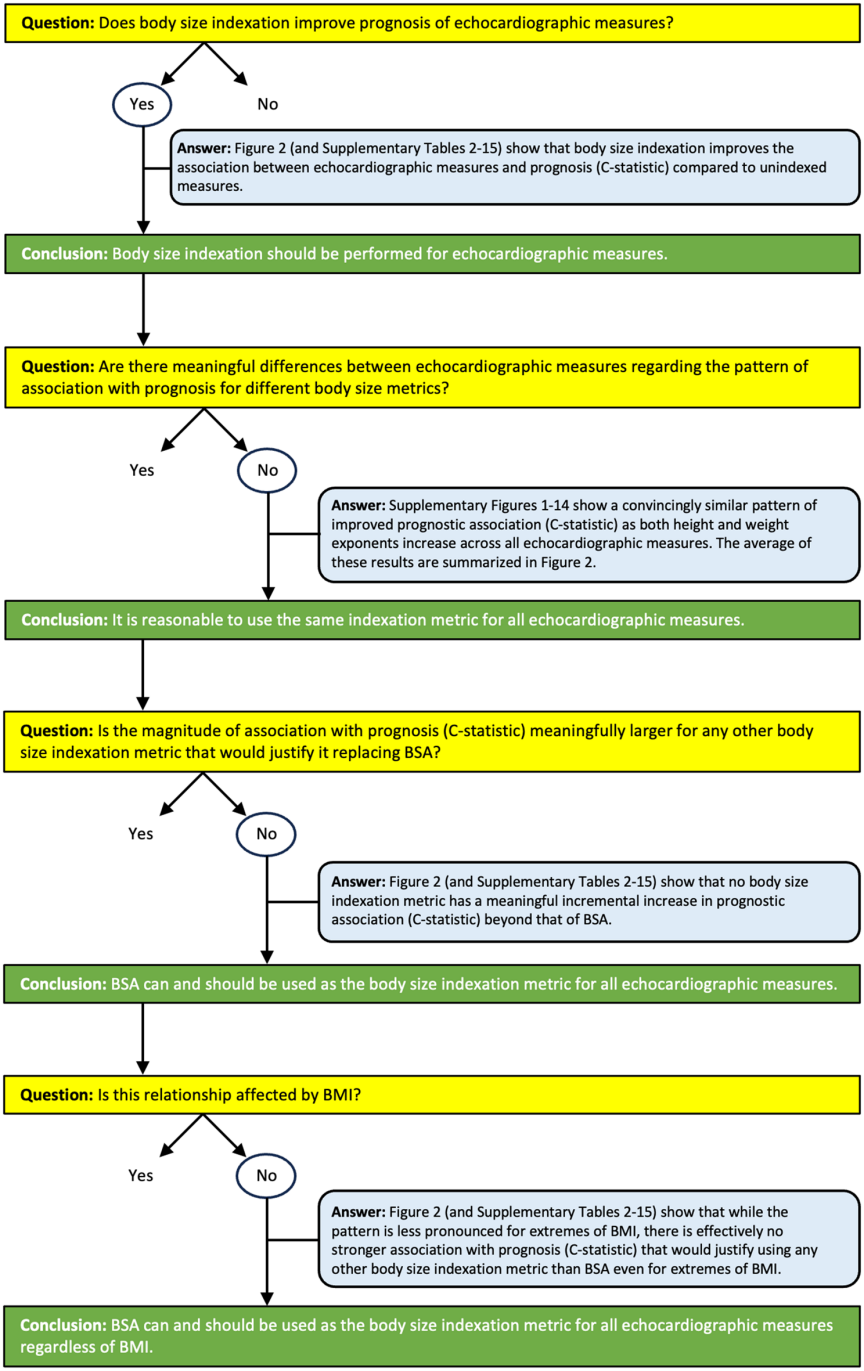
Flow-chart representing the logic, data, and reasoning for using body surface area for the indexation of all echocardiographic measures regardless of BMI.

In this study of body size indexation of cardiac and aortic sizes using real-world echocardiographic data from a large-scale nationwide cohort, indexation by BSA is shown to improve prognostic performance compared to unindexed measures regardless of BMI. Furthermore, no other body size indexation metric provided any meaningful improvement in prognostic performance beyond BSA. The current study comprehensively assessed indexation metrics with varying height and weight exponents in different forms (multiplicative, additive/subtractive, both), and in sex-specific cohorts. It was not possible to derive a body size metric with clinically meaningfully better prognostic performance than BSA. This means that using mortality as the arbiter of indexation effectiveness, no other indexation method exists that is clinically meaningfully better than BSA across all investigated cardiac measures. The logical steps and results from the current study underpinning this rationale are summarized in Fig. 4. Consequently, and in accordance with current guidelines for echocardiography^9^, cardiac measures can continue to be indexed using any formula for BSA, regardless of BMI or the echocardiographic measure of interest, and the current study supports extending this approach to aortic measures.

### Magnitude of improvement by body size indexation

In general, the magnitude of association between echocardiographic measures and survival increased after adjusting for body size. As an example, when comparing unindexed to BSA indexation for the normal/overweight group, the hazard ratio for all measures increased by on average 0.13 ± 0.11 (range 0.01–0.37). However, hazard ratios are susceptible to the reference group chosen and size of standard deviations. The C-statistic is a way of standardizing the interpretation of effect size. The C-statistic is calculated by taking all possible pairs of subjects consisting of one subject who experienced the event of interest and one who did not. The C-statistic is the proportion of such pairs in which the subject who experienced the event had a higher predicted probability of experiencing the event than the subject who did not experience the event. In light of how the C-statistic is calculated, it is reasonable to interpret the outcomes such that a change < 1 percentage point can be considered to be of negligible clinical relevance. Thus, across the studied echocardiographic measures, indexation by BSA improves the C-statistic compared to the unindexed measure. As an example, when comparing unindexed to BSA indexation for the normal/overweight group, the C-statistic for all measures increased by on average 0.037 ± 0.017 (range 0.019–0.078). Notably, Kaplan–Meier curves can be used for visualisation of trends but can be misleading in that it obscures the magnitude of the difference in prognosis, which can only be appreciated using the C-statistic.

### Comparison with indexation by height

The current study found that indexation by BSA improves prognostic performance compared to height regardless of BMI. It has been suggested that indexation by height^2.7^ improves detection of left ventricular hypertrophy and associations with cardiovascular events and mortality compared to BSA in obese populations^14,18,32^. Recently, it has been shown that indexation by height for left atrial volumes was able to maintain proportionality and avoid overcorrection for body size^33,34^. However, a consideration of prognostic value is more useful clinically than allometry. While indexation by height^2.7^ and modified versions of BSA have been shown to reclassify patients into binary groups with mortality benefit, cut-off values for these groups are arbitrary and fail to appreciate the continuous nature of such variables^35^. Further, other studies have found no improvement in indexation by height^2.7^ compared to BSA^5,36^. The findings of the current study confirm that indexation by height^2.7^ has similar prognostic value compared to indexation by BSA across any BMI group, and thus can be used interchangeability if desired.

### Prognostic strength across BMI subgroups

The current study found that the C-statistic was generally lower in higher BMI subgroups compared to normal and overweight groups (Supplementary Tables 2–15). This more blunted association with survival for body size indexation in higher BMI groups may be because BMI is an independent predictor of survival. It is likely that the risk of mortality in these groups is moreso influenced by other factors, and less attributable to their echocardiographic measures regardless of indexation metric^37^. That said, using BSA indexation for all BMI groups maintains a numerically improved association with prognosis. Taken together, BSA indexation can be used regardless of BMI.

### Age differences

The current study did not include the impact of age on cardiac or aortic size and mortality, which may be a source of uncontrolled confounding. An increase in age has long been established to be related to an increased aortic size, which would affect interpretation of the indexed measure^38,39^. The association between age and mortality is both intuitive and widely accepted^40,41^. However, a consideration of age in choice of body size indexation metric is impractical clinically, and fails to achieve the goal of indexation, namely, to account for body size. Given the strong association of age with mortality, the inclusion of age into statistical models overcasts differences between indexation metrics. A consideration of age is more appropriate for cut-off values of the indexed cardiac and aortic size measure, but not necessarily for the choice of body size indexation metric. Importantly, similar age distributions existed between the respective cohorts in the current study. Thus, while theoretically attractive, consideration of the effect of age does not contribute to addressing the purpose of the optimal choice of body size indexation method per se.

### Sex differences

The current study found that indexation of cardiac and aortic measures disaggregated by sex does not improve prognostic performance. Differences in left ventricular mass have been found between male and female patients^42,43^. A consideration of sex in the indexation of cardiac measures measured by cardiac magnetic resonance imaging has been suggested for improved prediction of incident heart failure^13^. The current study analysed sex-specific cohorts and found that body size metrics derived from sex-specific cohorts were effectively interchangeable with negligible differences in prognostic performance (Fig. 2, Supplementary Figs. 1–14). Thus, the findings of the current study suggest that the relationship between cardiac or aortic size and survival in relation to body size does not fundamentally differ between the sexes. Notably, this is still consistent with using different sex-specific cut-offs for normality for a given measure, indexed by the same choice of body size metric for both sexes.

### Allometry

Furthermore, there are concerns regarding the physiological relationships between left ventricular mass and indexation methods. While the relationships between body surface area, height, and weight are non-linear, the indexation of left ventricular mass by these variables often assumes linear relationships^44^. An argument can also be drawn from the theory of similarity, which reasons that relative geometries are best indexed to body size variables of similar dimensionality. For example, since left ventricular mass is related to cardiac dimensions raised to the third power, and BSA is related to a body dimension raised to the second power, it is logical that left ventricular mass should be proportional to BSA^3/2^ (also expressed as BSA^1.5^)^45^. Despite these theoretical considerations, the results of the current study show a continuum in the association between varying body size metrices with prognosis that does strictly require similar dimensionality.

### Prognostic strength of aortic measures

The notion that aortic measures would index similarly to body size was not immediately apparent prior to this study. Aortic measures generally had a weaker prognostic strength than measures of left atrial size and left ventricular mass, which had a C-statistic 10 percentage points higher than most aortic dimensions. Aortic sinus diameter had the strongest prognostic strength of all aortic measures, and aorta at sinotubular diameter had the weakest prognostic strength. While the improvement in association is stronger for cardiac measures than aortic measures, body size indexation by BSA still remains important for aortic measures.

### Study cohort

Across all cohorts, an average of 27% of patients had echocardiographic measures in the normal range, and a further 25% had echocardiographic measures in the mild range. In a survey in 2013 with responses from 89% of all echocardiography laboratories in Sweden, a weighted average of 34% of all echocardiographic studies showed essentially normal findings. On a per-laboratory basis, the normalcy rate (mean ± SD) was 39 ± 18%, range 11–79%, with the highest normalcy rates among privately operating outpatient laboratories and lowest normalcy rates for publicly operating hospital laboratories^46^. In light of those findings, the normalcy rate in the current study is typical in this regard, further underscoring the applicability of the large-scale real-world results of the current study to other echocardiography laboratories.

### Strengths and limitations

The limitations of applying and interpreting big data in NEDA have been acknowledged previously^24,47^. At the time of analysis, NEDA did not include important clinical details of common conditions such as coronary artery disease, ischaemic heart disease, and clinically diagnosed heart failure, which may impact mortality. Prevalent cardiovascular disease, namely myocardial infarction, can alter the natural relationship between anthropometric parameters and heart structures, and this could not be accounted for in this study^48^. NEDA does not include any information on race thus any such subanalyses could not be undertaken. That said, the current study used large-scale, real-world data with relevant clinical outcomes that inevitably have measurement variability between centres and observers. While on one hand, this is a limitation as an uncontrolled source of data heterogeneity, it is in fact a strength that reinforces the integrity of observed trends that exist despite sources of variability.

The use of data from NEDA also results in confounding by indication, wherein the inclusion of patients is biased by their need for an echocardiogram. However, this is not a limitation but rather a strength as indexation metrics are intended to be applied to such patients.

The main endpoint of consideration, cardiovascular mortality, was linked from the Australian National Death Index which has a high sensitivity and specificity (93% and 90%, respectively) validating its use as the primary endpoint^23^. Furthermore, in the current study, similar trends were also observed across all-cause mortality, further reinforcing the validity of the results.

The NEDA cohort is a large population that is representative of the diverse and multiethnic population in Australia. However, the applicability of the proposed body size indexation may be different in specific clinical settings.

The current study has definitively demonstrated that measures of cardiac and aortic size should continue to be indexed by BSA regardless of BMI. No other existing or derived body size metric (lean body mass or height and/or weight raised to various powers) is clinically meaningfully better. Left atrial size and left ventricular mass indexed to BSA provided the strongest prognostic association of all transthoracic echocardiographic size measures. The results from this study do not show compelling evidence for using different indexation metrics for different structures measurable by transthoracic echocardiography. In the setting of using just one indexation metric, there is no evidence to use anything other than BSA. Further studies on establishing the normal range for echocardiographic measures based on the association with mortality have been performed for aortic stenosis^47^, estimated pulmonary arterial pressure^49^, and diastolic dysfunction^24^. Future studies are now justified to revisit the appropriateness of normal ranges for structural echocardiographic measures indexed to body size, adjusted for age and sex, based on the association with prognosis.

### Supplementary Information

Supplementary Figures.

Supplementary Tables.

## Data Availability

The datasets used and/or analysed during the current study are available from the corresponding author upon reasonable request.
