# Supplementary material for: Prognostic association supports indexing size measures in echocardiography by body surface area
Source: Sci Rep. 2023 Nov 8;13:19390. doi: 10.1038/s41598-023-46183-z (PMC10632399; doi:10.1038/s41598-023-46183-z)
Supplement: Supplementary file 1 — Supplementary Figures. [file 41598_2023_46183_MOESM1_ESM.pdf]

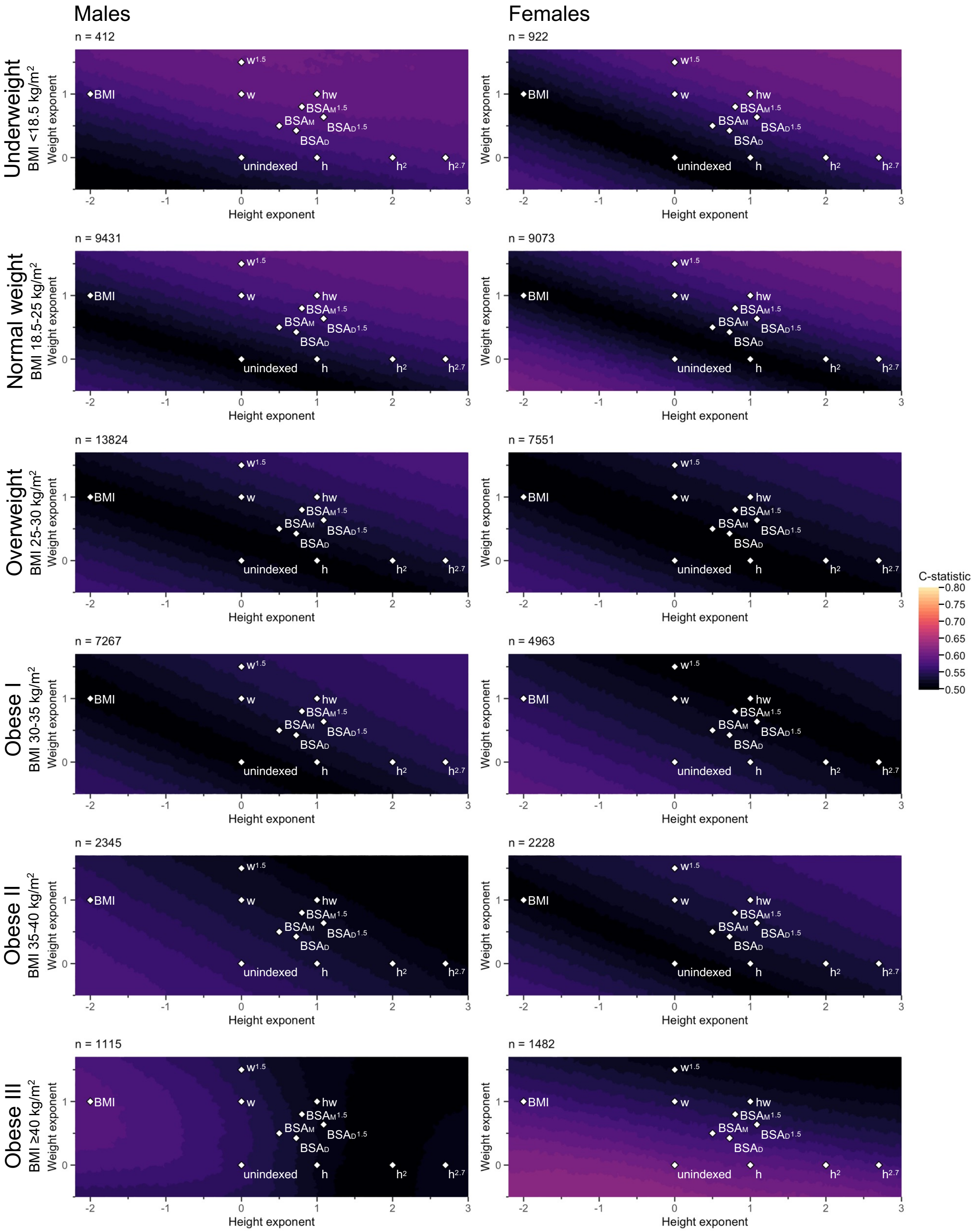

Supplementary Figure 1. Average prognostic strength of indexing for body size in aorta at sinotubular diameter.

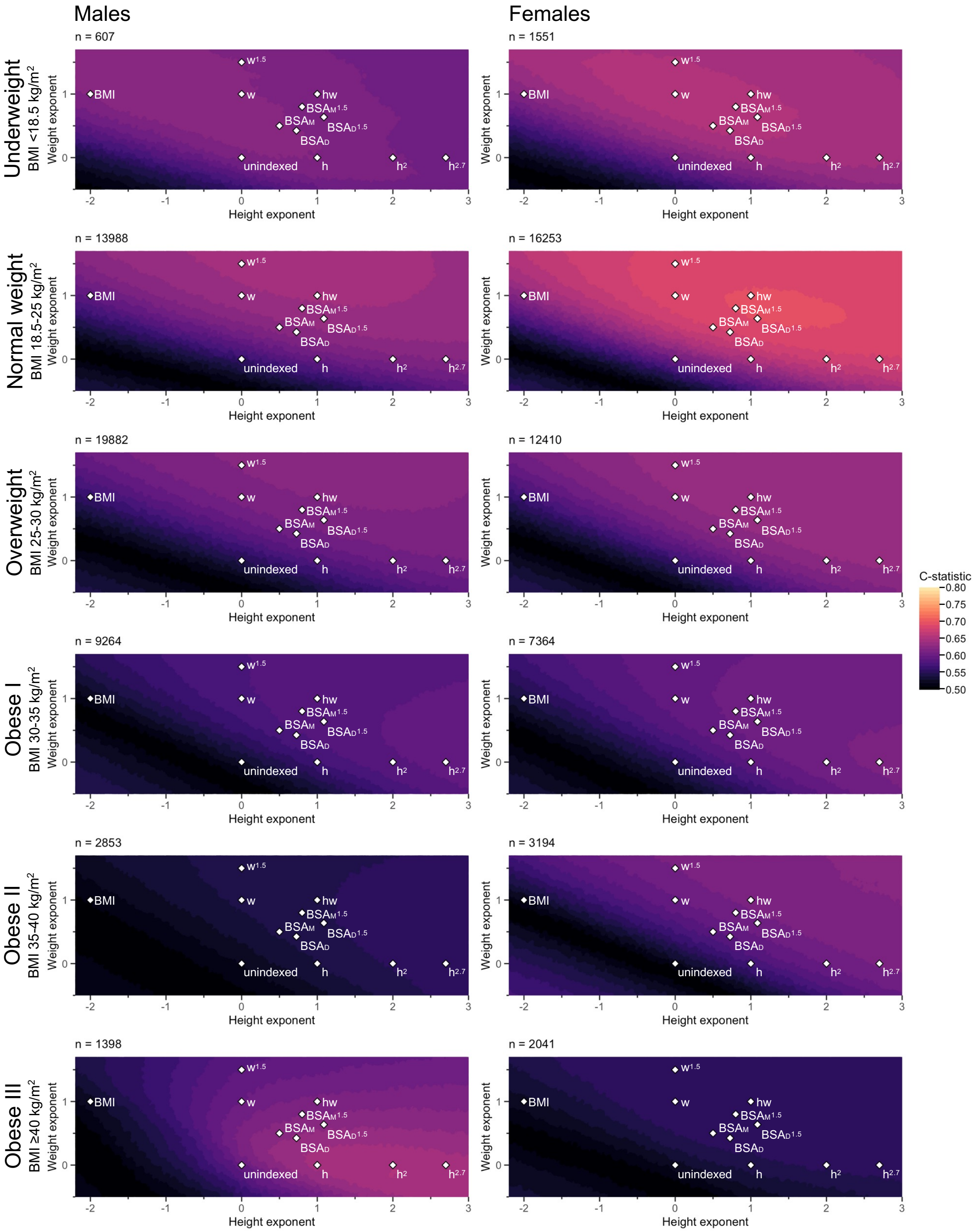

Supplementary Figure 2. Average prognostic strength of indexing for body size in aortic sinus diameter.

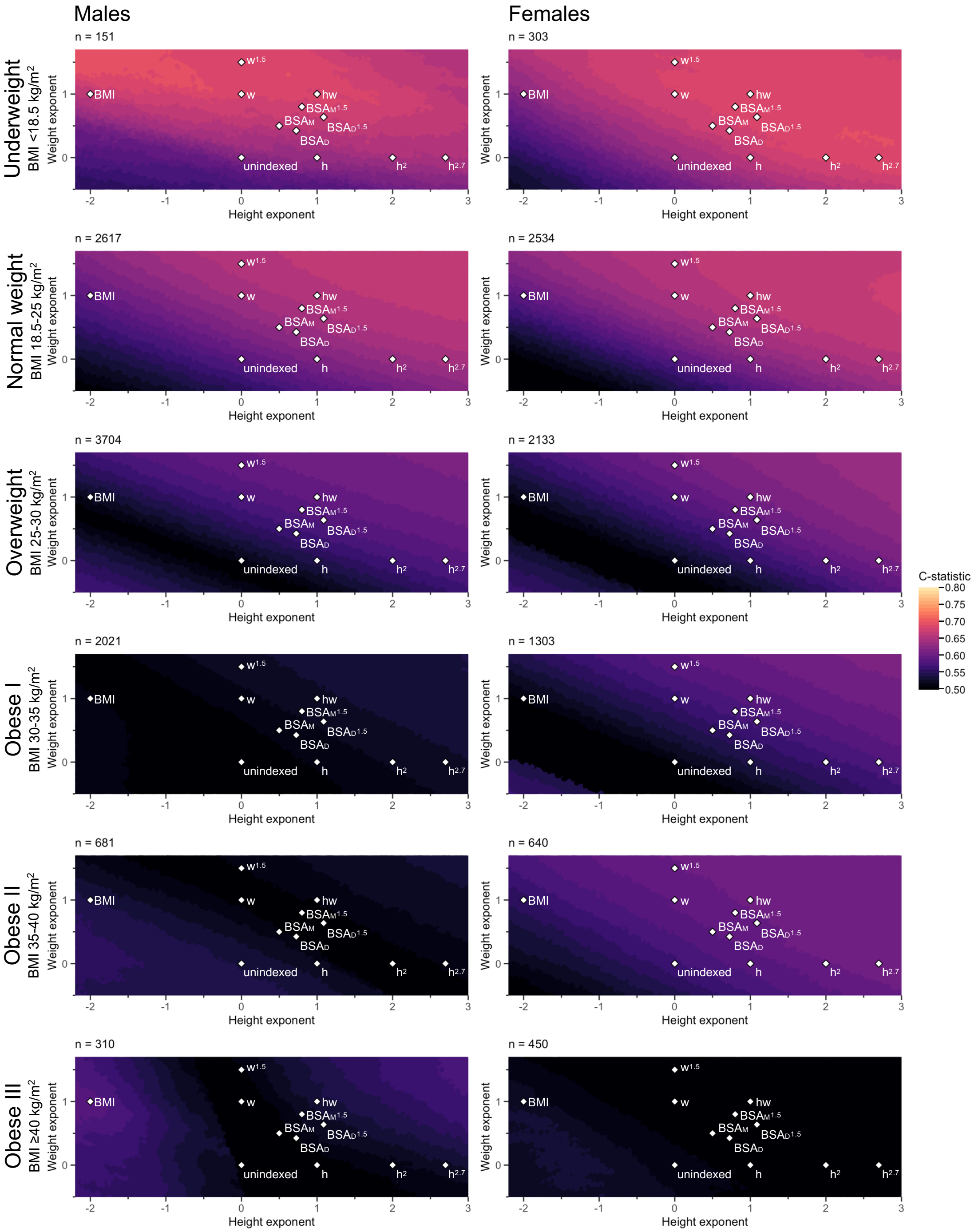

Supplementary Figure 3. Average prognostic strength of indexing for body size in aortic arch diameter.

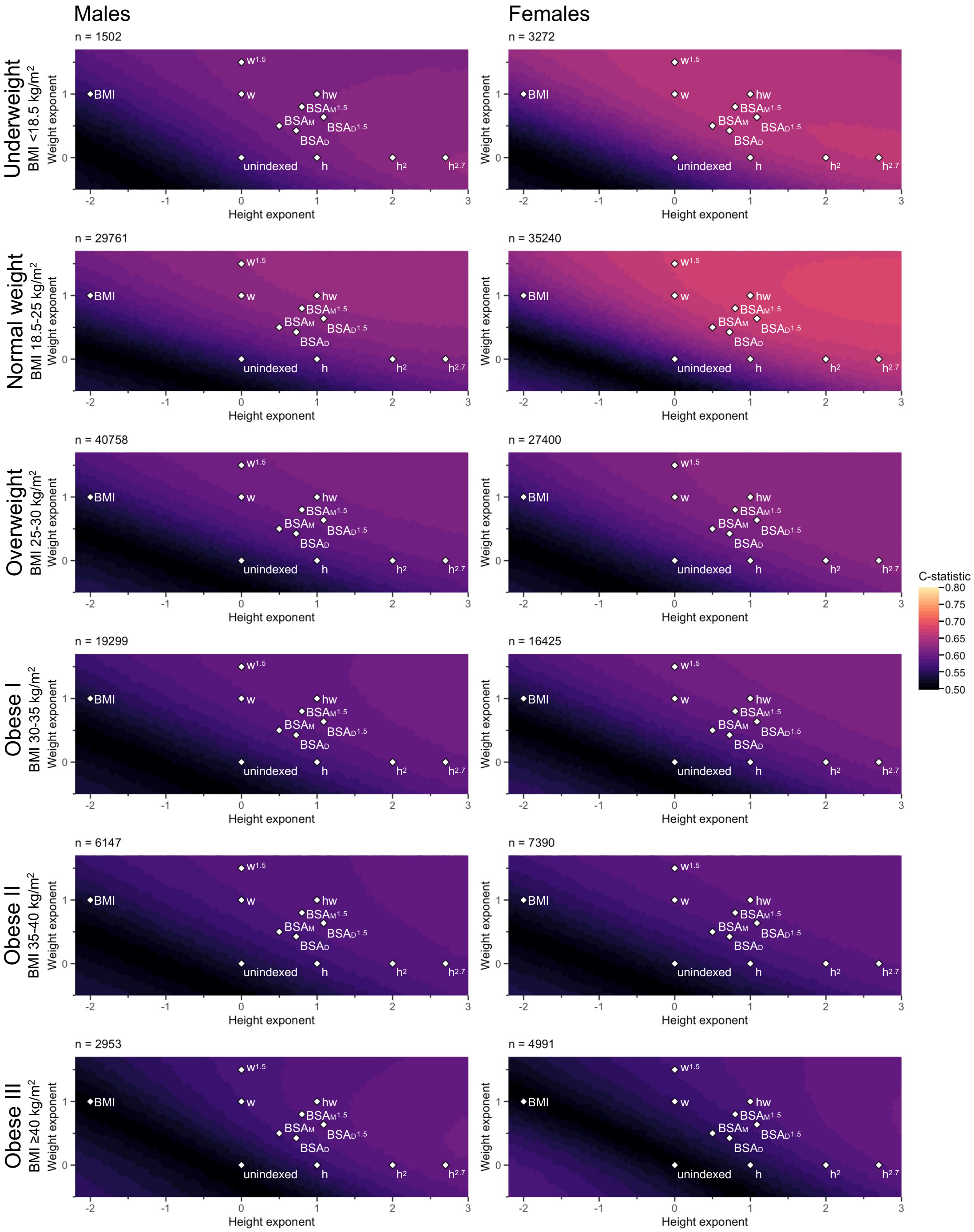

Supplementary Figure 4. Average prognostic strength of indexing for body size in aortic root diameter.

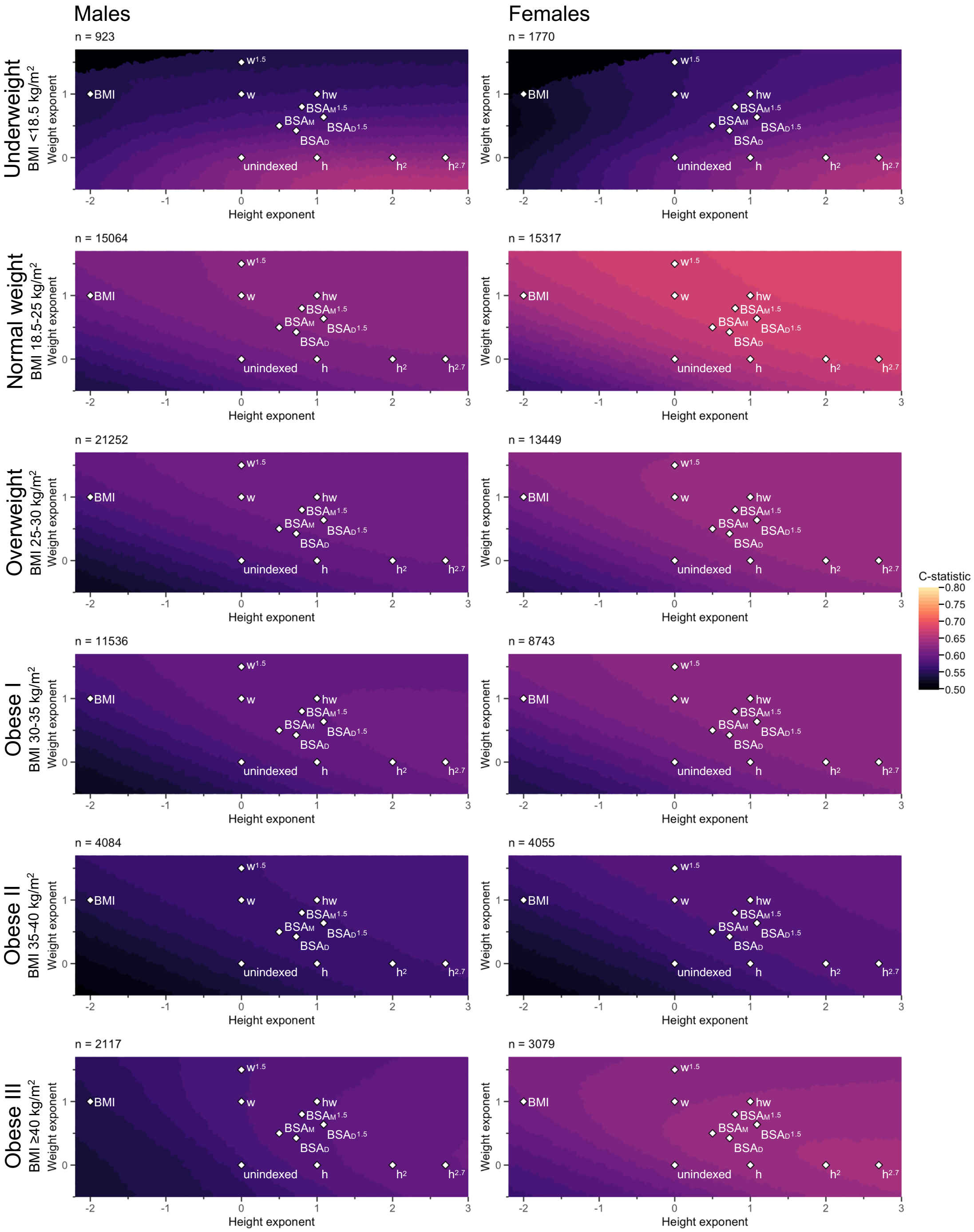

Supplementary Figure 5. Average prognostic strength of indexing for body size in ascending aorta diameter.

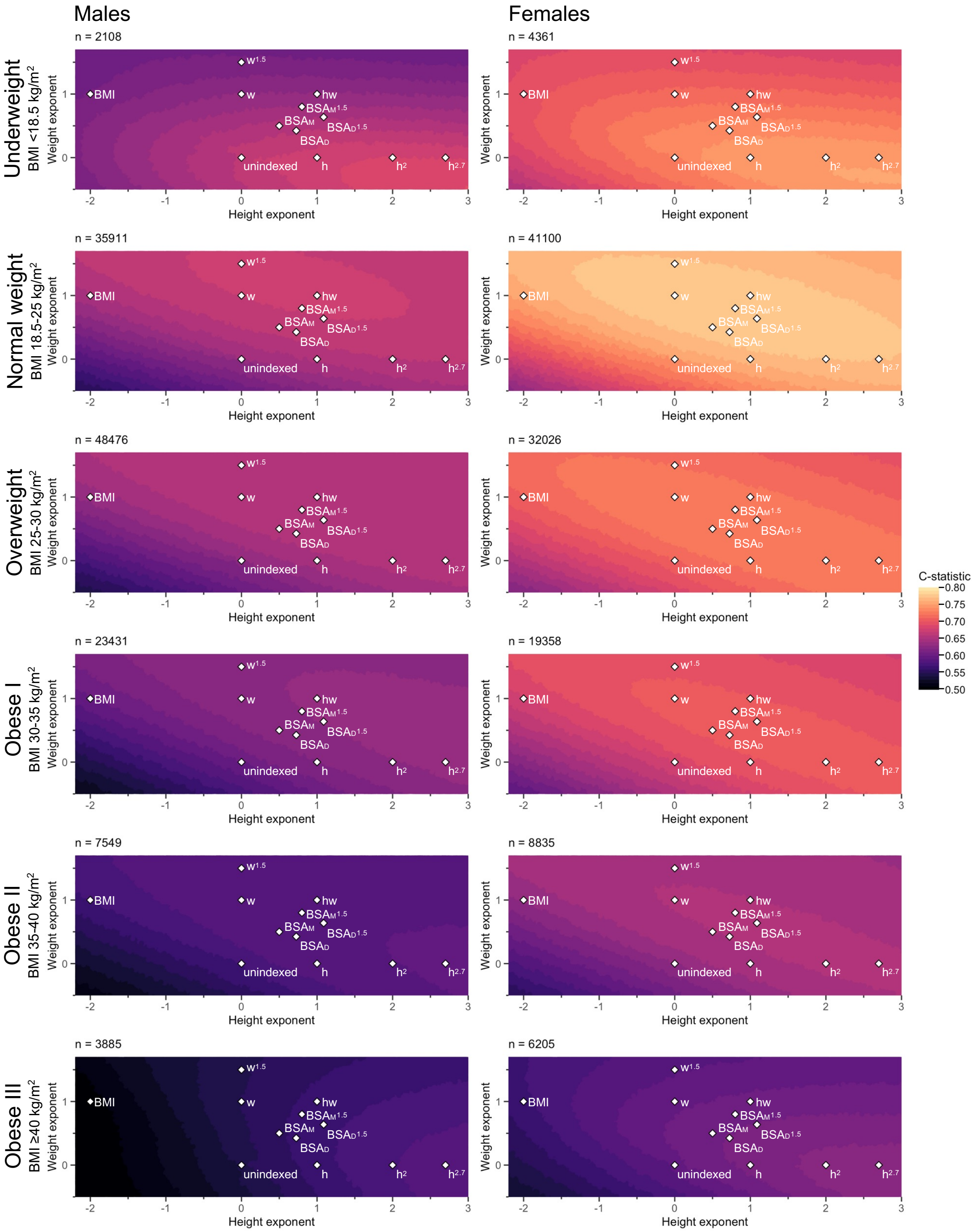

Supplementary Figure 6. Average prognostic strength of indexing for body size in IVS thickness.

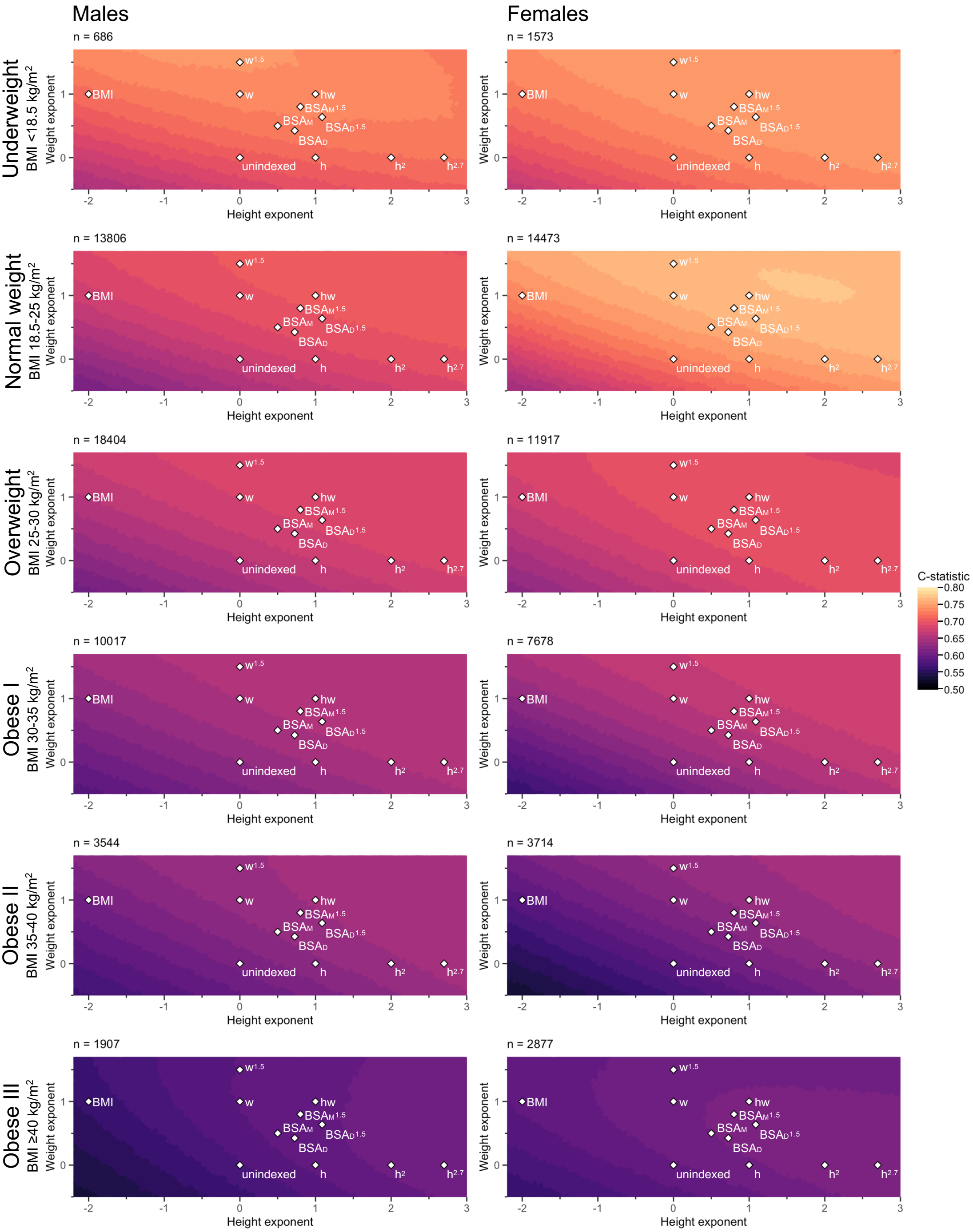

Supplementary Figure 7. Average prognostic strength of indexing for body size in LA area.

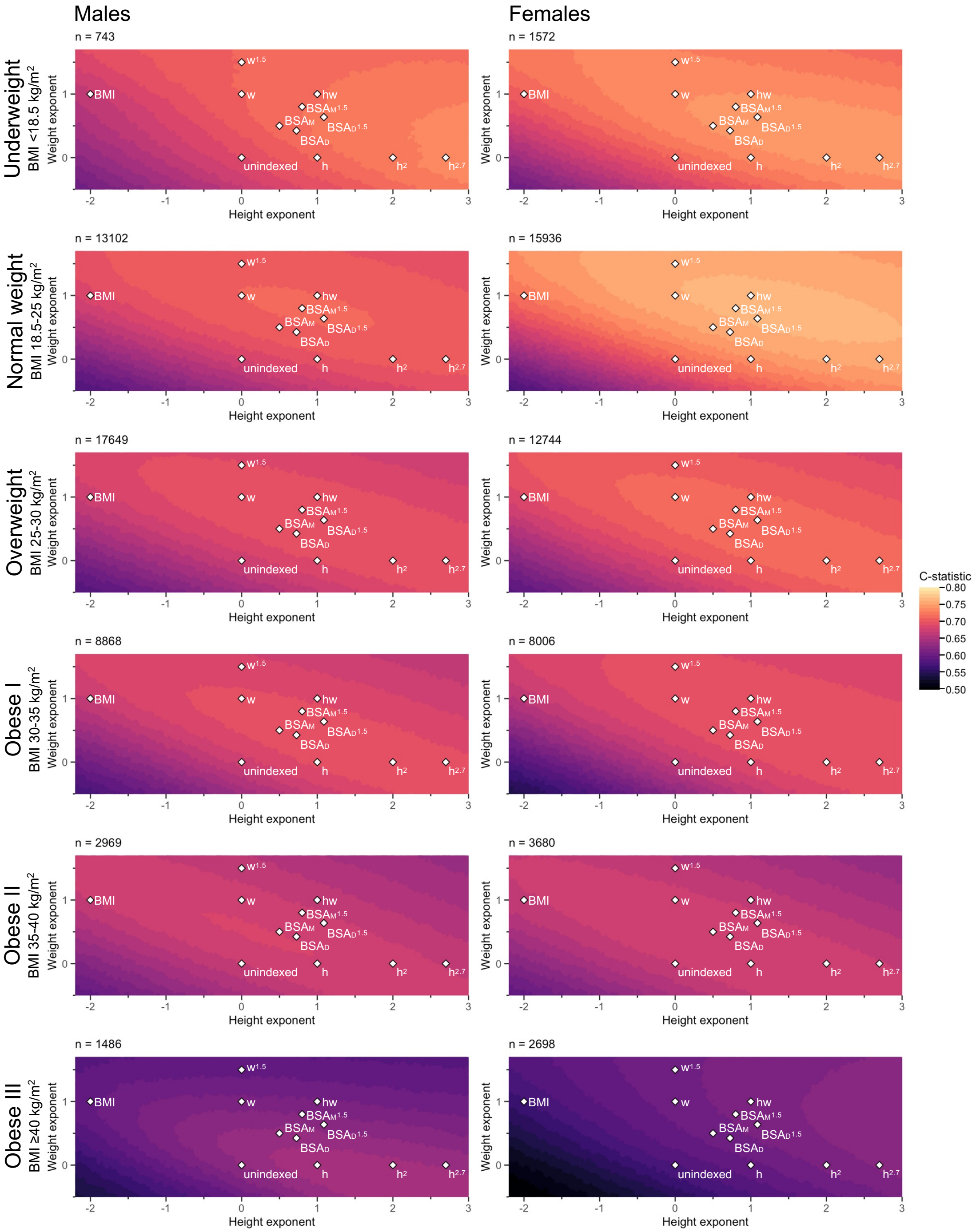

Supplementary Figure 8. Average prognostic strength of indexing for body size in LA diameter.

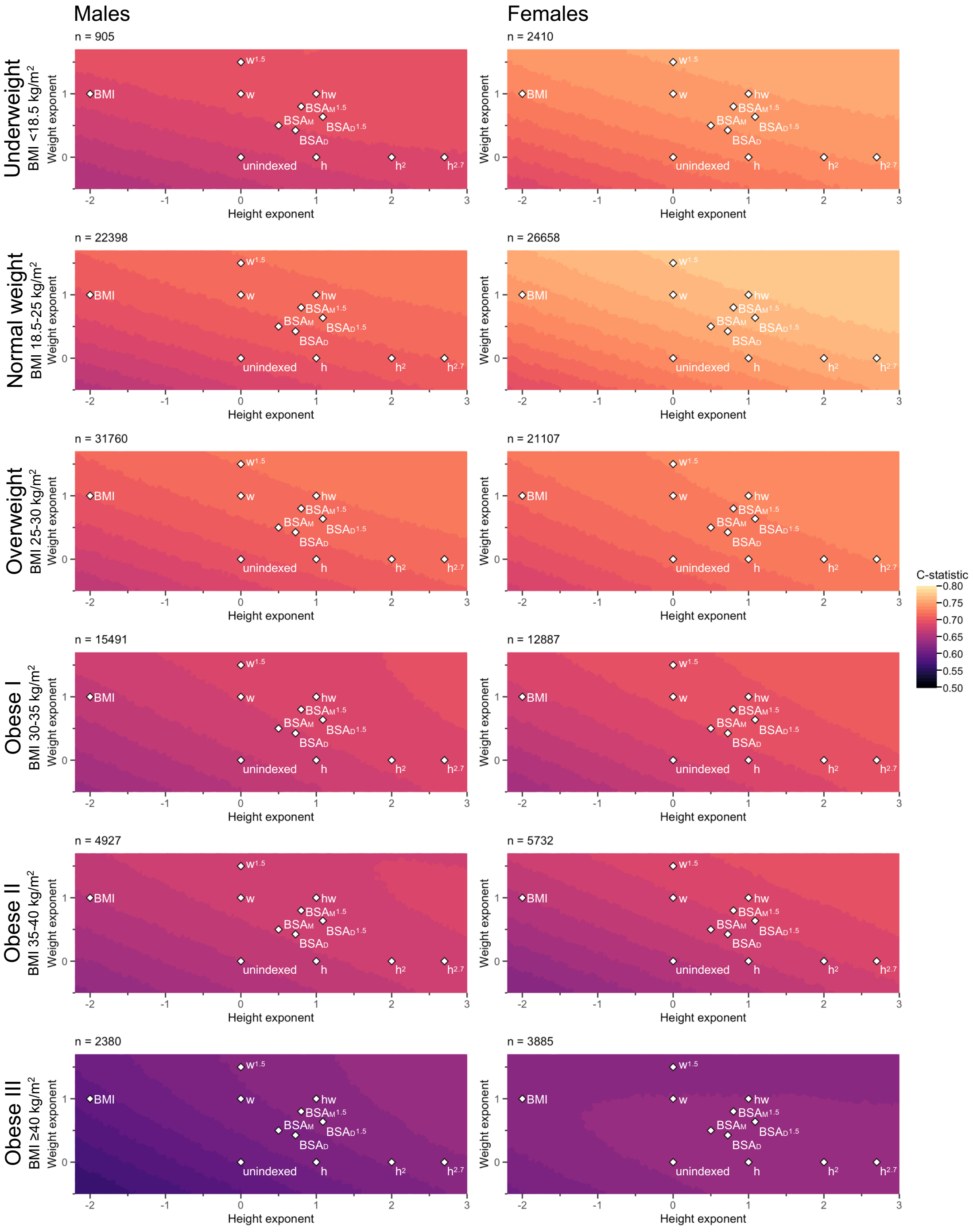

Supplementary Figure 9. Average prognostic strength of indexing for body size in LA volume.

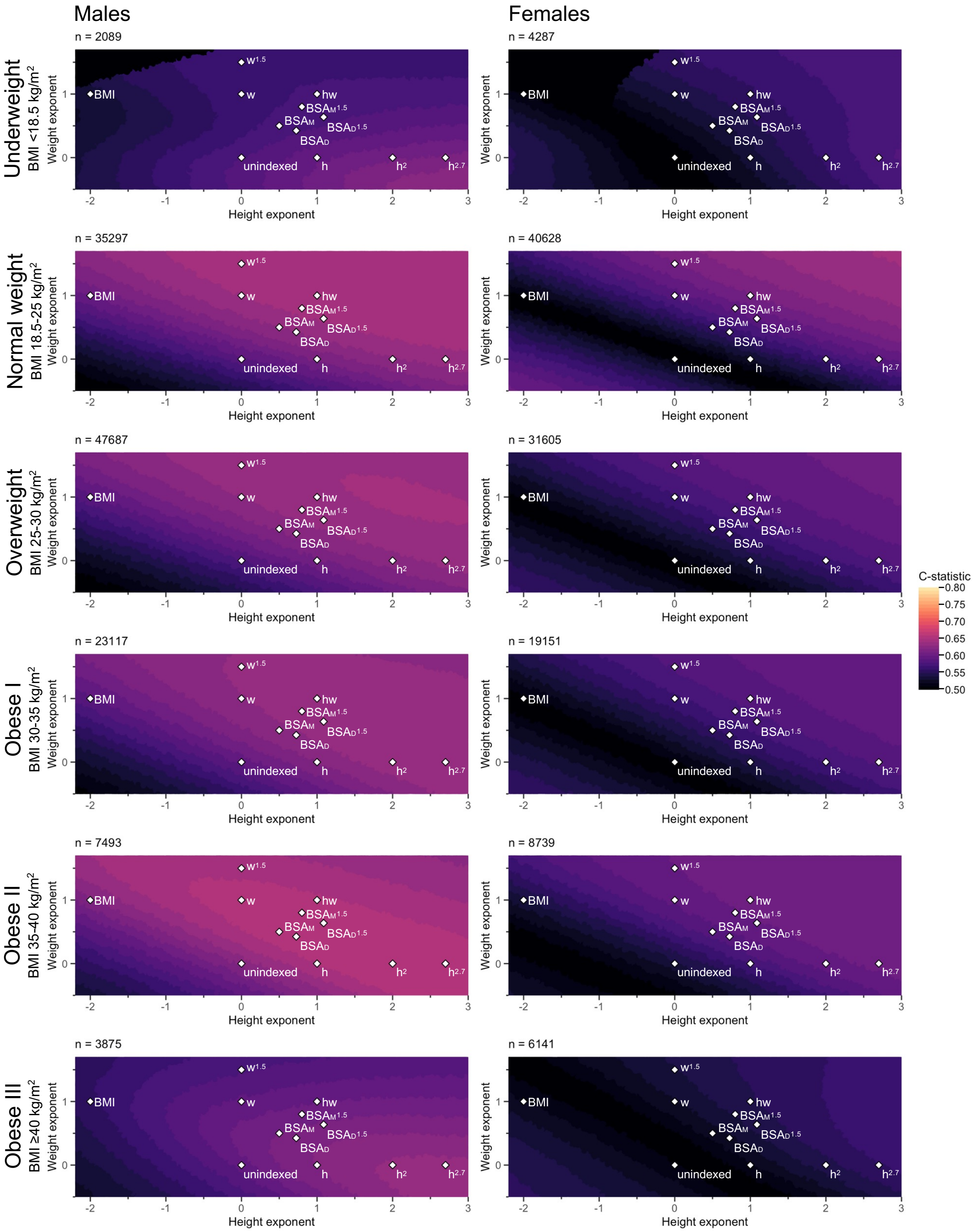

Supplementary Figure 10. Average prognostic strength of indexing for body size in LV end-diastolic diameter.

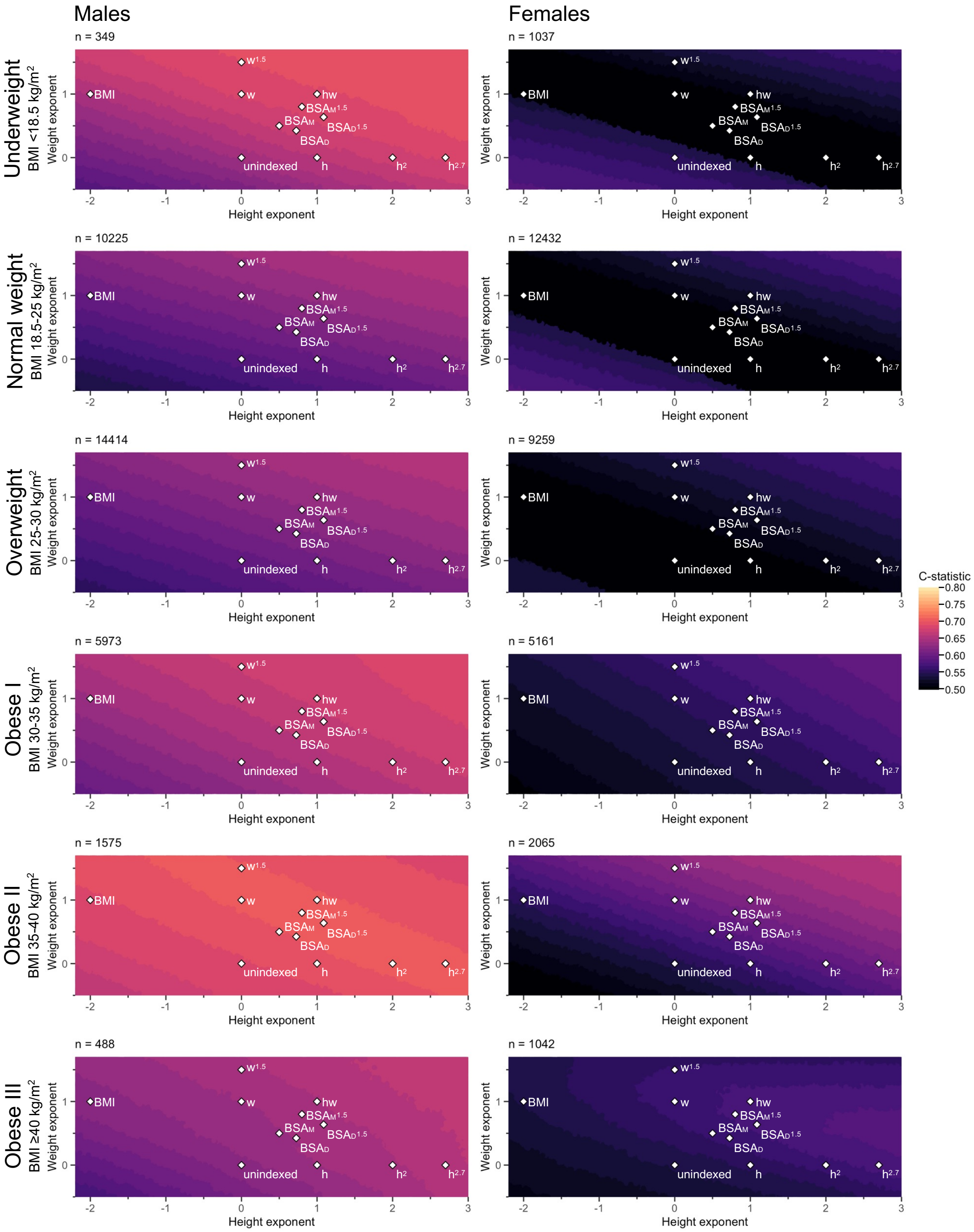

Supplementary Figure 11. Average prognostic strength of indexing for body size in LV end-diastolic volume.

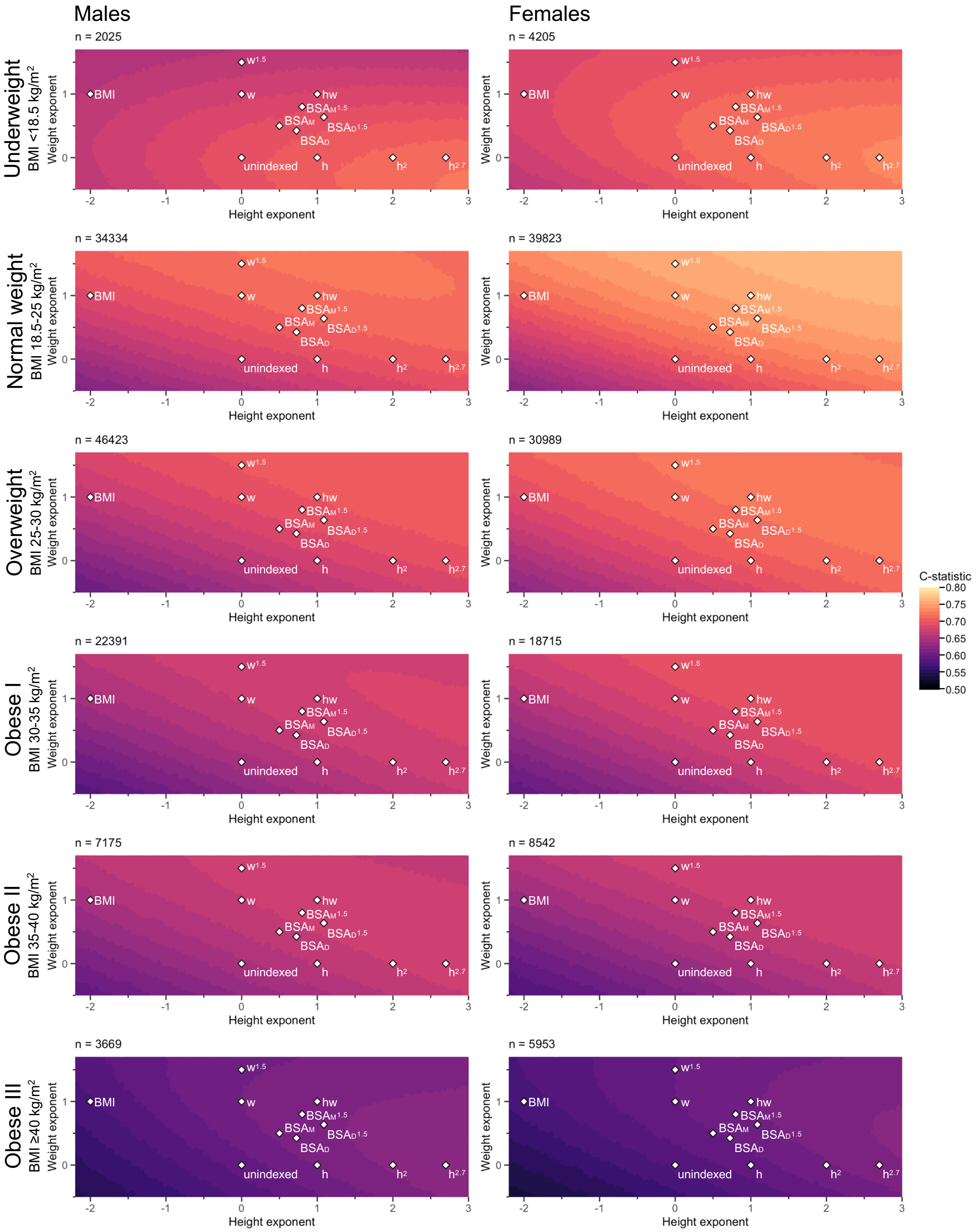

Supplementary Figure 12. Average prognostic strength of indexing for body size in LV mass.

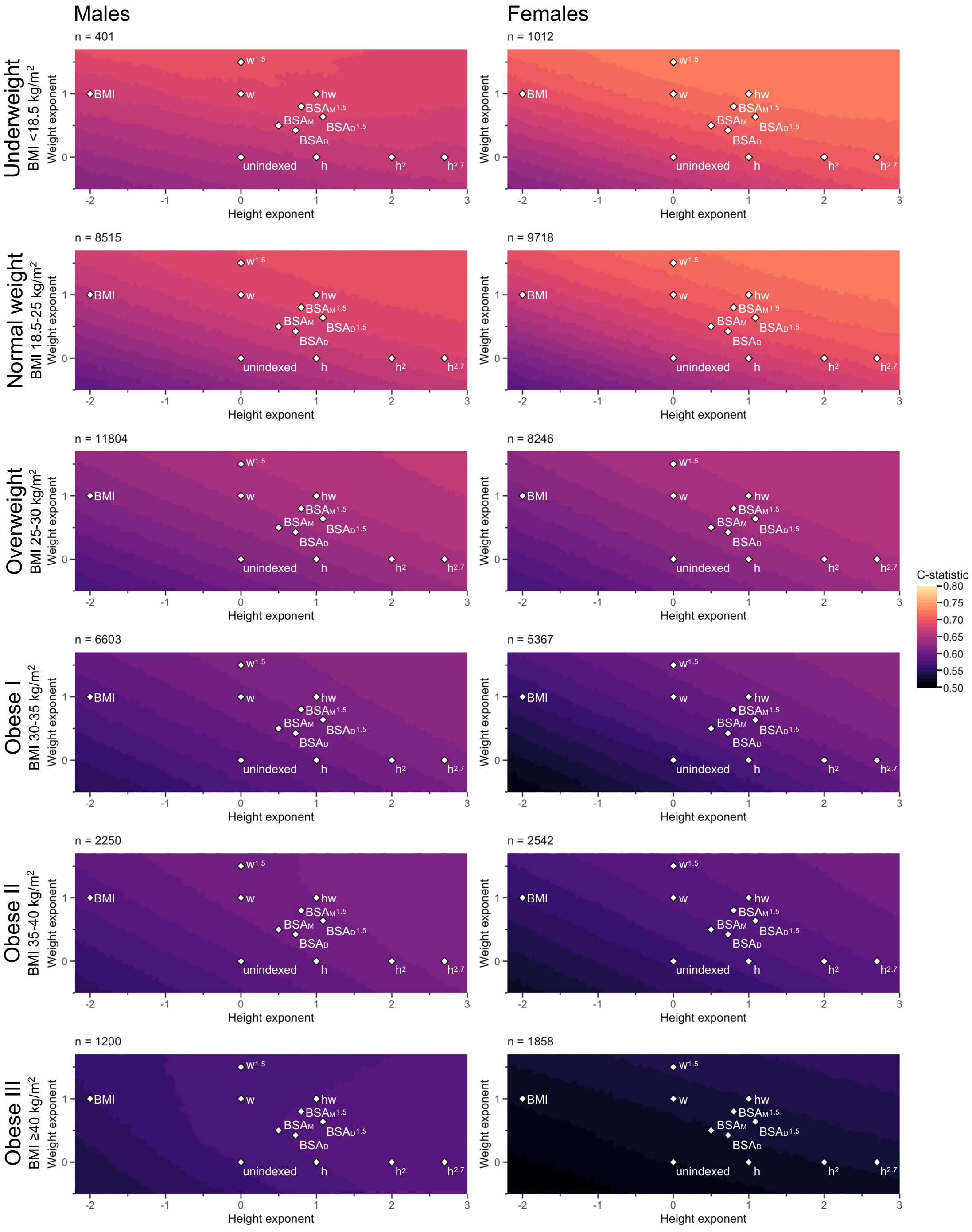

Supplementary Figure 13. Average prognostic strength of indexing for body size in RA area.

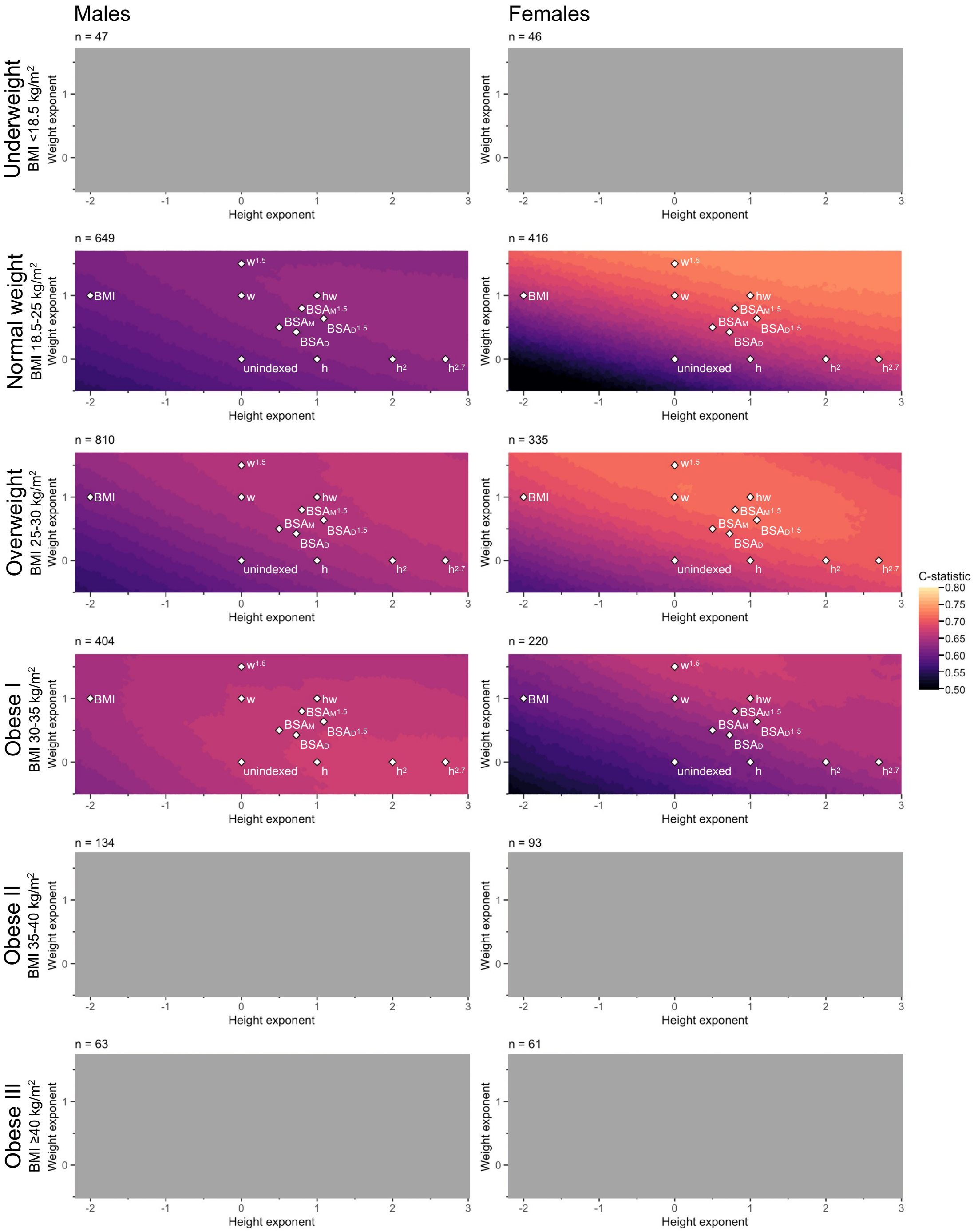

Supplementary Figure 14. Average prognostic strength of indexing for body size in RV diameter. Data for BMI <18.5 and ≥ 30 kg/m^2 are not shown due to insufficient sample sizes.

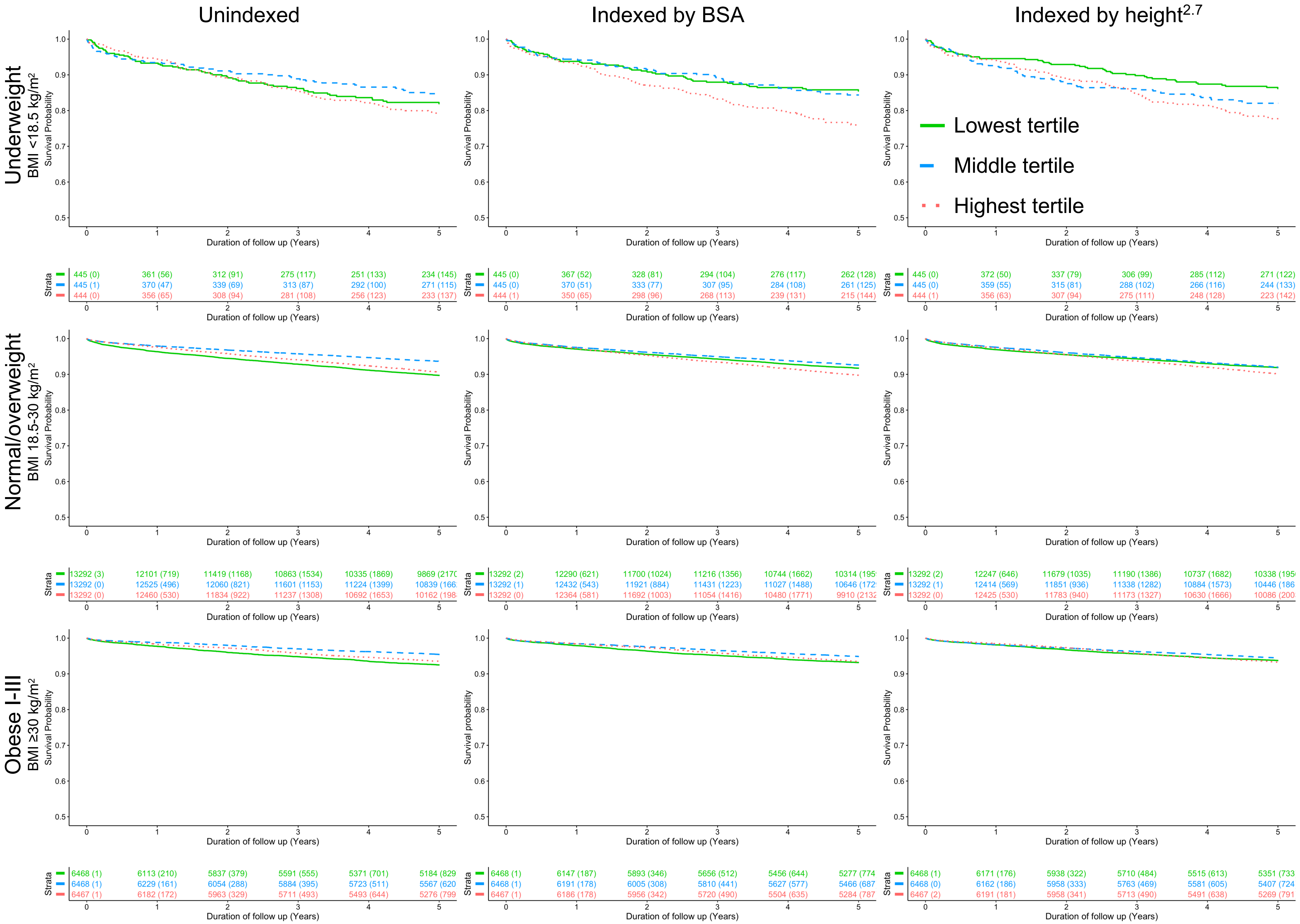

Supplementary Figure 15. Kaplan-Meir curves for 5-year cardiovascular mortality for aorta at sinotubular diameter unindexed, indexed by BSA and indexed by height$^{2.7}$.

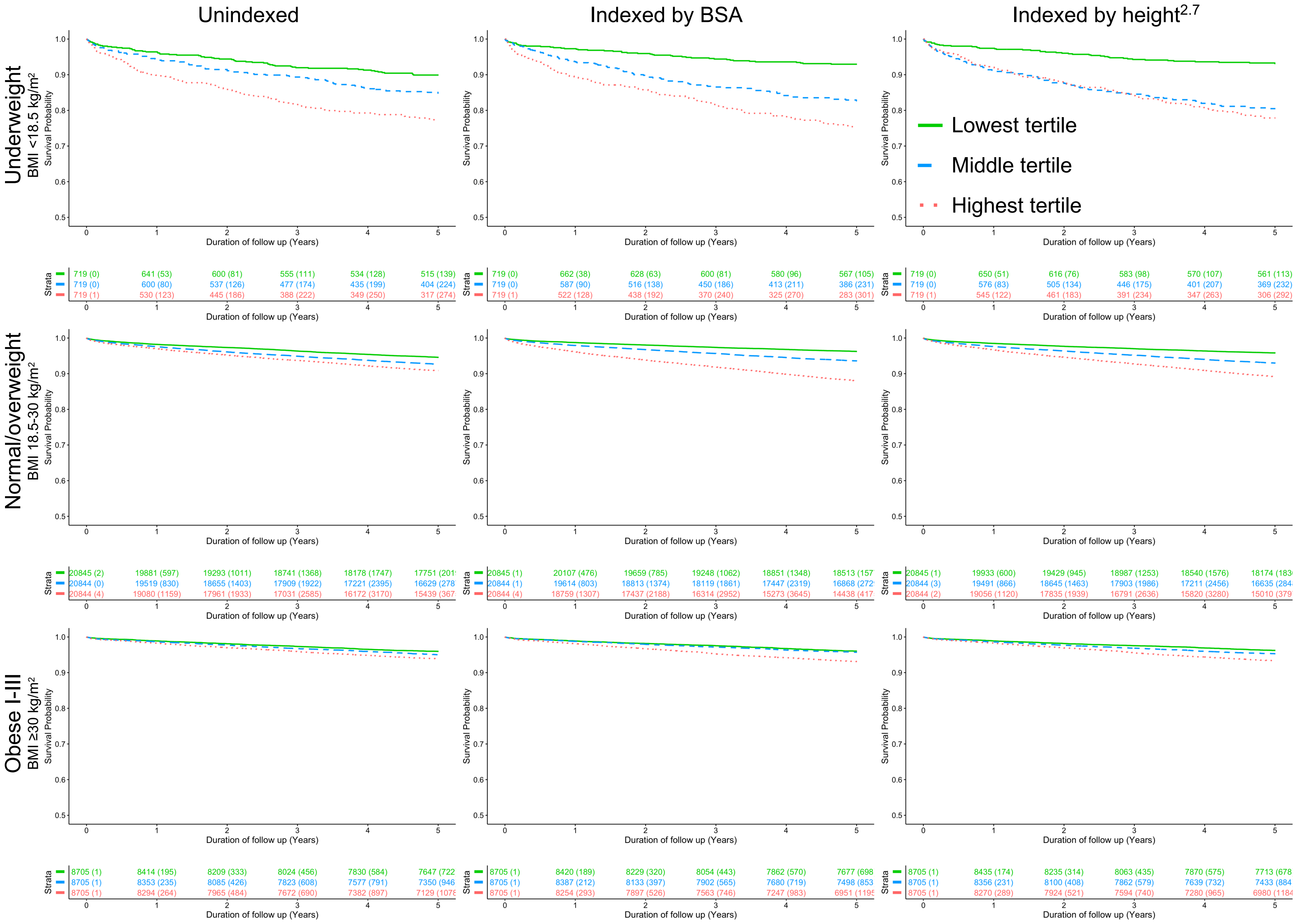

Supplementary Figure 16. Kaplan-Meir curves for 5-year cardiovascular mortality for aortic sinus diameter unindexed, indexed by BSA and indexed by height$^{2.7}$.

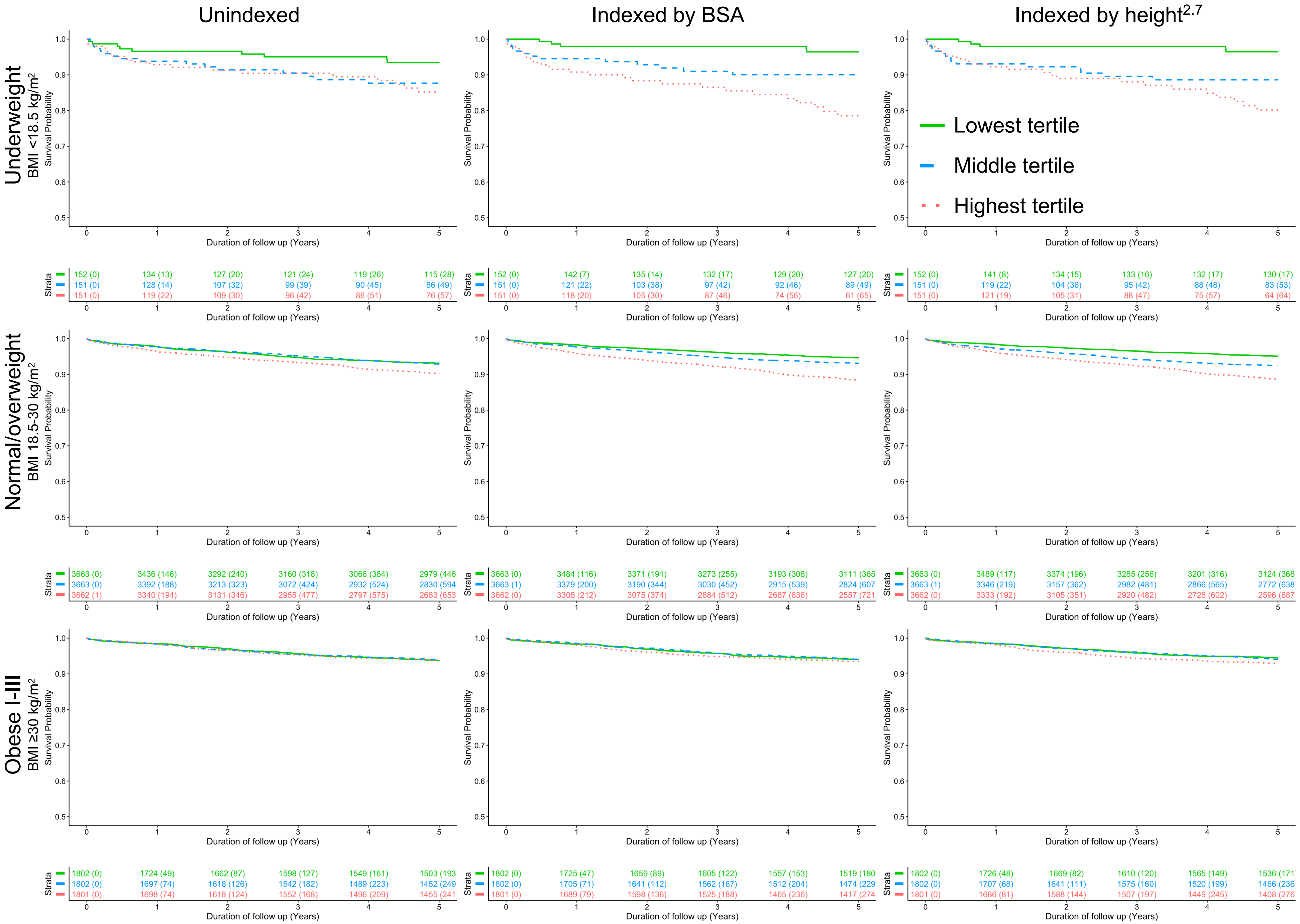

Supplementary Figure 17. Kaplan-Meir curves for 5-year cardiovascular mortality for aortic arch diameter unindexed, indexed by BSA and indexed by height$^{2.7}$.

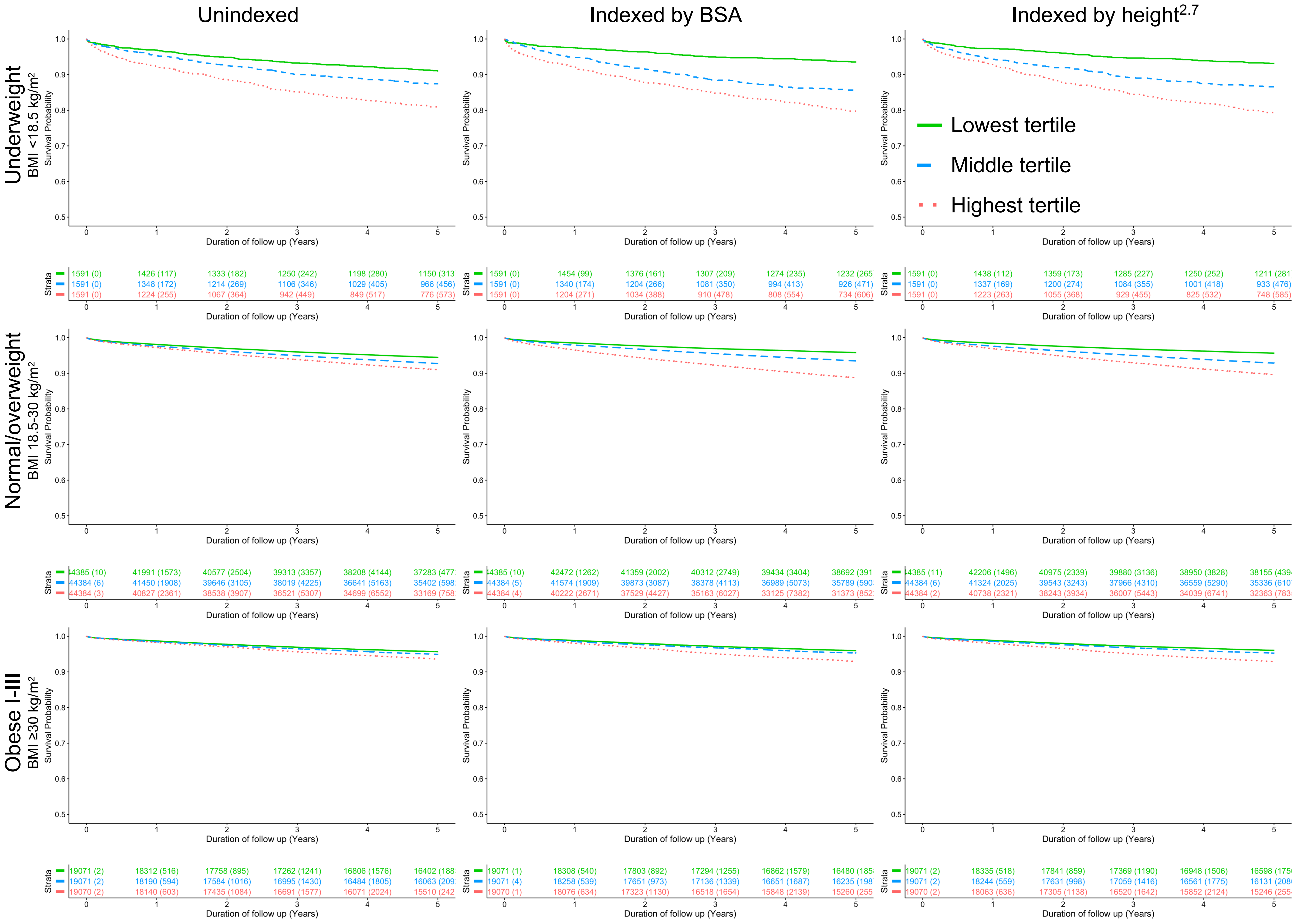

Supplementary Figure 18. Kaplan-Meir curves for 5-year cardiovascular mortality for aortic root diameter unindexed, indexed by BSA and indexed by height$^{2.7}$.

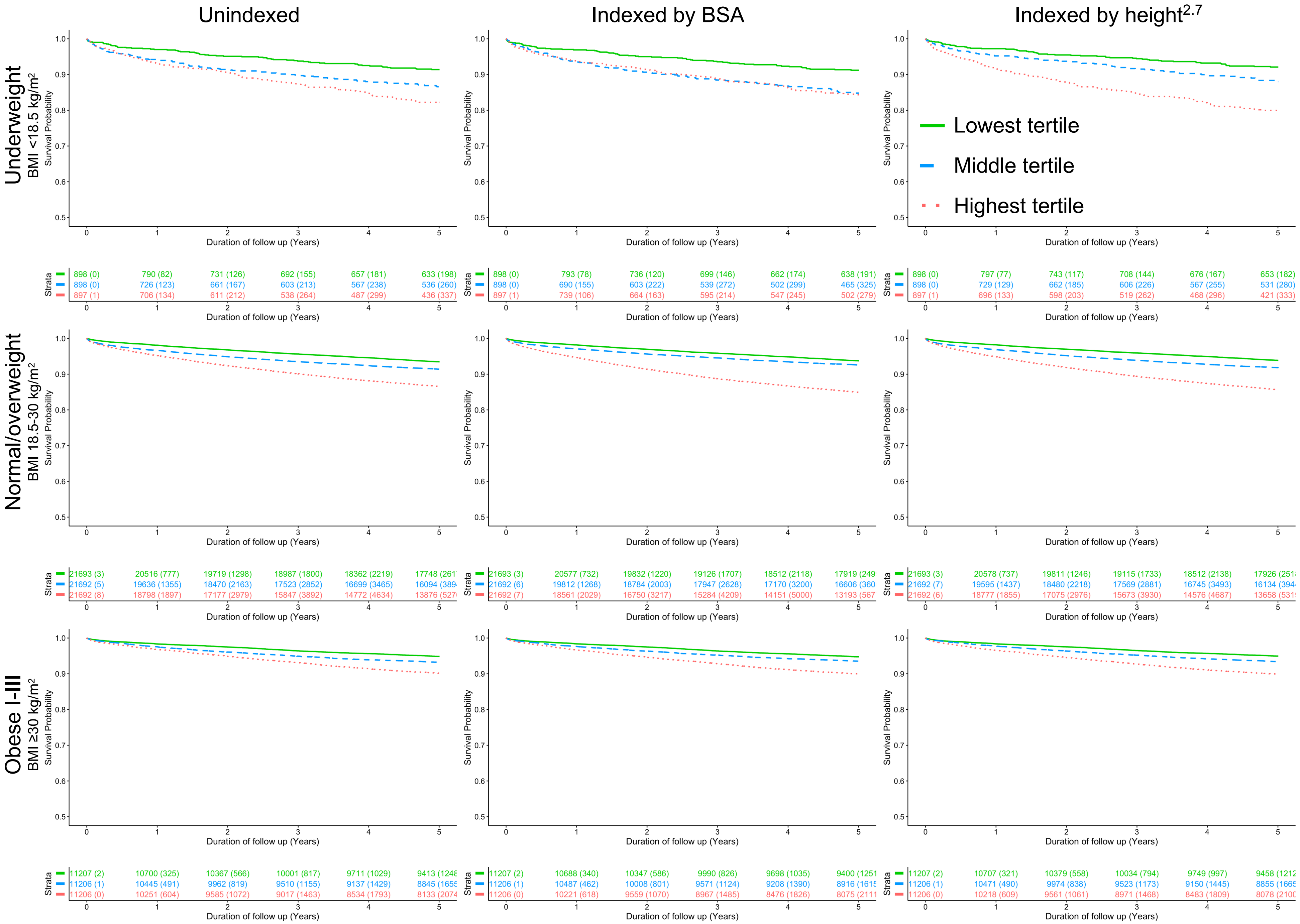

Supplementary Figure 19. Kaplan-Meir curves for 5-year cardiovascular mortality for ascending aorta diameter unindexed, indexed by BSA and indexed by height$^{2.7}$.

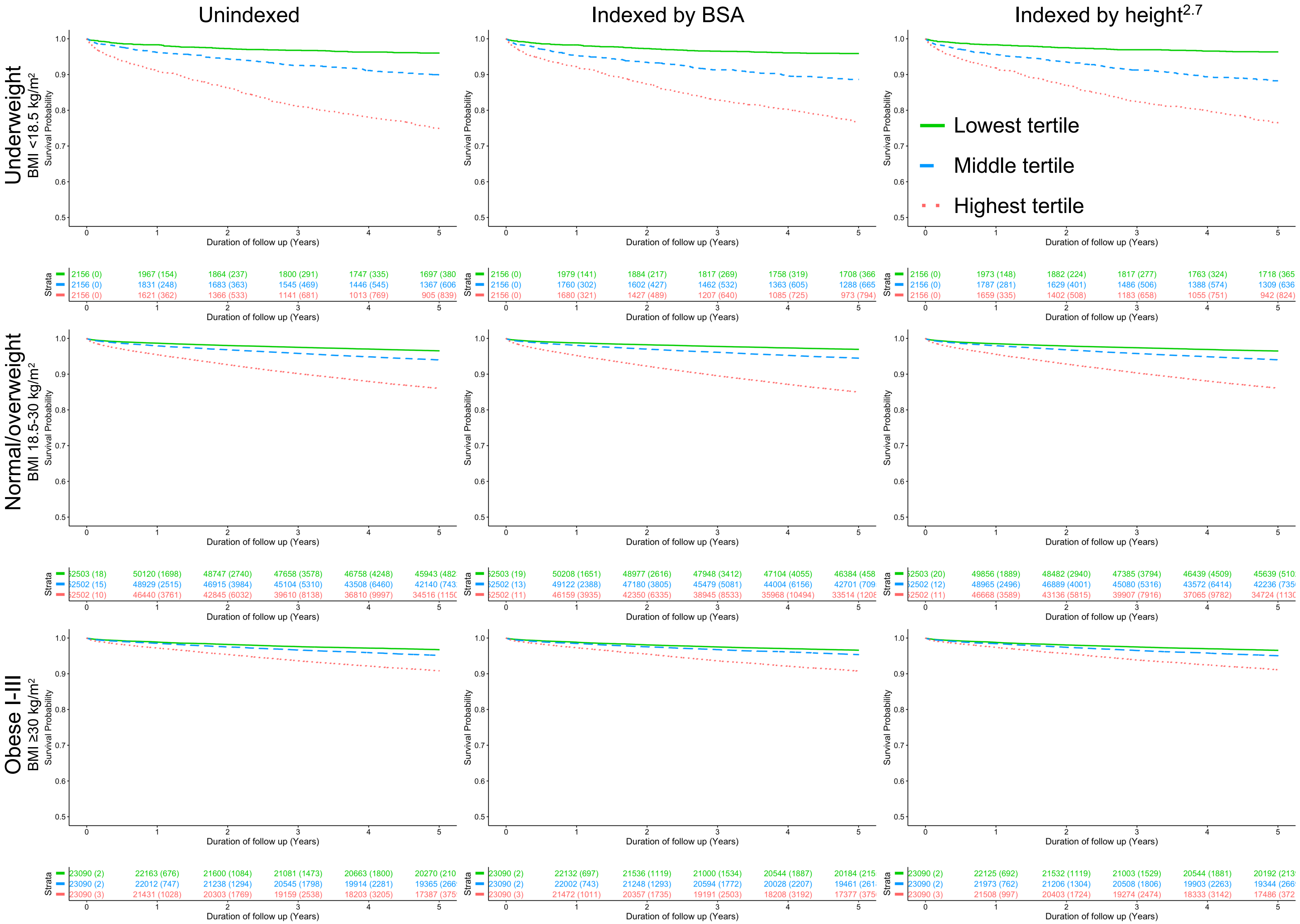

Supplementary Figure 20. Kaplan-Meir curves for 5-year cardiovascular mortality for IVS thickness unindexed, indexed by BSA and indexed by height$^{2.7}$.

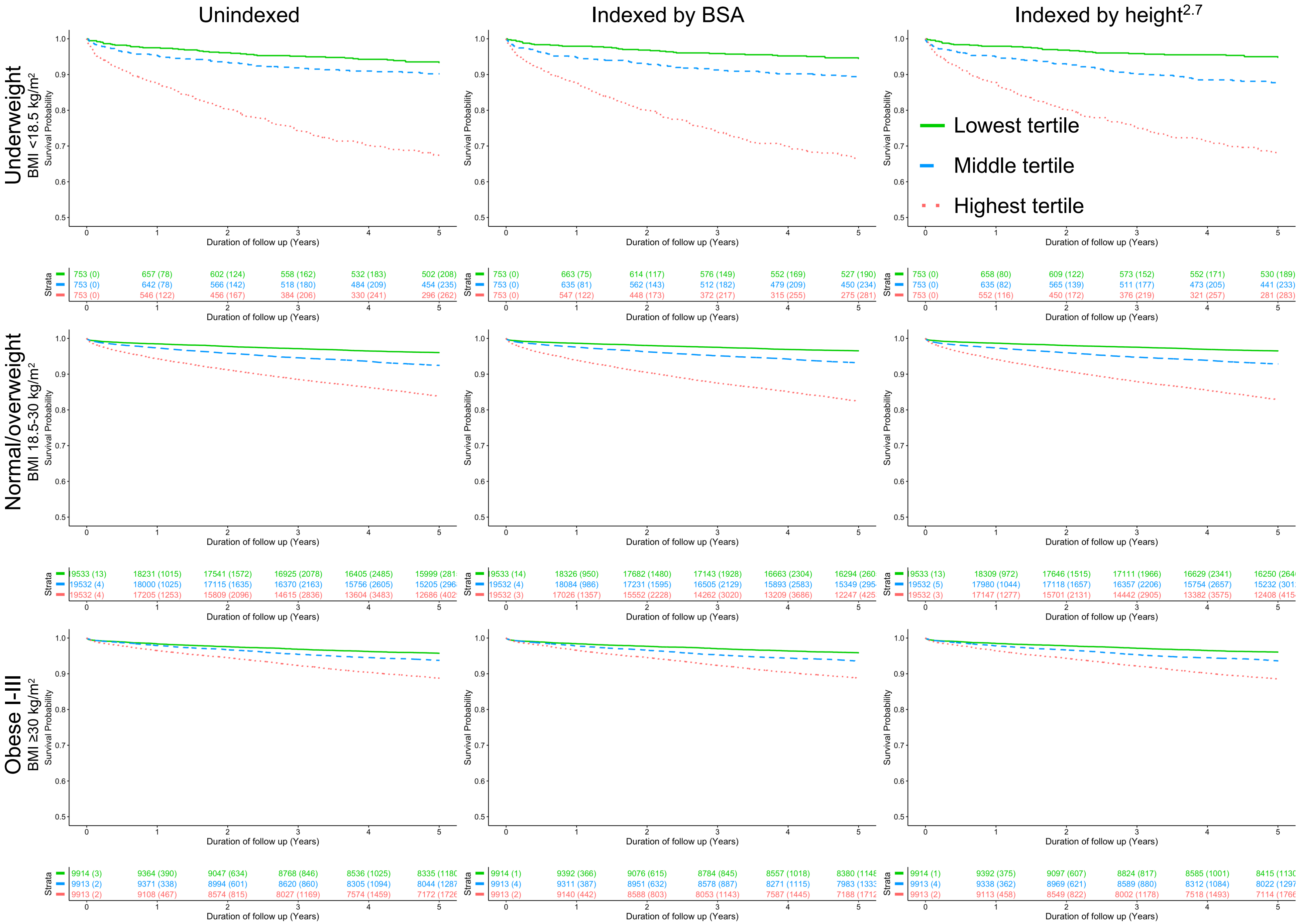

Supplementary Figure 21. Kaplan-Meir curves for 5-year cardiovascular mortality for LA area unindexed, indexed by BSA and indexed by height$^{2.7}$.

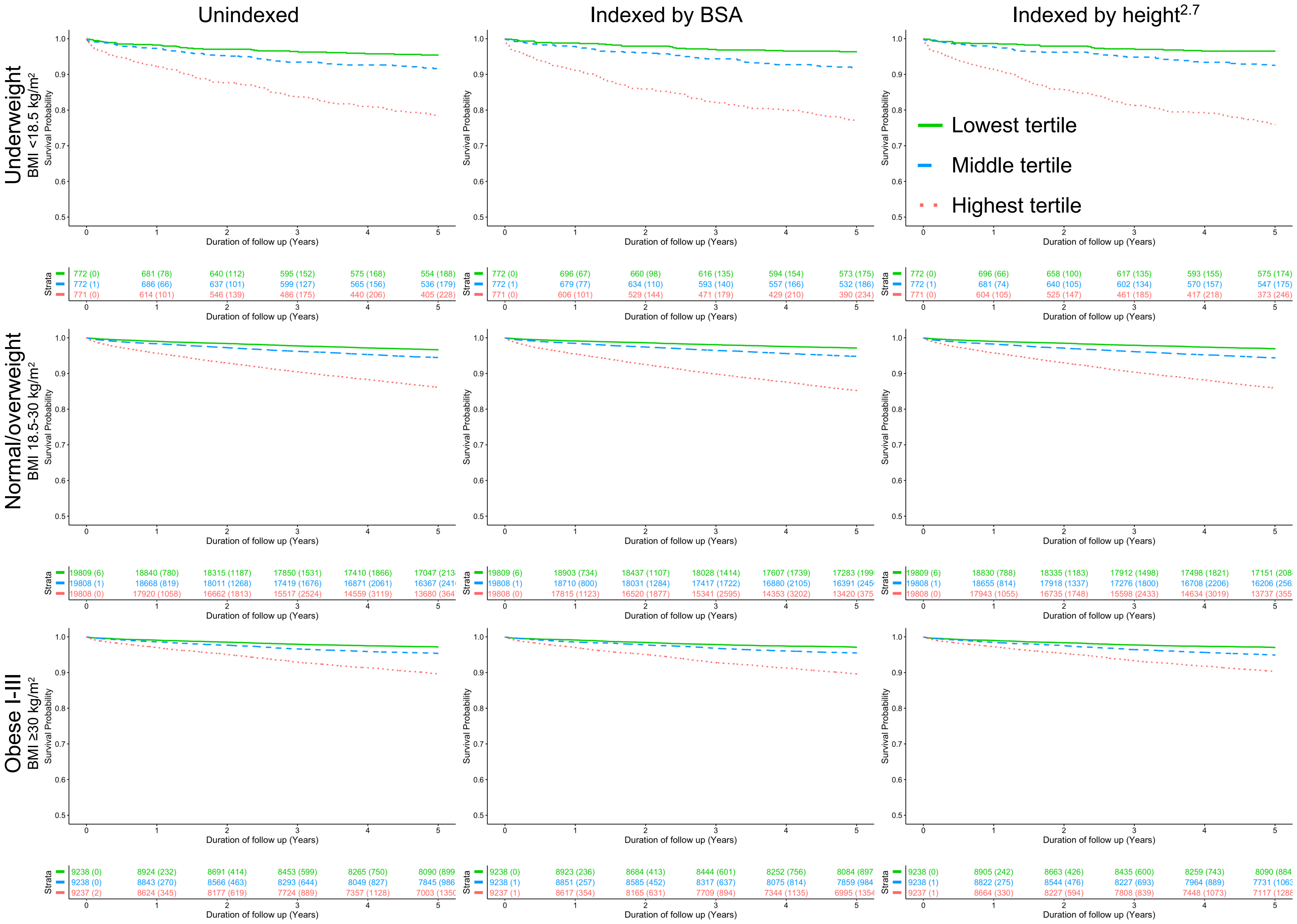

Supplementary Figure 22. Kaplan-Meir curves for 5-year cardiovascular mortality for LA diameter unindexed, indexed by BSA and indexed by height$^{2.7}$.

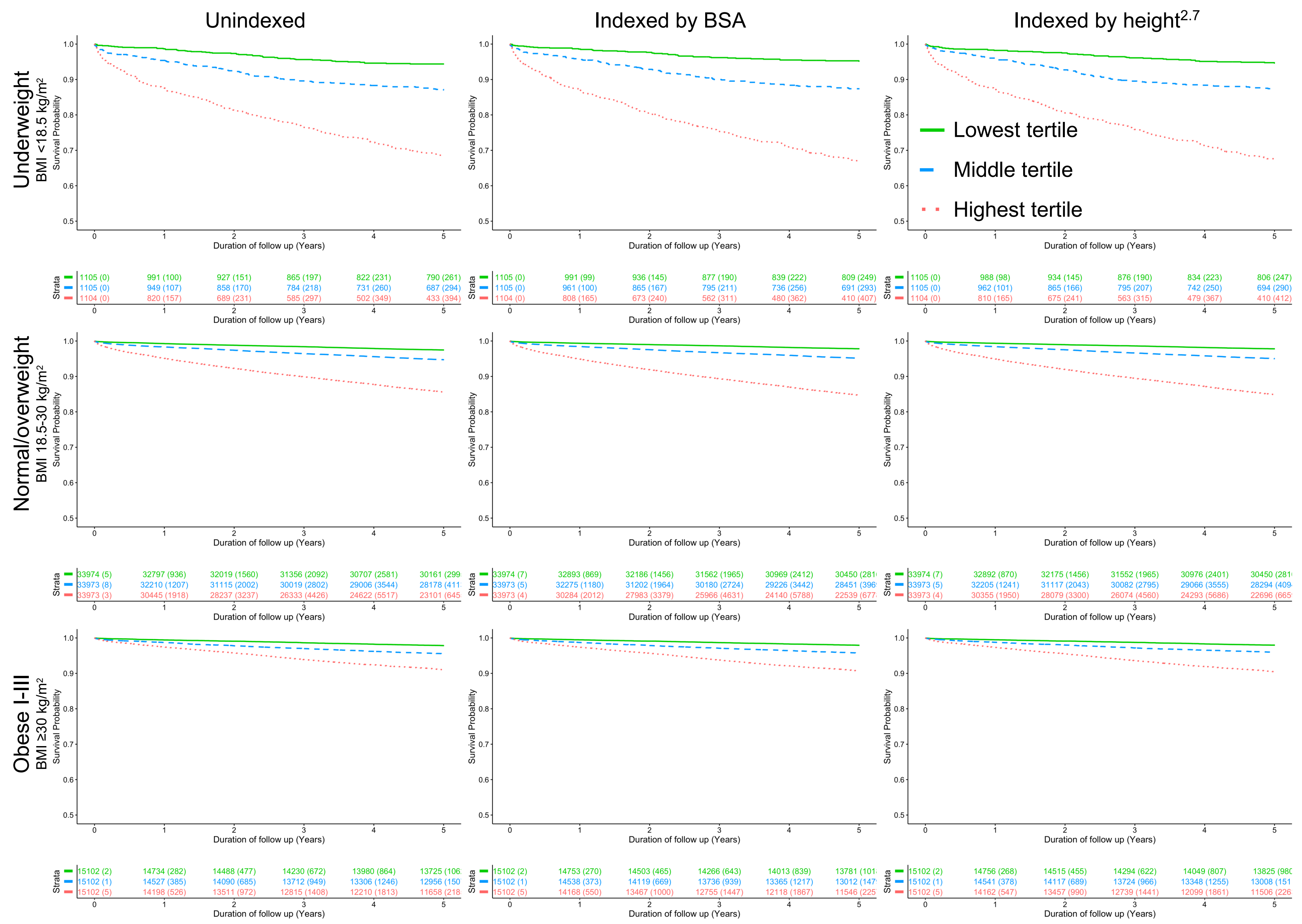

Supplementary Figure 23. Kaplan-Meir curves for 5-year cardiovascular mortality for LA volume unindexed, indexed by BSA and indexed by height$^{2.7}$.

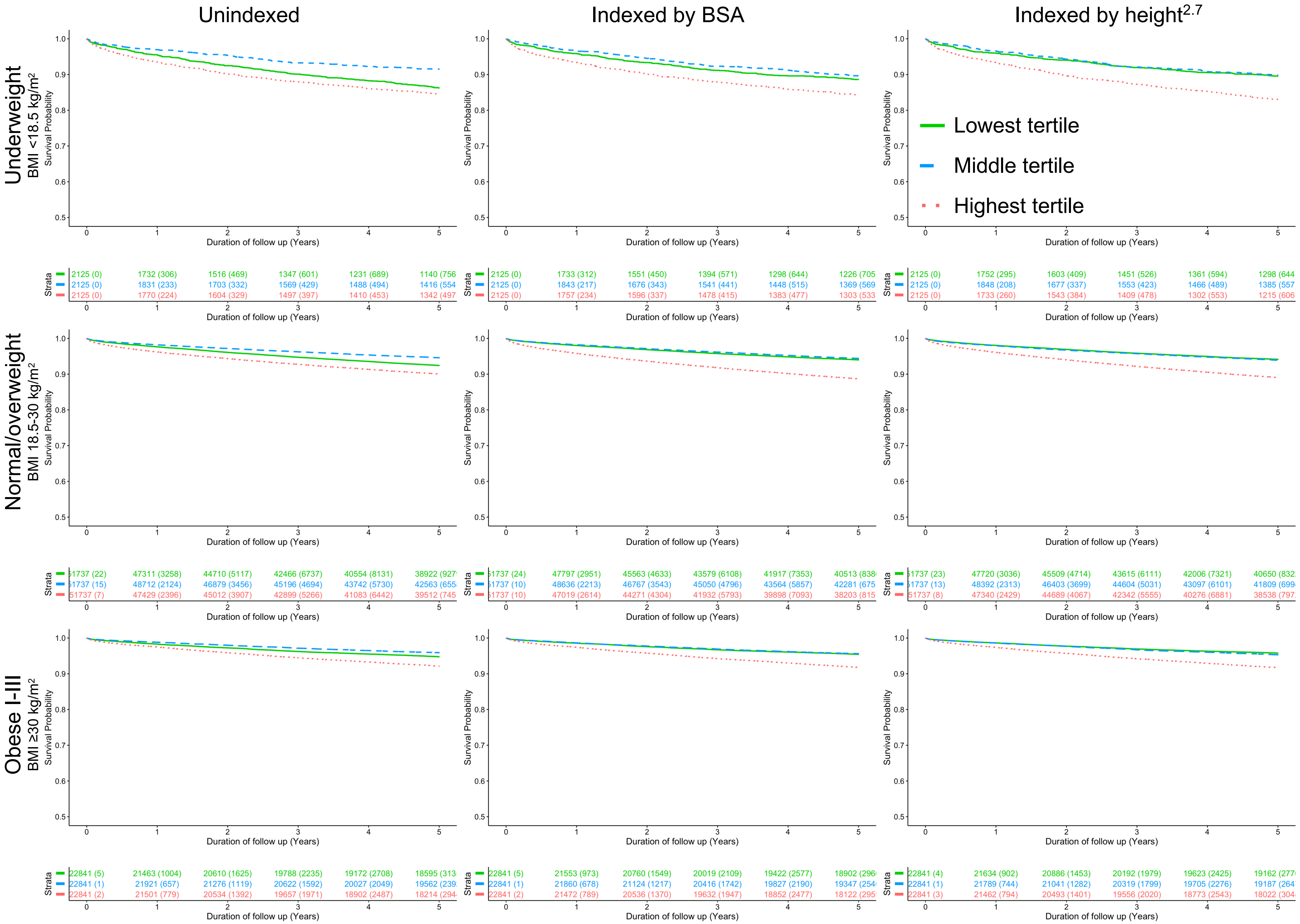

Supplementary Figure 24. Kaplan-Meir curves for 5-year cardiovascular mortality for LV end-diastolic diameter unindexed, indexed by BSA and indexed by height$^{2.7}$.

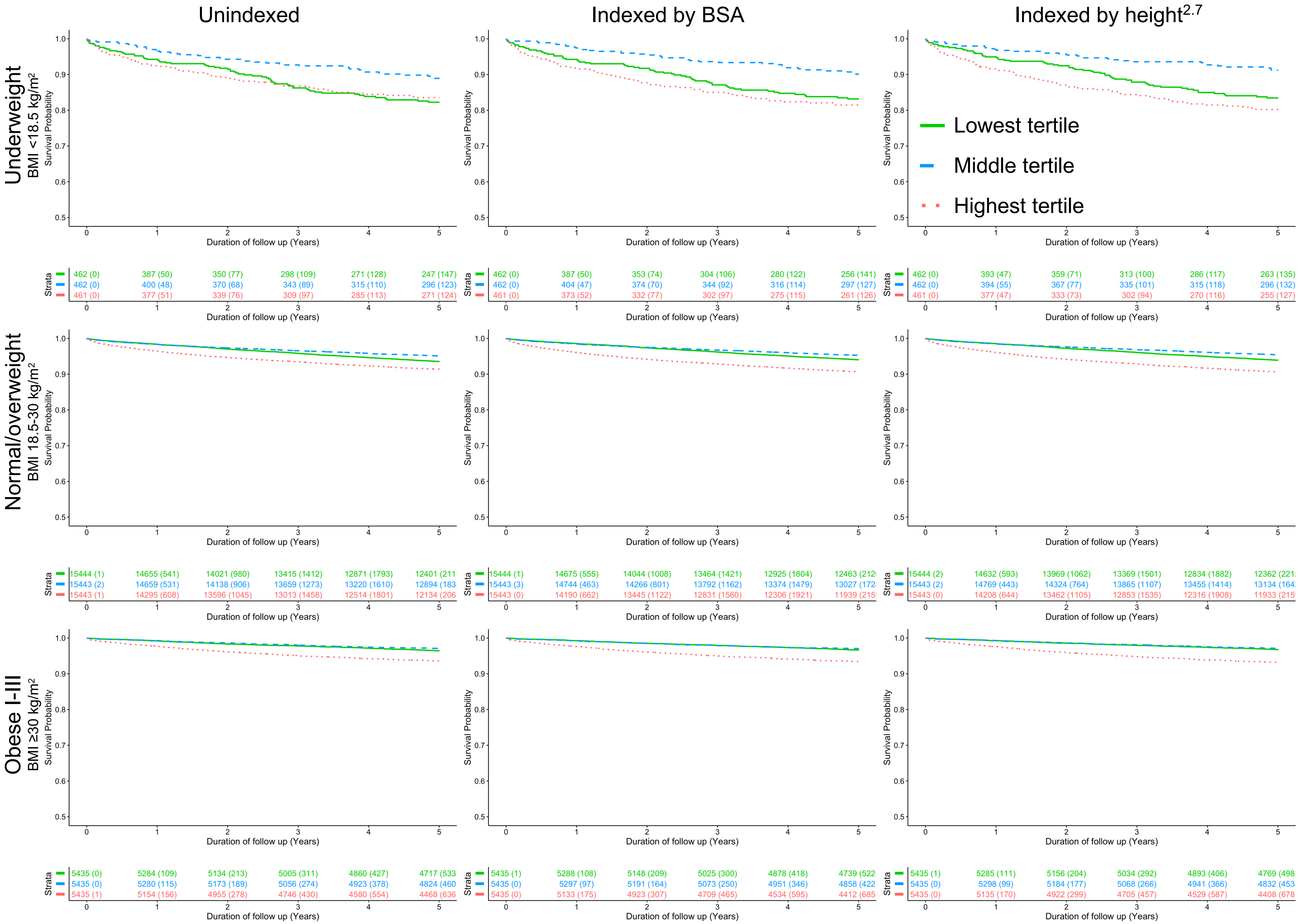

Supplementary Figure 25. Kaplan-Meir curves for 5-year cardiovascular mortality for LV end-diastolic volume unindexed, indexed by BSA and indexed by height$^{2.7}$.

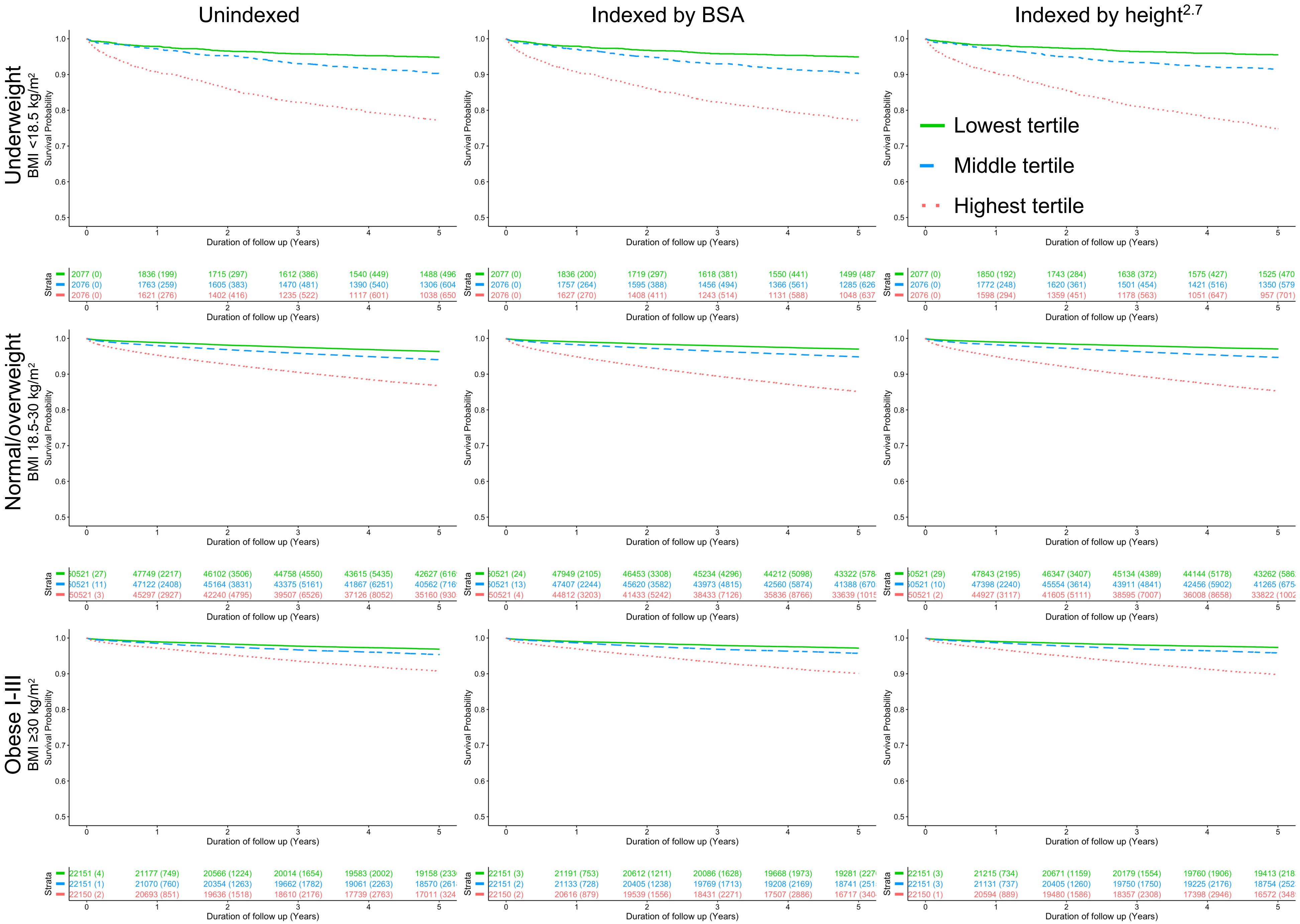

Supplementary Figure 26. Kaplan-Meir curves for 5-year cardiovascular mortality for LV mass unindexed, indexed by BSA and indexed by height$^{2.7}$.

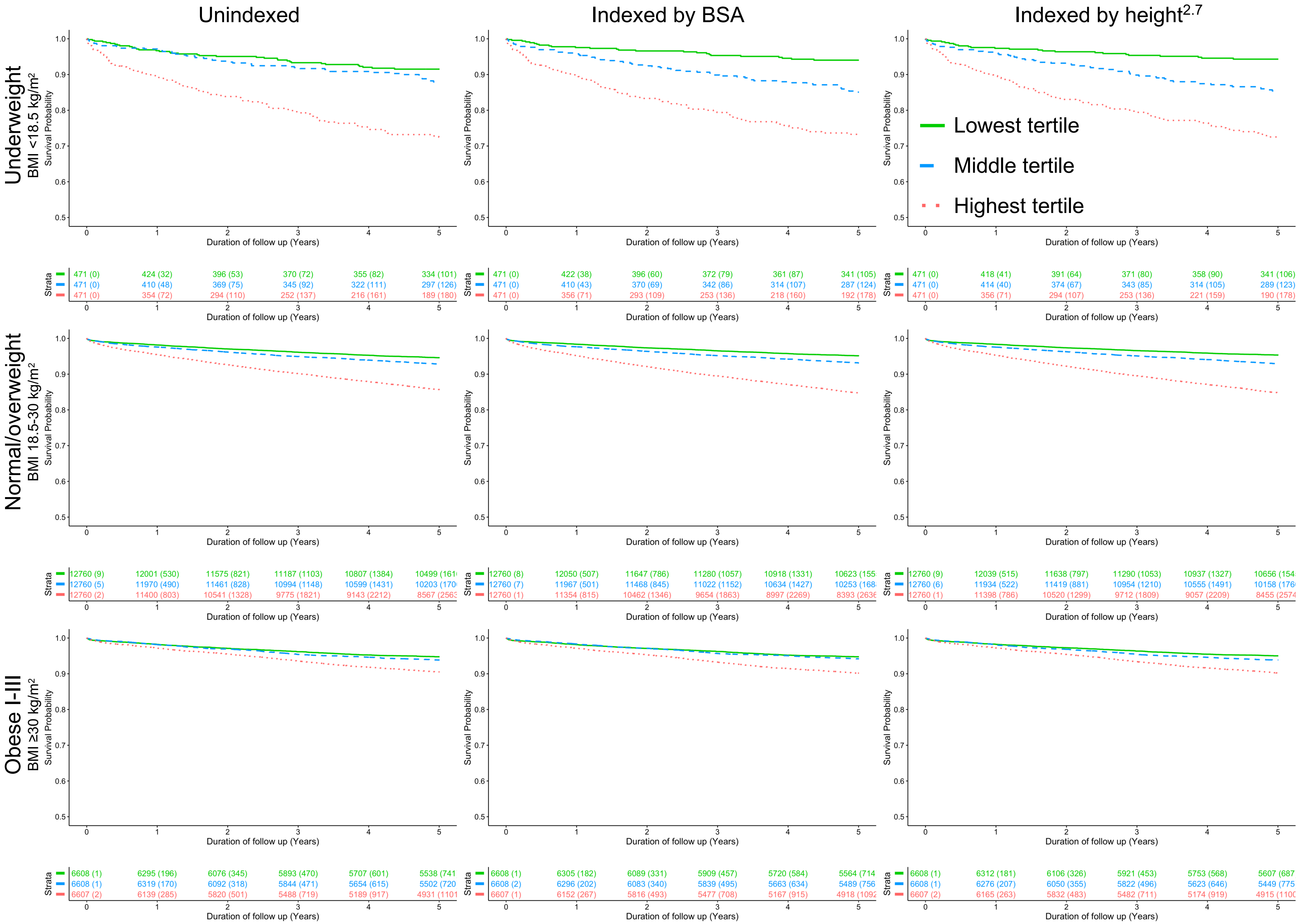

Supplementary Figure 27. Kaplan-Meir curves for 5-year cardiovascular mortality for RA area unindexed, indexed by BSA and indexed by height$^{2.7}$.

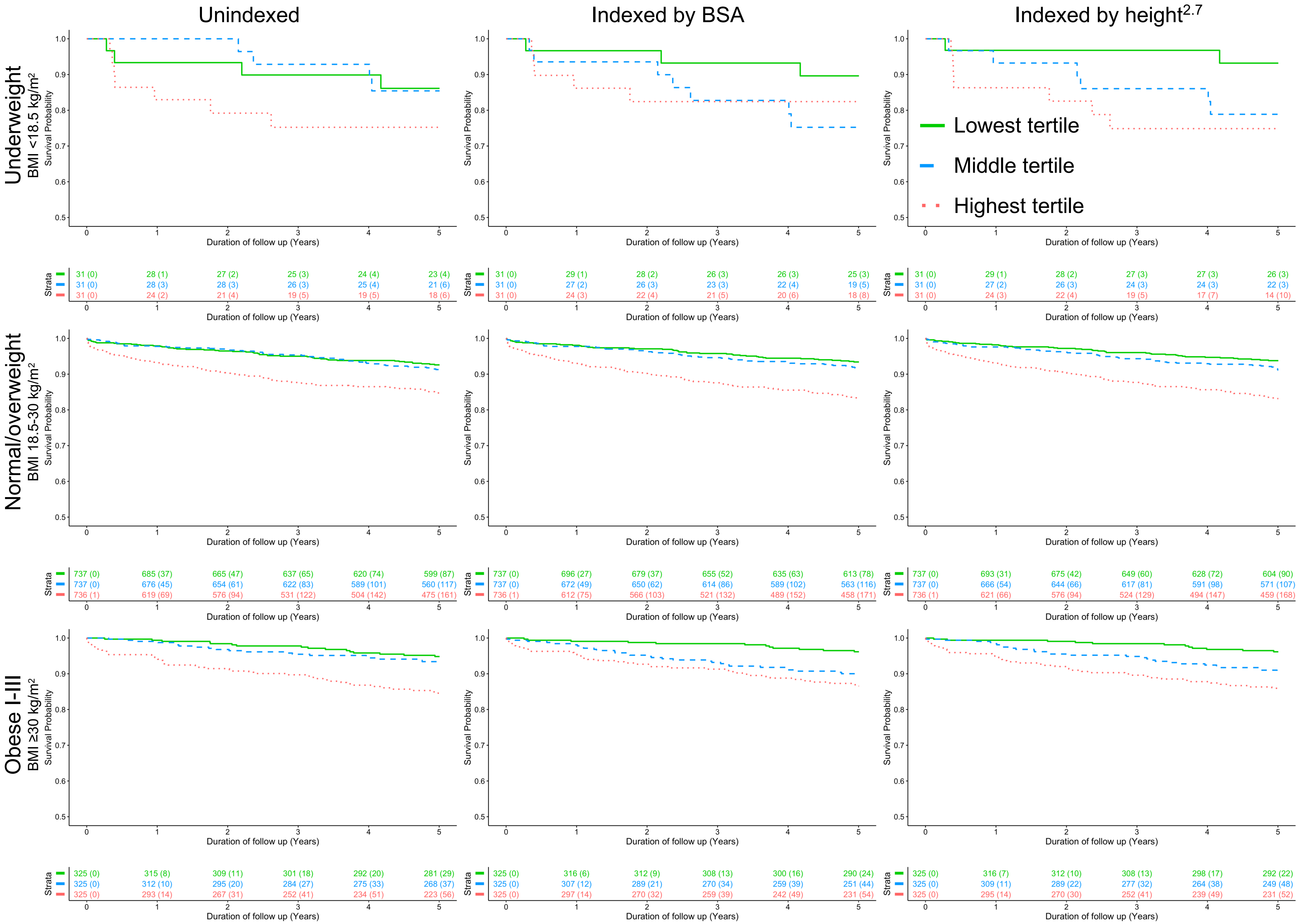

Supplementary Figure 28. Kaplan-Meir curves for 5-year cardiovascular mortality for RV diameter unindexed, indexed by BSA and indexed by height$^{2.7}$.
