# Supplementary material for: Prognostic association supports indexing size measures in echocardiography by body surface area
Source: Sci Rep. 2023 Nov 8;13:19390. doi: 10.1038/s41598-023-46183-z (PMC10632399; doi:10.1038/s41598-023-46183-z)
Supplement: Supplementary file 2 — Supplementary Tables. [file 41598_2023_46183_MOESM2_ESM.pdf]

**Supplementary table 1.** Subject characteristics for patients with available follow-up data for the respective cardiac or aortic measures. RA = right atrial, RV = right ventricular, RVOT = right ventricular outflow tract, LA = left atrial, LV = left ventricular, IVS = interventricular septum.

| | **RA area** | | | | | | |
|---|---|---|---|---|---|---|---|
| | **BMI <18.5 kg/m²** | **BMI 18.5-25 kg/m²** | **BMI 25-30 kg/m²** | **BMI 30-35 kg/m²** | **BMI 35-40 kg/m²** | **BMI ≥40 kg/m²** | **All** |
| **Subjects, n (%)** | 1413 (2) | 18200 (31) | 20080 (34) | 11973 (20) | 4784 (8) | 3066 (5) | 59516 (100) |
| **Events, n (%)** | 186 (13.2) | 1656 (9.1) | 1481 (7.4) | 777 (6.5) | 330 (6.9) | 183 (6.0) | 4613 (7.8) |
| **Female sex, n (%)** | 1012 (72) | 9714 (53) | 8250 (41) | 5367 (45) | 2535 (53) | 1865 (61) | 28743 (48) |
| **Age, years** | 68 [39-81] | 66 [47-78] | 66 [53-76] | 65 [54-74] | 63 [52-71] | 59 [49-68] | 65 [52-76] |
| **Follow-up, years** | 5.6 [2-8.2] | 7 [4.7-11.2] | 7.3 [5.3-11.3] | 7.2 [5.4-10.9] | 7.2 [5.4-10.3] | 6.9 [5.3-9.5] | 7.1 [5.2-11] |
| | **RV diameter** | | | | | | |
| | **BMI <18.5 kg/m²** | **BMI 18.5-25 kg/m²** | **BMI 25-30 kg/m²** | **BMI 30-35 kg/m²** | **BMI 35-40 kg/m²** | **BMI ≥40 kg/m²** | **All** |
| **Subjects, n (%)** | 93 (3) | 1062 (32) | 1148 (35) | 624 (19) | 226 (7) | 125 (4) | 3278 (100) |
| **Events, n (%)** | 15 (16.1) | 114 (10.7) | 97 (8.4) | 54 (8.7) | 20 (8.8) | 8 (6.4) | 308 (9.4) |
| **Female sex, n (%)** | 46 (50) | 416 (39) | 335 (29) | 220 (35) | 92 (41) | 62 (50) | 1171 (36) |
| **Age, years** | 60 [39-79] | 62 [43-76] | 63 [49-74] | 62 [49-71] | 59 [47-68] | 55 [46-65] | 62 [47-74] |
| **Follow-up, years** | 6.6 [3.2-9.4] | 6.8 [3.6-9.5] | 7 [5.4-9.6] | 7.1 [5.5-9.8] | 7.3 [5.5-9.7] | 7 [5.8-9.2] | 7 [5.1-9.6] |
| | **LA area** | | | | | | |
| | **BMI <18.5 kg/m²** | **BMI 18.5-25 kg/m²** | **BMI 25-30 kg/m²** | **BMI 30-35 kg/m²** | **BMI 35-40 kg/m²** | **BMI ≥40 kg/m²** | **All** |
| **Subjects, n (%)** | 2259 (2) | 28224 (31) | 30373 (34) | 17697 (20) | 7236 (8) | 4807 (5) | 90596 (100) |
| **Events, n (%)** | 302 (13.4) | 2634 (9.3) | 2267 (7.5) | 1182 (6.7) | 498 (6.9) | 316 (6.6) | 7199 (7.9) |
| **Female sex, n (%)** | 1573 (70) | 14468 (51) | 11922 (39) | 7678 (43) | 3692 (51) | 2899 (60) | 42232 (47) |
| **Age, years** | 67 [39-81] | 66 [47-78] | 66 [53-76] | 65 [53-74] | 63 [52-71] | 59 [49-68] | 65 [51-76] |
| **Follow-up, years** | 5.4 [1.7-9] | 7.1 [4.2-10.6] | 7.7 [5.2-10.9] | 7.7 [5.3-10.7] | 7.6 [5.3-10.5] | 7.3 [5.2-10] | 7.4 [5.1-10.6] |
| | **LA diameter** | | | | | | |
| | **BMI <18.5 kg/m²** | **BMI 18.5-25 kg/m²** | **BMI 25-30 kg/m²** | **BMI 30-35 kg/m²** | **BMI 35-40 kg/m²** | **BMI ≥40 kg/m²** | **All** |
| **Subjects, n (%)** | 2315 (3) | 29002 (32) | 30423 (34) | 16880 (19) | 6634 (7) | 4199 (5) | 89453 (100) |
| **Events, n (%)** | 227 (9.8) | 2254 (7.8) | 1883 (6.2) | 949 (5.6) | 382 (5.8) | 211 (5.0) | 5906 (6.6) |
| **Female sex, n (%)** | 1572 (68) | 15935 (55) | 12745 (42) | 8006 (47) | 3665 (55) | 2713 (65) | 44636 (50) |
| **Age, years** | 63 [35-78] | 62 [43-76] | 64 [51-74] | 63 [52-73] | 61 [50-70] | 58 [47-66] | 63 [49-74] |
| **Follow-up, years** | 6.6 [2.6-11] | 8.3 [5.2-12.8] | 8.5 [5.6-12.5] | 8.3 [5.7-12.1] | 8.1 [5.6-11.6] | 7.7 [5.5-11.1] | 8.3 [5.5-12.4] |

**Supplementary table 1.** Subject characteristics for patients with available follow-up data for the respective cardiac or aortic measures. RA = right atrial, RV = right ventricular, RVOT = right ventricular outflow tract, LA = left atrial, LV = left ventricular, IVS = interventricular septum.

| | **LA volume** | | | | | | |
|---|---|---|---|---|---|---|---|
| | **BMI <18.5 kg/m²** | **BMI 18.5-25 kg/m²** | **BMI 25-30 kg/m²** | **BMI 30-35 kg/m²** | **BMI 35-40 kg/m²** | **BMI ≥40 kg/m²** | **All** |
| **Subjects, n (%)** | 3314 (2) | 48955 (33) | 52965 (35) | 28382 (19) | 10633 (7) | 6291 (4) | 150540 (100) |
| **Events, n (%)** | 455 (13.7) | 3913 (8.0) | 3011 (5.7) | 1384 (4.9) | 519 (4.9) | 311 (4.9) | 9593 (6.4) |
| **Female sex, n (%)** | 2410 (73) | 26652 (54) | 21113 (40) | 12887 (45) | 5708 (54) | 3909 (62) | 72679 (48) |
| **Age, years** | 71 [45-83] | 66 [49-79] | 65 [53-76] | 64 [53-73] | 62 [51-71] | 58 [48-67] | 64 [51-76] |
| **Follow-up, years** | 5.7 [2-8.8] | 7.4 [5.1-10.2] | 7.8 [5.6-10.5] | 7.9 [5.7-10.5] | 7.8 [5.8-10.4] | 7.8 [5.7-10.2] | 7.7 [5.5-10.3] |
| | **LV end-diastolic diameter** | | | | | | |
| | **BMI <18.5 kg/m²** | **BMI 18.5-25 kg/m²** | **BMI 25-30 kg/m²** | **BMI 30-35 kg/m²** | **BMI 35-40 kg/m²** | **BMI ≥40 kg/m²** | **All** |
| **Subjects, n (%)** | 6375 (3) | 75797 (33) | 79414 (35) | 42274 (18) | 16191 (7) | 10058 (4) | 230109 (100) |
| **Events, n (%)** | 673 (10.6) | 6186 (8.2) | 4752 (6.0) | 2296 (5.4) | 849 (5.2) | 539 (5.4) | 15295 (6.6) |
| **Female sex, n (%)** | 4287 (67) | 40620 (54) | 31613 (40) | 19151 (45) | 8699 (54) | 6181 (62) | 110551 (48) |
| **Age, years** | 66 [42-80] | 64 [46-78] | 64 [52-75] | 63 [52-73] | 61 [50-70] | 58 [48-67] | 63 [50-75] |
| **Follow-up, years** | 6.1 [2.1-9.8] | 7.2 [5-10.5] | 7.5 [5.4-10.6] | 7.5 [5.5-10.5] | 7.4 [5.5-10.3] | 7.1 [5.4-9.9] | 7.3 [5.3-10.5] |
| | **LV end-diastolic volume** | | | | | | |
| | **BMI <18.5 kg/m²** | **BMI 18.5-25 kg/m²** | **BMI 25-30 kg/m²** | **BMI 30-35 kg/m²** | **BMI 35-40 kg/m²** | **BMI ≥40 kg/m²** | **All** |
| **Subjects, n (%)** | 1385 (2) | 22609 (35) | 23721 (37) | 11135 (17) | 3633 (6) | 1537 (2) | 64020 (100) |
| **Events, n (%)** | 177 (12.8) | 1707 (7.6) | 1182 (5.0) | 475 (4.3) | 135 (3.7) | 58 (3.8) | 3734 (5.8) |
| **Female sex, n (%)** | 1037 (75) | 12430 (55) | 9261 (39) | 5161 (46) | 2059 (57) | 1048 (68) | 30996 (48) |
| **Age, years** | 73 [47-84] | 66 [48-79] | 64 [52-75] | 63 [52-73] | 60 [50-70] | 59 [48-68] | 64 [50-76] |
| **Follow-up, years** | 5.6 [2.1-8.2] | 6.9 [5.2-9.2] | 7.2 [5.5-9.4] | 7.2 [5.6-9.4] | 7.2 [5.7-9.4] | 7 [5.5-8.9] | 7 [5.4-9.3] |
| | **IVS thickness** | | | | | | |
| | **BMI <18.5 kg/m²** | **BMI 18.5-25 kg/m²** | **BMI 25-30 kg/m²** | **BMI 30-35 kg/m²** | **BMI 35-40 kg/m²** | **BMI ≥40 kg/m²** | **All** |
| **Subjects, n (%)** | 6468 (3) | 76878 (33) | 80629 (35) | 42795 (18) | 16345 (7) | 10130 (4) | 233245 (100) |
| **Events, n (%)** | 677 (10.5) | 6305 (8.2) | 4855 (6.0) | 2331 (5.4) | 852 (5.2) | 539 (5.3) | 15559 (6.7) |
| **Female sex, n (%)** | 4361 (67) | 41092 (54) | 32034 (40) | 19358 (45) | 8796 (54) | 6244 (62) | 111885 (48) |
| **Age, years** | 66 [42-80] | 64 [46-78] | 64 [52-75] | 63 [52-73] | 61 [50-70] | 58 [47-67] | 63 [50-75] |
| **Follow-up, years** | 6.1 [2.1-9.8] | 7.1 [5-10.5] | 7.4 [5.4-10.6] | 7.5 [5.5-10.5] | 7.4 [5.5-10.2] | 7.1 [5.4-9.8] | 7.3 [5.3-10.4] |

**Supplementary table 1.** Subject characteristics for patients with available follow-up data for the respective cardiac or aortic measures. RA = right atrial, RV = right ventricular, RVOT = right ventricular outflow tract, LA = left atrial, LV = left ventricular, IVS = interventricular septum.

| | **LV mass** | | | | | | |
|---|---|---|---|---|---|---|---|
| | **BMI <18.5 kg/m$^2$** | **BMI 18.5-25 kg/m$^2$** | **BMI 25-30 kg/m$^2$** | **BMI 30-35 kg/m$^2$** | **BMI 35-40 kg/m$^2$** | **BMI ≥40 kg/m$^2$** | **All** |
| **Subjects, n (%)** | 6229 (3) | 74029 (33) | 77534 (35) | 41112 (18) | 15681 (7) | 9659 (4) | 224244 (100) |
| **Events, n (%)** | 650 (10.4) | 6000 (8.1) | 4583 (5.9) | 2204 (5.4) | 805 (5.1) | 512 (5.3) | 14754 (6.6) |
| **Female sex, n (%)** | 4205 (68) | 39815 (54) | 30997 (40) | 18715 (46) | 8506 (54) | 5989 (62) | 108227 (48) |
| **Age, years** | 66 [42-80] | 64 [46-78] | 64 [52-75] | 63 [52-73] | 61 [50-70] | 58 [48-67] | 63 [50-75] |
| **Follow-up, years** | 6.1 [2.1-9.9] | 7.1 [5-10.5] | 7.5 [5.4-10.6] | 7.5 [5.5-10.5] | 7.4 [5.5-10.3] | 7.1 [5.4-9.8] | 7.3 [5.3-10.5] |
| | **Aortic sinus diameter** | | | | | | |
| | **BMI <18.5 kg/m$^2$** | **BMI 18.5-25 kg/m$^2$** | **BMI 25-30 kg/m$^2$** | **BMI 30-35 kg/m$^2$** | **BMI 35-40 kg/m$^2$** | **BMI ≥40 kg/m$^2$** | **All** |
| **Subjects, n (%)** | 2157 (2) | 30172 (33) | 32361 (36) | 16629 (18) | 6032 (7) | 3454 (4) | 90805 (100) |
| **Events, n (%)** | 284 (13.2) | 2470 (8.2) | 1767 (5.5) | 793 (4.8) | 277 (4.6) | 174 (5.0) | 5765 (6.3) |
| **Female sex, n (%)** | 1551 (72) | 16252 (54) | 12411 (38) | 7364 (44) | 3181 (53) | 2054 (60) | 42813 (47) |
| **Age, years** | 72 [45-83] | 66 [49-80] | 65 [53-76] | 64 [53-73] | 61 [51-70] | 58 [49-67] | 64 [51-76] |
| **Follow-up, years** | 5.7 [1.8-8.4] | 7.3 [5.1-9.6] | 7.6 [5.6-9.8] | 7.6 [5.7-9.7] | 7.7 [5.8-9.6] | 7.4 [5.6-9.3] | 7.5 [5.4-9.7] |
| | **Aorta at sinotubular diameter** | | | | | | |
| | **BMI <18.5 kg/m$^2$** | **BMI 18.5-25 kg/m$^2$** | **BMI 25-30 kg/m$^2$** | **BMI 30-35 kg/m$^2$** | **BMI 35-40 kg/m$^2$** | **BMI ≥40 kg/m$^2$** | **All** |
| **Subjects, n (%)** | 1334 (2) | 18454 (30) | 21422 (35) | 12233 (20) | 4563 (8) | 2607 (4) | 60613 (100) |
| **Events, n (%)** | 199 (14.9) | 1757 (9.5) | 1432 (6.7) | 711 (5.8) | 274 (6.0) | 143 (5.5) | 4516 (7.5) |
| **Female sex, n (%)** | 922 (69) | 9072 (49) | 7552 (35) | 4963 (41) | 2219 (49) | 1491 (57) | 26219 (43) |
| **Age, years** | 72 [45-83] | 69 [52-80] | 67 [56-77] | 66 [55-75] | 63 [53-72] | 60 [50-68] | 67 [54-77] |
| **Follow-up, years** | 5.5 [1.6-8.6] | 7.4 [4.7-10.4] | 7.9 [5.5-10.8] | 7.9 [5.7-10.6] | 7.8 [5.7-10.4] | 7.7 [5.6-10.1] | 7.7 [5.3-10.5] |
| | **Aortic root diameter** | | | | | | |
| | **BMI <18.5 kg/m$^2$** | **BMI 18.5-25 kg/m$^2$** | **BMI 25-30 kg/m$^2$** | **BMI 30-35 kg/m$^2$** | **BMI 35-40 kg/m$^2$** | **BMI ≥40 kg/m$^2$** | **All** |
| **Subjects, n (%)** | 4773 (2) | 64890 (33) | 68263 (35) | 35731 (18) | 13498 (7) | 7983 (4) | 195138 (100) |
| **Events, n (%)** | 541 (11.3) | 5104 (7.9) | 3867 (5.7) | 1788 (5.0) | 673 (5.0) | 383 (4.8) | 12356 (6.3) |
| **Female sex, n (%)** | 3272 (69) | 35236 (54) | 27404 (40) | 16425 (46) | 7352 (55) | 5029 (63) | 94718 (49) |
| **Age, years** | 68 [41-81] | 64 [46-78] | 64 [52-75] | 63 [52-73] | 61 [51-70] | 58 [48-67] | 63 [50-75] |
| **Follow-up, years** | 5.9 [2.1-9.6] | 7.4 [5.1-10.9] | 7.7 [5.6-11] | 7.8 [5.7-10.9] | 7.7 [5.7-10.7] | 7.4 [5.5-10.3] | 7.6 [5.4-10.9] |

**Supplementary table 1.** Subject characteristics for patients with available follow-up data for the respective cardiac or aortic measures. RA = right atrial, RV = right ventricular, RVOT = right ventricular outflow tract, LA = left atrial, LV = left ventricular, IVS = interventricular septum.

| | Ascending aorta diameter | | | | | | |
|---|---|---|---|---|---|---|---|
| | **BMI <18.5 kg/m²** | **BMI 18.5-25 kg/m²** | **BMI 25-30 kg/m²** | **BMI 30-35 kg/m²** | **BMI 35-40 kg/m²** | **BMI ≥40 kg/m²** | **All** |
| **Subjects, n (%)** | 2693 (3) | 30316 (30) | 34761 (34) | 20283 (20) | 8117 (8) | 5219 (5) | 101389 (100) |
| **Events, n (%)** | 295 (11.0) | 2977 (9.8) | 2610 (7.5) | 1355 (6.7) | 539 (6.6) | 358 (6.9) | 8134 (8.0) |
| **Female sex, n (%)** | 1770 (66) | 15311 (51) | 13455 (39) | 8743 (43) | 4033 (50) | 3101 (59) | 46413 (46) |
| **Age, years** | 66 [43-80] | 67 [49-79] | 66 [54-77] | 65 [54-74] | 62 [52-71] | 59 [49-68] | 65 [52-76] |
| **Follow-up, years** | 5.9 [1.9-9.6] | 6.5 [3.6-9.3] | 6.9 [5.1-9.8] | 7 [5.2-9.7] | 6.9 [5.2-9.6] | 6.7 [5.2-9.2] | 6.8 [4.9-9.6] |
| | **Aortic arch diameter** | | | | | | |
| | **BMI <18.5 kg/m²** | **BMI 18.5-25 kg/m²** | **BMI 25-30 kg/m²** | **BMI 30-35 kg/m²** | **BMI 35-40 kg/m²** | **BMI ≥40 kg/m²** | **All** |
| **Subjects, n (%)** | 454 (3) | 5127 (30) | 5861 (35) | 3324 (20) | 1318 (8) | 763 (5) | 16847 (100) |
| **Events, n (%)** | 43 (9.5) | 440 (8.6) | 363 (6.2) | 183 (5.5) | 77 (5.8) | 52 (6.8) | 1158 (6.9) |
| **Female sex, n (%)** | 303 (67) | 2532 (49) | 2135 (36) | 1303 (39) | 638 (48) | 452 (59) | 7363 (44) |
| **Age, years** | 52 [26-77] | 63 [42-77] | 64 [51-75] | 62 [50-72] | 61 [50-69] | 57 [47-66] | 63 [48-74] |
| **Follow-up, years** | 5.8 [2.1-8.7] | 6.6 [4.8-9] | 6.7 [5.3-9.1] | 6.9 [5.4-9.1] | 6.7 [5.3-8.9] | 6.6 [5.2-8.5] | 6.7 [5.2-9] |

**Supplementary Table 2.** Statistical analyses for 5-year cardiovascular mortality for aorta at sinotubular diameter indexed by different metrics.

BSA = body surface area, [M] = Mosteller, [DB] = Du Bois, LBM = lean body mass, [H] = Hume, [B] = Boer, [J] = James, BMI = body mass index.

| | C statistic | | | Hazard Ratio per 1 SD increment | | | p value | | |
|---|---|---|---|---|---|---|---|---|---|
| | **BMI <18.5 kg/m²** | **BMI 18.5-30 kg/m²** | **BMI ≥30 kg/m²** | **BMI <18.5 kg/m²** | **BMI 18.5-30 kg/m²** | **BMI ≥30 kg/m²** | **BMI <18.5 kg/m²** | **BMI 18.5-30 kg/m²** | **BMI ≥30 kg/m²** |
| **Weight** | 0.573 [0.532-0.613] | 0.554 [0.544-0.565] | 0.498 [0.480-0.516] | 1.23 [1.11-1.35] | 1.28 [1.23-1.32] | 1.02 [0.94-1.11] | <0.0001 | <0.0001 | 0.63 |
| **Weight^1.5** | 0.589 [0.549-0.628] | 0.570 [0.560-0.580] | 0.508 [0.491-0.526] | 1.21 [1.11-1.32] | 1.33 [1.29-1.38] | 1.10 [0.98-1.22] | <0.0001 | <0.0001 | 0.10 |
| **Height*Weight** | 0.585 [0.546-0.625] | 0.562 [0.551-0.572] | 0.508 [0.491-0.526] | 1.24 [1.12-1.36] | 1.28 [1.24-1.33] | 1.07 [0.99-1.17] | <0.0001 | <0.0001 | 0.10 |
| **BSA[M]^1.5** | 0.574 [0.534-0.614] | 0.550 [0.539-0.560] | 0.500 [0.482-0.518] | 1.23 [1.12-1.37] | 1.24 [1.20-1.29] | 1.03 [0.96-1.11] | <0.0001 | <0.0001 | 0.44 |
| **BSA[DB]^1.5** | 0.574 [0.534-0.614] | 0.547 [0.537-0.558] | 0.501 [0.484-0.519] | 1.24 [1.12-1.37] | 1.23 [1.19-1.27] | 1.03 [0.96-1.11] | <0.0001 | <0.0001 | 0.37 |
| **BSA[M]** | 0.558 [0.517-0.598] | 0.532 [0.522-0.543] | 0.509 [0.490-0.527] | 1.21 [1.09-1.35] | 1.17 [1.13-1.21] | 0.98 [0.92-1.05] | 0.0004 | <0.0001 | 0.66 |
| **BSA[DB]** | 0.558 [0.517-0.599] | 0.530 [0.520-0.541] | 0.508 [0.489-0.526] | 1.22 [1.09-1.36] | 1.16 [1.12-1.20] | 0.99 [0.93-1.06] | 0.0004 | <0.0001 | 0.73 |
| **LBM[H]** | 0.569 [0.529-0.609] | 0.535 [0.525-0.545] | 0.495 [0.478-0.513] | 1.25 [1.12-1.39] | 1.17 [1.14-1.21] | 1.00 [0.94-1.07] | <0.0001 | <0.0001 | 0.89 |
| **LBM[B]** | 0.569 [0.529-0.609] | 0.532 [0.521-0.542] | 0.511 [0.493-0.528] | 1.27 [1.13-1.42] | 1.16 [1.12-1.20] | 0.98 [0.92-1.04] | <0.0001 | <0.0001 | 0.54 |
| **LBM[J]** | 0.566 [0.526-0.606] | 0.533 [0.523-0.543] | 0.487 [0.469-0.504] | 1.24 [1.11-1.39] | 1.18 [1.13-1.22] | 1.00 [0.95-1.06] | 0.0001 | <0.0001 | 0.89 |
| **BMI** | 0.526 [0.484-0.568] | 0.525 [0.514-0.536] | 0.525 [0.507-0.543] | 1.13 [1.01-1.26] | 1.15 [1.11-1.20] | 0.90 [0.82-0.98] | 0.028 | <0.0001 | 0.016 |
| **Height^2.7** | 0.569 [0.530-0.609] | 0.528 [0.518-0.538] | 0.504 [0.486-0.522] | 1.29 [1.14-1.47] | 1.15 [1.11-1.19] | 1.03 [0.98-1.09] | <0.0001 | <0.0001 | 0.25 |
| **Height^2.13** | 0.561 [0.521-0.601] | 0.520 [0.510-0.531] | 0.498 [0.480-0.516] | 1.27 [1.12-1.45] | 1.12 [1.09-1.17] | 1.01 [0.96-1.07] | 0.0002 | <0.0001 | 0.69 |
| **Height^2** | 0.560 [0.520-0.600] | 0.518 [0.508-0.529] | 0.497 [0.479-0.514] | 1.27 [1.11-1.44] | 1.12 [1.08-1.16] | 1.01 [0.95-1.07] | 0.0003 | <0.0001 | 0.84 |
| **Height^1.5** | 0.551 [0.510-0.591] | 0.511 [0.500-0.522] | 0.508 [0.490-0.526] | 1.24 [1.09-1.41] | 1.08 [1.05-1.12] | 0.98 [0.93-1.04] | 0.0012 | <0.0001 | 0.54 |
| **Height** | 0.539 [0.498-0.581] | 0.503 [0.492-0.514] | 0.513 [0.495-0.531] | 1.21 [1.06-1.37] | 1.05 [1.01-1.08] | 0.96 [0.90-1.01] | 0.0055 | 0.012 | 0.13 |
| ***Unindexed*** | *0.514 [0.472-0.556]* | *0.514 [0.503-0.525]* | *0.526 [0.507-0.544]* | *1.12 [0.97-1.28]* | *0.97 [0.93-1.00]* | *0.91 [0.86-0.96]* | *0.12* | *0.077* | *0.0012* |

**Supplementary Table 3.** Statistical analyses for 5-year cardiovascular mortality for aortic sinus diameter indexed by different metrics.

BSA = body surface area, [M] = Mosteller, [DB] = Du Bois, LBM = lean body mass, [H] = Hume, [B] = Boer, [J] = James, BMI = body mass index.

| | **C statistic** | | | **Hazard Ratio per 1 SD increment** | | | **p value** | | |
|---|---|---|---|---|---|---|---|---|---|
| | **BMI <18.5 kg/m$^2$** | **BMI 18.5-30 kg/m$^2$** | **BMI ≥30 kg/m$^2$** | **BMI <18.5 kg/m$^2$** | **BMI 18.5-30 kg/m$^2$** | **BMI ≥30 kg/m$^2$** | **BMI <18.5 kg/m$^2$** | **BMI 18.5-30 kg/m$^2$** | **BMI ≥30 kg/m$^2$** |
| **Weight** | 0.640 [0.610-0.670] | 0.645 [0.636-0.653] | 0.562 [0.546-0.578] | 1.36 [1.26-1.47] | 1.76 [1.70-1.81] | 1.47 [1.34-1.61] | <0.0001 | <0.0001 | <0.0001 |
| **Weight^1.5** | 0.629 [0.598-0.659] | 0.634 [0.625-0.642] | 0.553 [0.537-0.570] | 1.26 [1.18-1.34] | 1.69 [1.64-1.74] | 1.54 [1.38-1.72] | <0.0001 | <0.0001 | <0.0001 |
| **Height*Weight** | 0.627 [0.597-0.658] | 0.637 [0.629-0.645] | 0.564 [0.547-0.580] | 1.32 [1.22-1.42] | 1.64 [1.60-1.69] | 1.45 [1.34-1.57] | <0.0001 | <0.0001 | <0.0001 |
| **BSA[M]^1.5** | 0.635 [0.605-0.665] | 0.645 [0.637-0.653] | 0.571 [0.554-0.587] | 1.38 [1.27-1.49] | 1.68 [1.63-1.73] | 1.44 [1.34-1.56] | <0.0001 | <0.0001 | <0.0001 |
| **BSA[DB]^1.5** | 0.633 [0.603-0.663] | 0.644 [0.635-0.652] | 0.574 [0.557-0.590] | 1.37 [1.27-1.49] | 1.64 [1.59-1.68] | 1.42 [1.33-1.52] | <0.0001 | <0.0001 | <0.0001 |
| **BSA[M]** | 0.642 [0.613-0.672] | 0.646 [0.638-0.654] | 0.576 [0.560-0.593] | 1.40 [1.29-1.53] | 1.63 [1.59-1.68] | 1.39 [1.30-1.48] | <0.0001 | <0.0001 | <0.0001 |
| **BSA[DB]** | 0.641 [0.611-0.671] | 0.645 [0.637-0.653] | 0.579 [0.563-0.596] | 1.40 [1.29-1.53] | 1.60 [1.56-1.64] | 1.38 [1.30-1.47] | <0.0001 | <0.0001 | <0.0001 |
| **LBM[H]** | 0.624 [0.594-0.654] | 0.628 [0.620-0.636] | 0.564 [0.548-0.580] | 1.36 [1.25-1.48] | 1.52 [1.48-1.56] | 1.32 [1.24-1.40] | <0.0001 | <0.0001 | <0.0001 |
| **LBM[B]** | 0.626 [0.596-0.656] | 0.617 [0.608-0.625] | 0.543 [0.527-0.559] | 1.39 [1.27-1.52] | 1.48 [1.44-1.52] | 1.22 [1.15-1.29] | <0.0001 | <0.0001 | <0.0001 |
| **LBM[J]** | 0.626 [0.595-0.657] | 0.619 [0.610-0.627] | 0.546 [0.530-0.562] | 1.38 [1.26-1.50] | 1.57 [1.52-1.62] | 1.07 [1.05-1.08] | <0.0001 | <0.0001 | <0.0001 |
| **BMI** | 0.624 [0.592-0.655] | 0.615 [0.607-0.624] | 0.533 [0.517-0.549] | 1.28 [1.18-1.37] | 1.61 [1.56-1.67] | 1.20 [1.10-1.31] | <0.0001 | <0.0001 | <0.0001 |
| **Height^2.7** | 0.620 [0.590-0.650] | 0.618 [0.610-0.626] | 0.580 [0.564-0.596] | 1.42 [1.29-1.57] | 1.46 [1.42-1.50] | 1.27 [1.22-1.32] | <0.0001 | <0.0001 | <0.0001 |
| **Height^2.13** | 0.628 [0.598-0.658] | 0.621 [0.613-0.629] | 0.584 [0.568-0.601] | 1.44 [1.31-1.59] | 1.47 [1.43-1.51] | 1.32 [1.26-1.37] | <0.0001 | <0.0001 | <0.0001 |
| **Height^2** | 0.630 [0.600-0.659] | 0.621 [0.613-0.630] | 0.585 [0.569-0.601] | 1.44 [1.31-1.59] | 1.47 [1.43-1.51] | 1.32 [1.27-1.39] | <0.0001 | <0.0001 | <0.0001 |
| **Height^1.5** | 0.635 [0.605-0.664] | 0.618 [0.610-0.626] | 0.585 [0.569-0.601] | 1.45 [1.32-1.60] | 1.45 [1.41-1.49] | 1.34 [1.28-1.41] | <0.0001 | <0.0001 | <0.0001 |
| **Height** | 0.635 [0.606-0.665] | 0.607 [0.599-0.616] | 0.579 [0.563-0.596] | 1.44 [1.31-1.59] | 1.40 [1.37-1.44] | 1.32 [1.25-1.39] | <0.0001 | <0.0001 | <0.0001 |
| ***Unindexed*** | *0.613 [0.582-0.644]* | *0.568 [0.560-0.577]* | *0.552 [0.536-0.568]* | *1.36 [1.22-1.51]* | *1.26 [1.22-1.29]* | *1.20 [1.14-1.26]* | *<0.0001* | *<0.0001* | *<0.0001* |

**Supplementary Table 4.** Statistical analyses for 5-year cardiovascular mortality for aortic arch diameter indexed by different metrics.

BSA = body surface area, [M] = Mosteller, [DB] = Du Bois, LBM = lean body mass, [H] = Hume, [B] = Boer, [J] = James, BMI = body mass index.

| | C statistic | | | Hazard Ratio per 1 SD increment | | | p value | | |
|---|---|---|---|---|---|---|---|---|---|
| | BMI <18.5 kg/m$^2$ | BMI 18.5-30 kg/m$^2$ | BMI ≥30 kg/m$^2$ | BMI <18.5 kg/m$^2$ | BMI 18.5-30 kg/m$^2$ | BMI ≥30 kg/m$^2$ | BMI <18.5 kg/m$^2$ | BMI 18.5-30 kg/m$^2$ | BMI ≥30 kg/m$^2$ |
| **Weight** | 0.685 [0.613-0.757] | 0.620 [0.601-0.640] | 0.516 [0.483-0.549] | 1.29 [1.12-1.49] | 1.56 [1.46-1.66] | 1.19 [1.01-1.40] | 0.0006 | <0.0001 | 0.037 |
| **Weight^1.5** | 0.679 [0.607-0.750] | 0.625 [0.605-0.644] | 0.521 [0.488-0.554] | 1.21 [1.07-1.37] | 1.59 [1.49-1.69] | 1.26 [1.02-1.54] | 0.0021 | <0.0001 | 0.030 |
| **Height*Weight** | 0.679 [0.608-0.749] | 0.627 [0.608-0.646] | 0.527 [0.494-0.561] | 1.25 [1.09-1.44] | 1.54 [1.45-1.63] | 1.27 [1.09-1.48] | 0.0011 | <0.0001 | 0.0024 |
| **BSA[M]^1.5** | 0.682 [0.611-0.754] | 0.622 [0.602-0.641] | 0.523 [0.490-0.556] | 1.30 [1.12-1.51] | 1.52 [1.43-1.61] | 1.23 [1.07-1.41] | 0.0005 | <0.0001 | 0.0042 |
| **BSA[DB]^1.5** | 0.682 [0.611-0.753] | 0.622 [0.602-0.641] | 0.526 [0.493-0.559] | 1.31 [1.12-1.52] | 1.50 [1.41-1.59] | 1.24 [1.09-1.41] | 0.0005 | <0.0001 | 0.0012 |
| **BSA[M]** | 0.678 [0.605-0.751] | 0.608 [0.588-0.628] | 0.516 [0.482-0.549] | 1.35 [1.15-1.60] | 1.46 [1.37-1.55] | 1.18 [1.04-1.34] | 0.0003 | <0.0001 | 0.011 |
| **BSA[DB]** | 0.677 [0.604-0.750] | 0.608 [0.588-0.627] | 0.518 [0.485-0.552] | 1.36 [1.15-1.61] | 1.45 [1.37-1.54] | 1.19 [1.05-1.35] | 0.0003 | <0.0001 | 0.0049 |
| **LBM[H]** | 0.676 [0.603-0.749] | 0.612 [0.592-0.631] | 0.523 [0.490-0.557] | 1.32 [1.12-1.54] | 1.44 [1.36-1.52] | 1.22 [1.08-1.38] | 0.0007 | <0.0001 | 0.001 |
| **LBM[B]** | 0.680 [0.607-0.752] | 0.606 [0.586-0.626] | 0.521 [0.488-0.555] | 1.34 [1.13-1.59] | 1.42 [1.34-1.50] | 1.20 [1.07-1.34] | 0.0006 | <0.0001 | 0.0019 |
| **LBM[J]** | 0.676 [0.603-0.749] | 0.606 [0.586-0.626] | 0.531 [0.497-0.564] | 1.37 [1.14-1.64] | 1.53 [1.43-1.64] | 1.09 [1.06-1.11] | 0.0006 | <0.0001 | <0.0001 |
| **BMI** | 0.641 [0.558-0.723] | 0.575 [0.555-0.596] | 0.517 [0.484-0.550] | 1.33 [1.12-1.58] | 1.43 [1.33-1.53] | 0.98 [0.83-1.15] | 0.001 | <0.0001 | 0.77 |
| **Height^2.7** | 0.671 [0.598-0.743] | 0.608 [0.589-0.628] | 0.538 [0.505-0.571] | 1.42 [1.16-1.74] | 1.44 [1.35-1.53] | 1.26 [1.15-1.38] | 0.0008 | <0.0001 | <0.0001 |
| **Height^2.13** | 0.670 [0.598-0.743] | 0.602 [0.583-0.622] | 0.532 [0.499-0.566] | 1.44 [1.17-1.76] | 1.42 [1.34-1.51] | 1.26 [1.14-1.39] | 0.0005 | <0.0001 | <0.0001 |
| **Height^2** | 0.671 [0.598-0.744] | 0.601 [0.581-0.620] | 0.531 [0.498-0.564] | 1.44 [1.17-1.77] | 1.42 [1.33-1.51] | 1.26 [1.13-1.39] | 0.0005 | <0.0001 | <0.0001 |
| **Height^1.5** | 0.669 [0.595-0.743] | 0.591 [0.572-0.611] | 0.525 [0.491-0.558] | 1.47 [1.19-1.81] | 1.39 [1.31-1.48] | 1.23 [1.10-1.37] | 0.0004 | <0.0001 | 0.0002 |
| **Height** | 0.661 [0.585-0.737] | 0.579 [0.559-0.599] | 0.517 [0.484-0.550] | 1.49 [1.20-1.86] | 1.35 [1.27-1.44] | 1.18 [1.06-1.32] | 0.0003 | <0.0001 | 0.0032 |
| ***Unindexed*** | *0.630 [0.549-0.712]* | *0.548 [0.528-0.569]* | *0.502 [0.469-0.535]* | *1.55 [1.20-2.00]* | *1.24 [1.16-1.32]* | *1.07 [0.96-1.20]* | *0.0007* | *<0.0001* | *0.22* |

**Supplementary Table 5.** Statistical analyses for 5-year cardiovascular mortality for aortic root diameter indexed by different metrics.

BSA = body surface area, [M] = Mosteller, [DB] = Du Bois, LBM = lean body mass, [H] = Hume, [B] = Boer, [J] = James, BMI = body mass index.

| | **C statistic** | | | **Hazard Ratio per 1 SD increment** | | | **p value** | | |
|---|---|---|---|---|---|---|---|---|---|
| | **BMI <18.5 kg/m$^2$** | **BMI 18.5-30 kg/m$^2$** | **BMI ≥30 kg/m$^2$** | **BMI <18.5 kg/m$^2$** | **BMI 18.5-30 kg/m$^2$** | **BMI ≥30 kg/m$^2$** | **BMI <18.5 kg/m$^2$** | **BMI 18.5-30 kg/m$^2$** | **BMI ≥30 kg/m$^2$** |
| **Weight** | 0.631 [0.609-0.653] | 0.626 [0.620-0.632] | 0.568 [0.557-0.579] | 1.23 [1.17-1.29] | 1.65 [1.61-1.68] | 1.50 [1.41-1.59] | <0.0001 | <0.0001 | <0.0001 |
| **Weight^1.5** | 0.623 [0.601-0.646] | 0.617 [0.611-0.623] | 0.562 [0.551-0.573] | 1.14 [1.10-1.19] | 1.60 [1.57-1.64] | 1.57 [1.47-1.67] | <0.0001 | <0.0001 | <0.0001 |
| **Height*Weight** | 0.626 [0.603-0.648] | 0.622 [0.616-0.627] | 0.570 [0.559-0.580] | 1.24 [1.18-1.30] | 1.56 [1.53-1.60] | 1.42 [1.36-1.48] | <0.0001 | <0.0001 | <0.0001 |
| **BSA[M]^1.5** | 0.632 [0.610-0.655] | 0.627 [0.622-0.633] | 0.574 [0.563-0.584] | 1.30 [1.23-1.37] | 1.59 [1.56-1.62] | 1.43 [1.37-1.50] | <0.0001 | <0.0001 | <0.0001 |
| **BSA[DB]^1.5** | 0.632 [0.610-0.655] | 0.627 [0.621-0.632] | 0.575 [0.565-0.586] | 1.33 [1.25-1.40] | 1.56 [1.53-1.59] | 1.39 [1.34-1.45] | <0.0001 | <0.0001 | <0.0001 |
| **BSA[M]** | 0.634 [0.612-0.656] | 0.626 [0.620-0.632] | 0.575 [0.564-0.586] | 1.35 [1.28-1.44] | 1.54 [1.51-1.57] | 1.38 [1.33-1.44] | <0.0001 | <0.0001 | <0.0001 |
| **BSA[DB]** | 0.635 [0.613-0.657] | 0.625 [0.620-0.631] | 0.577 [0.566-0.588] | 1.37 [1.29-1.46] | 1.52 [1.49-1.55] | 1.37 [1.32-1.43] | <0.0001 | <0.0001 | <0.0001 |
| **LBM[H]** | 0.626 [0.603-0.649] | 0.612 [0.606-0.617] | 0.565 [0.554-0.576] | 1.38 [1.29-1.46] | 1.43 [1.41-1.45] | 1.29 [1.24-1.33] | <0.0001 | <0.0001 | <0.0001 |
| **LBM[B]** | 0.627 [0.605-0.650] | 0.600 [0.594-0.606] | 0.546 [0.535-0.556] | 1.41 [1.32-1.50] | 1.39 [1.37-1.42] | 1.21 [1.16-1.25] | <0.0001 | <0.0001 | <0.0001 |
| **LBM[J]** | 0.617 [0.594-0.640] | 0.601 [0.595-0.607] | 0.544 [0.533-0.555] | 1.27 [1.20-1.34] | 1.47 [1.44-1.50] | 1.07 [1.05-1.08] | <0.0001 | <0.0001 | <0.0001 |
| **BMI** | 0.595 [0.572-0.618] | 0.596 [0.590-0.602] | 0.543 [0.532-0.554] | 1.15 [1.09-1.22] | 1.51 [1.47-1.54] | 1.27 [1.20-1.34] | <0.0001 | <0.0001 | <0.0001 |
| **Height^2.7** | 0.633 [0.611-0.656] | 0.608 [0.602-0.613] | 0.579 [0.568-0.589] | 1.50 [1.40-1.61] | 1.43 [1.41-1.46] | 1.25 [1.22-1.28] | <0.0001 | <0.0001 | <0.0001 |
| **Height^2.13** | 0.637 [0.614-0.660] | 0.609 [0.604-0.615] | 0.581 [0.570-0.591] | 1.51 [1.41-1.63] | 1.44 [1.41-1.46] | 1.29 [1.25-1.33] | <0.0001 | <0.0001 | <0.0001 |
| **Height^2** | 0.638 [0.615-0.660] | 0.609 [0.604-0.615] | 0.581 [0.570-0.591] | 1.52 [1.41-1.63] | 1.44 [1.41-1.46] | 1.30 [1.26-1.34] | <0.0001 | <0.0001 | <0.0001 |
| **Height^1.5** | 0.637 [0.615-0.659] | 0.606 [0.600-0.611] | 0.579 [0.568-0.589] | 1.51 [1.40-1.63] | 1.41 [1.39-1.44] | 1.31 [1.27-1.35] | <0.0001 | <0.0001 | <0.0001 |
| **Height** | 0.631 [0.608-0.654] | 0.597 [0.591-0.602] | 0.573 [0.562-0.584] | 1.49 [1.38-1.60] | 1.37 [1.35-1.40] | 1.30 [1.25-1.34] | <0.0001 | <0.0001 | <0.0001 |
| ***Unindexed*** | *0.599 [0.575-0.622]* | *0.566 [0.560-0.571]* | *0.551 [0.541-0.562]* | *1.36 [1.26-1.48]* | *1.25 [1.22-1.27]* | *1.20 [1.16-1.24]* | *<0.0001* | *<0.0001* | *<0.0001* |

**Supplementary Table 6.** Statistical analyses for 5-year cardiovascular mortality for ascending aorta diameter indexed by different metrics.

BSA = body surface area, [M] = Mosteller, [DB] = Du Bois, LBM = lean body mass, [H] = Hume, [B] = Boer, [J] = James, BMI = body mass index.

| | **C statistic** | | | **Hazard Ratio per 1 SD increment** | | | **p value** | | |
|---|---|---|---|---|---|---|---|---|---|
| | **BMI <18.5 kg/m²** | **BMI 18.5-30 kg/m²** | **BMI ≥30 kg/m²** | **BMI <18.5 kg/m²** | **BMI 18.5-30 kg/m²** | **BMI ≥30 kg/m²** | **BMI <18.5 kg/m²** | **BMI 18.5-30 kg/m²** | **BMI ≥30 kg/m²** |
| **Weight** | 0.559 [0.531-0.587] | 0.629 [0.622-0.637] | 0.583 [0.572-0.595] | 1.07 [1.00-1.15] | 1.64 [1.59-1.68] | 1.49 [1.40-1.57] | 0.044 | <0.0001 | <0.0001 |
| **Weight^1.5** | 0.555 [0.528-0.582] | 0.627 [0.619-0.634] | 0.578 [0.566-0.590] | 1.01 [0.96-1.07] | 1.64 [1.60-1.69] | 1.65 [1.54-1.77] | 0.57 | <0.0001 | <0.0001 |
| **Height*Weight** | 0.571 [0.543-0.598] | 0.627 [0.620-0.635] | 0.584 [0.572-0.595] | 1.10 [1.03-1.17] | 1.59 [1.55-1.63] | 1.49 [1.41-1.57] | 0.0061 | <0.0001 | <0.0001 |
| **BSA[M]^1.5** | 0.574 [0.545-0.602] | 0.629 [0.622-0.637] | 0.588 [0.576-0.599] | 1.16 [1.07-1.26] | 1.60 [1.56-1.64] | 1.44 [1.37-1.51] | 0.0004 | <0.0001 | <0.0001 |
| **BSA[DB]^1.5** | 0.582 [0.553-0.611] | 0.628 [0.621-0.636] | 0.589 [0.578-0.601] | 1.21 [1.11-1.33] | 1.59 [1.54-1.63] | 1.42 [1.35-1.48] | <0.0001 | <0.0001 | <0.0001 |
| **BSA[M]** | 0.579 [0.550-0.608] | 0.629 [0.621-0.636] | 0.591 [0.580-0.603] | 1.26 [1.14-1.40] | 1.59 [1.54-1.63] | 1.38 [1.32-1.45] | <0.0001 | <0.0001 | <0.0001 |
| **BSA[DB]** | 0.586 [0.557-0.616] | 0.628 [0.621-0.636] | 0.593 [0.581-0.604] | 1.32 [1.18-1.47] | 1.58 [1.54-1.62] | 1.38 [1.32-1.44] | <0.0001 | <0.0001 | <0.0001 |
| **LBM[H]** | 0.608 [0.578-0.638] | 0.622 [0.614-0.629] | 0.584 [0.573-0.596] | 1.39 [1.26-1.54] | 1.52 [1.48-1.56] | 1.36 [1.30-1.42] | <0.0001 | <0.0001 | <0.0001 |
| **LBM[B]** | 0.609 [0.579-0.639] | 0.616 [0.609-0.624] | 0.575 [0.564-0.587] | 1.42 [1.28-1.58] | 1.50 [1.46-1.53] | 1.32 [1.26-1.37] | <0.0001 | <0.0001 | <0.0001 |
| **LBM[J]** | 0.570 [0.542-0.599] | 0.617 [0.610-0.625] | 0.577 [0.566-0.589] | 1.16 [1.06-1.26] | 1.57 [1.52-1.61] | 1.14 [1.12-1.16] | 0.0011 | <0.0001 | <0.0001 |
| **BMI** | 0.529 [0.501-0.558] | 0.615 [0.607-0.622] | 0.570 [0.559-0.582] | 1.01 [0.94-1.09] | 1.57 [1.53-1.62] | 1.39 [1.31-1.47] | 0.82 | <0.0001 | <0.0001 |
| **Height^2.7** | 0.633 [0.603-0.664] | 0.619 [0.611-0.626] | 0.594 [0.583-0.606] | 1.68 [1.48-1.90] | 1.53 [1.49-1.57] | 1.32 [1.28-1.37] | <0.0001 | <0.0001 | <0.0001 |
| **Height^2.13** | 0.630 [0.600-0.660] | 0.620 [0.613-0.627] | 0.596 [0.585-0.608] | 1.71 [1.50-1.95] | 1.54 [1.50-1.59] | 1.33 [1.28-1.39] | <0.0001 | <0.0001 | <0.0001 |
| **Height^2** | 0.629 [0.599-0.659] | 0.620 [0.613-0.628] | 0.597 [0.585-0.608] | 1.71 [1.50-1.96] | 1.54 [1.50-1.59] | 1.33 [1.28-1.39] | <0.0001 | <0.0001 | <0.0001 |
| **Height^1.5** | 0.624 [0.593-0.654] | 0.620 [0.613-0.627] | 0.597 [0.586-0.609] | 1.72 [1.50-1.98] | 1.54 [1.49-1.58] | 1.33 [1.28-1.39] | <0.0001 | <0.0001 | <0.0001 |
| **Height** | 0.616 [0.586-0.647] | 0.617 [0.610-0.624] | 0.596 [0.584-0.608] | 1.70 [1.47-1.96] | 1.52 [1.47-1.56] | 1.32 [1.27-1.38] | <0.0001 | <0.0001 | <0.0001 |
| ***Unindexed*** | *0.594 [0.562-0.625]* | *0.602 [0.595-0.610]* | *0.585 [0.573-0.596]* | *1.56 [1.34-1.81]* | *1.44 [1.40-1.49]* | *1.29 [1.23-1.34]* | *<0.0001* | *<0.0001* | *<0.0001* |

**Supplementary Table 7.** Statistical analyses for 5-year cardiovascular mortality for IVS thickness indexed by different metrics.

BSA = body surface area, [M] = Mosteller, [DB] = Du Bois, LBM = lean body mass, [H] = Hume, [B] = Boer, [J] = James, BMI = body mass index.

| | C statistic | | | Hazard Ratio per 1 SD increment | | | p value | | |
|---|---|---|---|---|---|---|---|---|---|
| | BMI <18.5 kg/m$^2$ | BMI 18.5-30 kg/m$^2$ | BMI ≥30 kg/m$^2$ | BMI <18.5 kg/m$^2$ | BMI 18.5-30 kg/m$^2$ | BMI ≥30 kg/m$^2$ | BMI <18.5 kg/m$^2$ | BMI 18.5-30 kg/m$^2$ | BMI ≥30 kg/m$^2$ |
| **Weight** | 0.677 [0.658-0.695] | 0.695 [0.690-0.700] | 0.617 [0.608-0.627] | 1.30 [1.26-1.35] | 1.67 [1.65-1.69] | 1.71 [1.65-1.78] | <0.0001 | <0.0001 | <0.0001 |
| **Weight^1.5** | 0.654 [0.635-0.672] | 0.677 [0.672-0.682] | 0.602 [0.593-0.612] | 1.17 [1.14-1.21] | 1.71 [1.69-1.73] | 1.87 [1.79-1.96] | <0.0001 | <0.0001 | <0.0001 |
| **Height*Weight** | 0.671 [0.652-0.690] | 0.683 [0.678-0.688] | 0.611 [0.602-0.621] | 1.30 [1.26-1.34] | 1.64 [1.62-1.65] | 1.62 [1.57-1.68] | <0.0001 | <0.0001 | <0.0001 |
| **BSA[M]^1.5** | 0.686 [0.667-0.705] | 0.694 [0.689-0.699] | 0.621 [0.612-0.631] | 1.37 [1.32-1.42] | 1.62 [1.61-1.64] | 1.60 [1.55-1.66] | <0.0001 | <0.0001 | <0.0001 |
| **BSA[DB]^1.5** | 0.690 [0.671-0.709] | 0.692 [0.687-0.697] | 0.623 [0.613-0.632] | 1.40 [1.35-1.45] | 1.61 [1.59-1.63] | 1.56 [1.52-1.61] | <0.0001 | <0.0001 | <0.0001 |
| **BSA[M]** | 0.701 [0.682-0.720] | 0.699 [0.694-0.704] | 0.630 [0.620-0.639] | 1.43 [1.38-1.48] | 1.60 [1.58-1.61] | 1.55 [1.50-1.59] | <0.0001 | <0.0001 | <0.0001 |
| **BSA[DB]** | 0.704 [0.685-0.723] | 0.698 [0.693-0.703] | 0.631 [0.621-0.640] | 1.45 [1.39-1.50] | 1.59 [1.57-1.61] | 1.53 [1.49-1.57] | <0.0001 | <0.0001 | <0.0001 |
| **LBM[H]** | 0.698 [0.679-0.717] | 0.680 [0.675-0.685] | 0.614 [0.604-0.623] | 1.44 [1.39-1.49] | 1.55 [1.54-1.57] | 1.46 [1.43-1.50] | <0.0001 | <0.0001 | <0.0001 |
| **LBM[B]** | 0.700 [0.681-0.719] | 0.671 [0.666-0.676] | 0.595 [0.585-0.605] | 1.46 [1.41-1.52] | 1.54 [1.52-1.56] | 1.39 [1.36-1.43] | <0.0001 | <0.0001 | <0.0001 |
| **LBM[J]** | 0.677 [0.658-0.697] | 0.673 [0.668-0.678] | 0.596 [0.587-0.605] | 1.39 [1.33-1.44] | 1.61 [1.59-1.63] | 1.12 [1.11-1.13] | <0.0001 | <0.0001 | <0.0001 |
| **BMI** | 0.668 [0.649-0.687] | 0.691 [0.686-0.696] | 0.606 [0.597-0.616] | 1.27 [1.23-1.32] | 1.65 [1.63-1.67] | 1.62 [1.56-1.68] | <0.0001 | <0.0001 | <0.0001 |
| **Height^2.7** | 0.708 [0.689-0.727] | 0.675 [0.670-0.681] | 0.622 [0.613-0.632] | 1.58 [1.52-1.65] | 1.59 [1.57-1.61] | 1.40 [1.37-1.43] | <0.0001 | <0.0001 | <0.0001 |
| **Height^2.13** | 0.714 [0.695-0.733] | 0.682 [0.676-0.687] | 0.628 [0.619-0.638] | 1.58 [1.52-1.65] | 1.59 [1.57-1.61] | 1.44 [1.41-1.48] | <0.0001 | <0.0001 | <0.0001 |
| **Height^2** | 0.715 [0.697-0.734] | 0.683 [0.678-0.688] | 0.629 [0.620-0.639] | 1.58 [1.52-1.65] | 1.59 [1.57-1.61] | 1.45 [1.41-1.48] | <0.0001 | <0.0001 | <0.0001 |
| **Height^1.5** | 0.718 [0.700-0.737] | 0.685 [0.680-0.690] | 0.633 [0.623-0.642] | 1.59 [1.52-1.65] | 1.59 [1.57-1.61] | 1.46 [1.43-1.50] | <0.0001 | <0.0001 | <0.0001 |
| **Height** | 0.719 [0.700-0.738] | 0.685 [0.680-0.690] | 0.633 [0.624-0.642] | 1.59 [1.52-1.65] | 1.58 [1.56-1.60] | 1.46 [1.43-1.50] | <0.0001 | <0.0001 | <0.0001 |
| ***Unindexed*** | *0.710 [0.690-0.729]* | *0.672 [0.666-0.677]* | *0.623 [0.614-0.632]* | *1.58 [1.52-1.65]* | *1.54 [1.52-1.56]* | *1.43 [1.39-1.46]* | *<0.0001* | *<0.0001* | *<0.0001* |

**Supplementary Table 8.** Statistical analyses for 5-year cardiovascular mortality for LA area indexed by different metrics.

BSA = body surface area, [M] = Mosteller, [DB] = Du Bois, LBM = lean body mass, [H] = Hume, [B] = Boer, [J] = James, BMI = body mass index.

| | **C statistic** | | | **Hazard Ratio per 1 SD increment** | | | **p value** | | |
|---|---|---|---|---|---|---|---|---|---|
| | **BMI <18.5 kg/$m^2$** | **BMI 18.5-30 kg/$m^2$** | **BMI ≥30 kg/$m^2$** | **BMI <18.5 kg/$m^2$** | **BMI 18.5-30 kg/$m^2$** | **BMI ≥30 kg/$m^2$** | **BMI <18.5 kg/$m^2$** | **BMI 18.5-30 kg/$m^2$** | **BMI ≥30 kg/$m^2$** |
| **Weight** | 0.734 [0.706-0.762] | 0.705 [0.698-0.713] | 0.626 [0.613-0.639] | 1.24 [1.21-1.28] | 1.43 [1.42-1.45] | 1.76 [1.68-1.84] | <0.0001 | <0.0001 | <0.0001 |
| **Weight^1.5** | 0.731 [0.704-0.759] | 0.697 [0.690-0.705] | 0.618 [0.605-0.631] | 1.22 [1.19-1.25] | 1.51 [1.50-1.53] | 1.96 [1.86-2.08] | <0.0001 | <0.0001 | <0.0001 |
| **Height*Weight** | 0.731 [0.703-0.758] | 0.703 [0.695-0.710] | 0.627 [0.614-0.640] | 1.26 [1.23-1.29] | 1.46 [1.45-1.48] | 1.75 [1.67-1.83] | <0.0001 | <0.0001 | <0.0001 |
| **BSA[M]^1.5** | 0.732 [0.705-0.760] | 0.706 [0.698-0.713] | 0.631 [0.618-0.644] | 1.27 [1.24-1.31] | 1.41 [1.40-1.43] | 1.68 [1.62-1.75] | <0.0001 | <0.0001 | <0.0001 |
| **BSA[DB]^1.5** | 0.732 [0.704-0.759] | 0.706 [0.698-0.713] | 0.633 [0.620-0.645] | 1.29 [1.25-1.33] | 1.41 [1.39-1.42] | 1.65 [1.59-1.71] | <0.0001 | <0.0001 | <0.0001 |
| **BSA[M]** | 0.731 [0.703-0.759] | 0.704 [0.696-0.711] | 0.632 [0.619-0.645] | 1.29 [1.25-1.33] | 1.37 [1.36-1.38] | 1.61 [1.55-1.67] | <0.0001 | <0.0001 | <0.0001 |
| **BSA[DB]** | 0.731 [0.703-0.759] | 0.703 [0.696-0.711] | 0.633 [0.621-0.646] | 1.30 [1.27-1.34] | 1.37 [1.35-1.38] | 1.59 [1.54-1.65] | <0.0001 | <0.0001 | <0.0001 |
| **LBM[H]** | 0.726 [0.698-0.754] | 0.699 [0.691-0.706] | 0.627 [0.615-0.640] | 1.34 [1.30-1.39] | 1.41 [1.39-1.42] | 1.59 [1.53-1.65] | <0.0001 | <0.0001 | <0.0001 |
| **LBM[B]** | 0.727 [0.699-0.755] | 0.693 [0.686-0.701] | 0.616 [0.603-0.628] | 1.35 [1.30-1.39] | 1.43 [1.41-1.44] | 1.52 [1.47-1.58] | <0.0001 | <0.0001 | <0.0001 |
| **LBM[J]** | 0.726 [0.698-0.754] | 0.695 [0.687-0.702] | 0.615 [0.602-0.628] | 1.32 [1.28-1.37] | 1.46 [1.44-1.48] | 1.11 [1.09-1.12] | <0.0001 | <0.0001 | <0.0001 |
| **BMI** | 0.724 [0.695-0.753] | 0.690 [0.683-0.698] | 0.611 [0.598-0.623] | 1.23 [1.20-1.26] | 1.37 [1.36-1.39] | 1.64 [1.56-1.71] | <0.0001 | <0.0001 | <0.0001 |
| **Height^2.7** | 0.725 [0.697-0.753] | 0.697 [0.690-0.705] | 0.635 [0.623-0.648] | 1.45 [1.39-1.51] | 1.41 [1.39-1.42] | 1.48 [1.44-1.53] | <0.0001 | <0.0001 | <0.0001 |
| **Height^2.13** | 0.727 [0.698-0.755] | 0.698 [0.690-0.705] | 0.636 [0.624-0.649] | 1.43 [1.37-1.48] | 1.39 [1.37-1.40] | 1.50 [1.46-1.55] | <0.0001 | <0.0001 | <0.0001 |
| **Height^2** | 0.727 [0.699-0.755] | 0.697 [0.690-0.705] | 0.636 [0.624-0.649] | 1.42 [1.37-1.48] | 1.38 [1.37-1.39] | 1.51 [1.46-1.55] | <0.0001 | <0.0001 | <0.0001 |
| **Height^1.5** | 0.727 [0.698-0.755] | 0.695 [0.688-0.703] | 0.635 [0.622-0.647] | 1.40 [1.35-1.46] | 1.37 [1.35-1.38] | 1.51 [1.46-1.55] | <0.0001 | <0.0001 | <0.0001 |
| **Height** | 0.725 [0.697-0.754] | 0.692 [0.684-0.699] | 0.632 [0.619-0.644] | 1.39 [1.33-1.44] | 1.35 [1.34-1.37] | 1.50 [1.46-1.55] | <0.0001 | <0.0001 | <0.0001 |
| ***Unindexed*** | *0.718 [0.688-0.747]* | *0.679 [0.671-0.686]* | 0.622 [0.609-0.634] | *1.36 [1.31-1.41]* | *1.34 [1.33-1.35]* | *1.47 [1.42-1.51]* | *<0.0001* | *<0.0001* | *<0.0001* |

**Supplementary Table 9.** Statistical analyses for 5-year cardiovascular mortality for LA diameter indexed by different metrics.

BSA = body surface area, [M] = Mosteller, [DB] = Du Bois, LBM = lean body mass, [H] = Hume, [B] = Boer, [J] = James, BMI = body mass index.

| | **C statistic** | | | **Hazard Ratio per 1 SD increment** | | | **p value** | | |
|---|---|---|---|---|---|---|---|---|---|
| | **BMI <18.5 kg/m$^2$** | **BMI 18.5-30 kg/m$^2$** | **BMI ≥30 kg/m$^2$** | **BMI <18.5 kg/m$^2$** | **BMI 18.5-30 kg/m$^2$** | **BMI ≥30 kg/m$^2$** | **BMI <18.5 kg/m$^2$** | **BMI 18.5-30 kg/m$^2$** | **BMI ≥30 kg/m$^2$** |
| **Weight** | 0.723 [0.690-0.755] | 0.704 [0.696-0.712] | 0.652 [0.637-0.666] | 1.40 [1.33-1.48] | 1.81 [1.77-1.84] | 2.20 [2.06-2.35] | <0.0001 | <0.0001 | <0.0001 |
| **Weight^1.5** | 0.714 [0.681-0.746] | 0.680 [0.672-0.689] | 0.628 [0.614-0.643] | 1.25 [1.20-1.30] | 1.73 [1.69-1.77] | 2.34 [2.16-2.55] | <0.0001 | <0.0001 | <0.0001 |
| **Height*Weight** | 0.721 [0.688-0.754] | 0.691 [0.683-0.699] | 0.640 [0.626-0.654] | 1.41 [1.33-1.48] | 1.69 [1.66-1.73] | 1.98 [1.86-2.10] | <0.0001 | <0.0001 | <0.0001 |
| **BSA[M]^1.5** | 0.728 [0.694-0.761] | 0.705 [0.697-0.713] | 0.656 [0.642-0.670] | 1.49 [1.40-1.58] | 1.76 [1.72-1.79] | 2.00 [1.89-2.11] | <0.0001 | <0.0001 | <0.0001 |
| **BSA[DB]^1.5** | 0.729 [0.696-0.763] | 0.704 [0.696-0.712] | 0.657 [0.643-0.671] | 1.52 [1.43-1.61] | 1.74 [1.70-1.77] | 1.91 [1.82-2.02] | <0.0001 | <0.0001 | <0.0001 |
| **BSA[M]** | 0.729 [0.695-0.763] | 0.713 [0.705-0.721] | 0.671 [0.657-0.685] | 1.56 [1.47-1.67] | 1.81 [1.77-1.84] | 1.98 [1.88-2.08] | <0.0001 | <0.0001 | <0.0001 |
| **BSA[DB]** | 0.730 [0.697-0.764] | 0.713 [0.705-0.721] | 0.672 [0.658-0.686] | 1.58 [1.48-1.69] | 1.80 [1.76-1.84] | 1.94 [1.85-2.04] | <0.0001 | <0.0001 | <0.0001 |
| **LBM[H]** | 0.727 [0.693-0.762] | 0.693 [0.685-0.701] | 0.646 [0.632-0.660] | 1.55 [1.46-1.65] | 1.66 [1.63-1.69] | 1.74 [1.66-1.83] | <0.0001 | <0.0001 | <0.0001 |
| **LBM[B]** | 0.727 [0.693-0.761] | 0.682 [0.674-0.690] | 0.621 [0.607-0.635] | 1.58 [1.49-1.68] | 1.63 [1.60-1.67] | 1.58 [1.51-1.66] | <0.0001 | <0.0001 | <0.0001 |
| **LBM[J]** | 0.718 [0.685-0.752] | 0.683 [0.675-0.692] | 0.618 [0.604-0.632] | 1.53 [1.43-1.63] | 1.77 [1.73-1.81] | 1.11 [1.10-1.13] | <0.0001 | <0.0001 | <0.0001 |
| **BMI** | 0.696 [0.662-0.730] | 0.696 [0.688-0.704] | 0.644 [0.630-0.659] | 1.38 [1.29-1.47] | 1.89 [1.84-1.93] | 2.09 [1.96-2.23] | <0.0001 | <0.0001 | <0.0001 |
| **Height^2.7** | 0.731 [0.697-0.766] | 0.692 [0.684-0.700] | 0.652 [0.638-0.666] | 1.72 [1.60-1.85] | 1.76 [1.72-1.80] | 1.51 [1.46-1.56] | <0.0001 | <0.0001 | <0.0001 |
| **Height^2.13** | 0.733 [0.698-0.767] | 0.699 [0.691-0.707] | 0.663 [0.650-0.677] | 1.74 [1.62-1.87] | 1.81 [1.77-1.85] | 1.66 [1.60-1.72] | <0.0001 | <0.0001 | <0.0001 |
| **Height^2** | 0.732 [0.698-0.767] | 0.700 [0.692-0.708] | 0.666 [0.652-0.679] | 1.74 [1.62-1.87] | 1.82 [1.77-1.86] | 1.69 [1.63-1.76] | <0.0001 | <0.0001 | <0.0001 |
| **Height^1.5** | 0.731 [0.696-0.765] | 0.703 [0.695-0.711] | 0.673 [0.659-0.687] | 1.76 [1.63-1.90] | 1.85 [1.80-1.89] | 1.81 [1.73-1.89] | <0.0001 | <0.0001 | <0.0001 |
| **Height** | 0.725 [0.690-0.760] | 0.702 [0.693-0.710] | 0.677 [0.663-0.690] | 1.79 [1.65-1.93] | 1.86 [1.81-1.90] | 1.87 [1.78-1.95] | <0.0001 | <0.0001 | <0.0001 |
| ***Unindexed*** | *0.704 [0.668-0.740]* | *0.686 [0.678-0.694]* | 0.667 [0.653-0.681] | *1.83 [1.66-2.01]* | *1.79 [1.75-1.84]* | *1.80 [1.72-1.88]* | *<0.0001* | *<0.0001* | *<0.0001* |

**Supplementary Table 10.** Statistical analyses for 5-year cardiovascular mortality for LA volume indexed by different metrics.

BSA = body surface area, [M] = Mosteller, [DB] = Du Bois, LBM = lean body mass, [H] = Hume, [B] = Boer, [J] = James, BMI = body mass index.

| | **C statistic** | | | **Hazard Ratio per 1 SD increment** | | | **p value** | | |
|---|---|---|---|---|---|---|---|---|---|
| | **BMI <18.5 kg/m$^2$** | **BMI 18.5-30 kg/m$^2$** | **BMI ≥30 kg/m$^2$** | **BMI <18.5 kg/m$^2$** | **BMI 18.5-30 kg/m$^2$** | **BMI ≥30 kg/m$^2$** | **BMI <18.5 kg/m$^2$** | **BMI 18.5-30 kg/m$^2$** | **BMI ≥30 kg/m$^2$** |
| **Weight** | 0.725 [0.703-0.747] | 0.731 [0.726-0.737] | 0.670 [0.660-0.681] | 1.41 [1.36-1.47] | 1.57 [1.55-1.59] | 1.57 [1.51-1.62] | <0.0001 | <0.0001 | <0.0001 |
| **Weight^1.5** | 0.726 [0.704-0.748] | 0.733 [0.728-0.739] | 0.667 [0.656-0.677] | 1.33 [1.28-1.37] | 1.55 [1.53-1.56] | 1.69 [1.63-1.76] | <0.0001 | <0.0001 | <0.0001 |
| **Height*Weight** | 0.724 [0.702-0.746] | 0.733 [0.727-0.739] | 0.672 [0.662-0.683] | 1.39 [1.34-1.45] | 1.56 [1.54-1.58] | 1.57 [1.52-1.63] | <0.0001 | <0.0001 | <0.0001 |
| **BSA[M]^1.5** | 0.724 [0.702-0.746] | 0.731 [0.725-0.736] | 0.673 [0.663-0.684] | 1.45 [1.39-1.51] | 1.58 [1.56-1.60] | 1.52 [1.48-1.57] | <0.0001 | <0.0001 | <0.0001 |
| **BSA[DB]^1.5** | 0.723 [0.701-0.746] | 0.730 [0.724-0.736] | 0.674 [0.664-0.685] | 1.47 [1.41-1.54] | 1.58 [1.56-1.60] | 1.51 [1.46-1.55] | <0.0001 | <0.0001 | <0.0001 |
| **BSA[M]** | 0.722 [0.700-0.744] | 0.726 [0.720-0.731] | 0.672 [0.662-0.683] | 1.53 [1.45-1.60] | 1.59 [1.57-1.61] | 1.48 [1.43-1.52] | <0.0001 | <0.0001 | <0.0001 |
| **BSA[DB]** | 0.721 [0.699-0.744] | 0.725 [0.719-0.731] | 0.673 [0.663-0.683] | 1.54 [1.47-1.62] | 1.59 [1.57-1.62] | 1.47 [1.43-1.51] | <0.0001 | <0.0001 | <0.0001 |
| **LBM[H]** | 0.719 [0.696-0.741] | 0.726 [0.720-0.731] | 0.672 [0.661-0.682] | 1.51 [1.44-1.59] | 1.58 [1.56-1.60] | 1.48 [1.44-1.52] | <0.0001 | <0.0001 | <0.0001 |
| **LBM[B]** | 0.719 [0.697-0.742] | 0.723 [0.718-0.729] | 0.667 [0.656-0.677] | 1.52 [1.45-1.59] | 1.57 [1.55-1.59] | 1.46 [1.42-1.50] | <0.0001 | <0.0001 | <0.0001 |
| **LBM[J]** | 0.719 [0.697-0.742] | 0.724 [0.718-0.730] | 0.666 [0.656-0.677] | 1.50 [1.43-1.57] | 1.61 [1.59-1.63] | 1.14 [1.13-1.15] | <0.0001 | <0.0001 | <0.0001 |
| **BMI** | 0.719 [0.696-0.741] | 0.719 [0.713-0.725] | 0.657 [0.647-0.668] | 1.44 [1.38-1.50] | 1.54 [1.52-1.57] | 1.51 [1.46-1.56] | <0.0001 | <0.0001 | <0.0001 |
| **Height^2.7** | 0.718 [0.696-0.741] | 0.723 [0.717-0.729] | 0.677 [0.666-0.687] | 1.67 [1.57-1.77] | 1.62 [1.60-1.64] | 1.42 [1.38-1.46] | <0.0001 | <0.0001 | <0.0001 |
| **Height^2.13** | 0.719 [0.696-0.741] | 0.721 [0.716-0.727] | 0.676 [0.665-0.686] | 1.68 [1.58-1.79] | 1.62 [1.60-1.65] | 1.42 [1.38-1.46] | <0.0001 | <0.0001 | <0.0001 |
| **Height^2** | 0.718 [0.696-0.741] | 0.721 [0.715-0.727] | 0.675 [0.665-0.686] | 1.69 [1.59-1.79] | 1.62 [1.60-1.65] | 1.42 [1.38-1.46] | <0.0001 | <0.0001 | <0.0001 |
| **Height^1.5** | 0.718 [0.695-0.740] | 0.719 [0.713-0.724] | 0.673 [0.663-0.684] | 1.70 [1.60-1.81] | 1.62 [1.59-1.64] | 1.42 [1.38-1.45] | <0.0001 | <0.0001 | <0.0001 |
| **Height** | 0.716 [0.694-0.739] | 0.715 [0.710-0.721] | 0.671 [0.660-0.681] | 1.71 [1.61-1.82] | 1.61 [1.59-1.64] | 1.41 [1.37-1.45] | <0.0001 | <0.0001 | <0.0001 |
| ***Unindexed*** | *0.712 [0.689-0.735]* | *0.707 [0.701-0.713]* | *0.663 [0.653-0.674]* | *1.74 [1.63-1.85]* | *1.58 [1.56-1.61]* | *1.39 [1.35-1.43]* | *<0.0001* | *<0.0001* | *<0.0001* |

**Supplementary Table 11.** Statistical analyses for 5-year cardiovascular mortality for LV end-diastolic diameter indexed by different metrics.

BSA = body surface area, [M] = Mosteller, [DB] = Du Bois, LBM = lean body mass, [H] = Hume, [B] = Boer, [J] = James, BMI = body mass index.

| | **C statistic** | | | **Hazard Ratio per 1 SD increment** | | | **p value** | | |
|---|---|---|---|---|---|---|---|---|---|
| | **BMI <18.5 kg/m$^2$** | **BMI 18.5-30 kg/m$^2$** | **BMI ≥30 kg/m$^2$** | **BMI <18.5 kg/m$^2$** | **BMI 18.5-30 kg/m$^2$** | **BMI ≥30 kg/m$^2$** | **BMI <18.5 kg/m$^2$** | **BMI 18.5-30 kg/m$^2$** | **BMI ≥30 kg/m$^2$** |
| **Weight** | 0.545 [0.523-0.568] | 0.611 [0.605-0.617] | 0.583 [0.573-0.593] | 1.04 [1.00-1.09] | 1.65 [1.62-1.68] | 1.68 [1.61-1.75] | 0.066 | <0.0001 | <0.0001 |
| **Weight^1.5** | 0.549 [0.528-0.569] | 0.609 [0.604-0.615] | 0.573 [0.563-0.583] | 1.01 [0.97-1.04] | 1.63 [1.59-1.66] | 1.67 [1.59-1.75] | 0.70 | <0.0001 | <0.0001 |
| **Height*Weight** | 0.552 [0.530-0.573] | 0.611 [0.606-0.617] | 0.582 [0.573-0.592] | 1.07 [1.02-1.12] | 1.57 [1.54-1.60] | 1.41 [1.38-1.45] | 0.0025 | <0.0001 | <0.0001 |
| **BSA[M]^1.5** | 0.550 [0.528-0.573] | 0.611 [0.605-0.617] | 0.590 [0.580-0.599] | 1.10 [1.05-1.15] | 1.59 [1.56-1.62] | 1.48 [1.44-1.52] | 0.0001 | <0.0001 | <0.0001 |
| **BSA[DB]^1.5** | 0.554 [0.531-0.576] | 0.610 [0.604-0.616] | 0.592 [0.582-0.602] | 1.13 [1.07-1.19] | 1.56 [1.53-1.58] | 1.40 [1.37-1.44] | <0.0001 | <0.0001 | <0.0001 |
| **BSA[M]** | 0.547 [0.524-0.571] | 0.603 [0.597-0.609] | 0.593 [0.583-0.604] | 1.13 [1.07-1.19] | 1.55 [1.53-1.58] | 1.53 [1.48-1.58] | <0.0001 | <0.0001 | <0.0001 |
| **BSA[DB]** | 0.551 [0.527-0.575] | 0.602 [0.596-0.608] | 0.596 [0.586-0.606] | 1.16 [1.10-1.22] | 1.54 [1.51-1.56] | 1.49 [1.45-1.53] | <0.0001 | <0.0001 | <0.0001 |
| **LBM[H]** | 0.560 [0.537-0.584] | 0.597 [0.591-0.603] | 0.582 [0.573-0.592] | 1.22 [1.16-1.29] | 1.44 [1.42-1.46] | 1.31 [1.28-1.34] | <0.0001 | <0.0001 | <0.0001 |
| **LBM[B]** | 0.561 [0.538-0.585] | 0.588 [0.582-0.594] | 0.562 [0.552-0.572] | 1.25 [1.17-1.32] | 1.40 [1.38-1.43] | 1.28 [1.24-1.31] | <0.0001 | <0.0001 | <0.0001 |
| **LBM[J]** | 0.539 [0.517-0.562] | 0.588 [0.582-0.594] | 0.562 [0.552-0.571] | 1.08 [1.02-1.14] | 1.49 [1.46-1.52] | 1.07 [1.06-1.08] | 0.0038 | <0.0001 | <0.0001 |
| **BMI** | 0.489 [0.466-0.513] | 0.581 [0.575-0.587] | 0.558 [0.548-0.568] | 0.98 [0.93-1.02] | 1.54 [1.51-1.57] | 1.54 [1.47-1.62] | 0.34 | <0.0001 | <0.0001 |
| **Height^2.7** | 0.578 [0.555-0.602] | 0.595 [0.589-0.600] | 0.596 [0.586-0.606] | 1.37 [1.28-1.47] | 1.46 [1.44-1.49] | 1.22 [1.20-1.24] | <0.0001 | <0.0001 | <0.0001 |
| **Height^2.13** | 0.574 [0.550-0.598] | 0.593 [0.587-0.599] | 0.600 [0.590-0.610] | 1.35 [1.27-1.45] | 1.48 [1.46-1.51] | 1.29 [1.27-1.31] | <0.0001 | <0.0001 | <0.0001 |
| **Height^2** | 0.573 [0.549-0.598] | 0.592 [0.586-0.598] | 0.600 [0.590-0.610] | 1.35 [1.26-1.44] | 1.48 [1.46-1.51] | 1.31 [1.29-1.33] | <0.0001 | <0.0001 | <0.0001 |
| **Height^1.5** | 0.567 [0.542-0.592] | 0.586 [0.580-0.592] | 0.599 [0.589-0.610] | 1.32 [1.24-1.41] | 1.47 [1.45-1.50] | 1.39 [1.36-1.42] | <0.0001 | <0.0001 | <0.0001 |
| **Height** | 0.557 [0.532-0.583] | 0.577 [0.570-0.583] | 0.594 [0.583-0.604] | 1.29 [1.20-1.38] | 1.44 [1.41-1.46] | 1.45 [1.41-1.49] | <0.0001 | <0.0001 | <0.0001 |
| ***Unindexed*** | *0.533 [0.508-0.559]* | *0.553 [0.546-0.559]* | 0.572 [0.561-0.582] | *1.19 [1.10-1.28]* | *1.31 [1.29-1.34]* | *1.38 [1.34-1.43]* | *<0.0001* | *<0.0001* | *<0.0001* |

**Supplementary Table 12.** Statistical analyses for 5-year cardiovascular mortality for LV end-diastolic volume indexed by different metrics.

BSA = body surface area, [M] = Mosteller, [DB] = Du Bois, LBM = lean body mass, [H] = Hume, [B] = Boer, [J] = James, BMI = body mass index.

| | **C statistic** | | | **Hazard Ratio per 1 SD increment** | | | **p value** | | |
|---|---|---|---|---|---|---|---|---|---|
| | **BMI <18.5 kg/m$^2$** | **BMI 18.5-30 kg/m$^2$** | **BMI ≥30 kg/m$^2$** | **BMI <18.5 kg/m$^2$** | **BMI 18.5-30 kg/m$^2$** | **BMI ≥30 kg/m$^2$** | **BMI <18.5 kg/m$^2$** | **BMI 18.5-30 kg/m$^2$** | **BMI ≥30 kg/m$^2$** |
| **Weight** | 0.553 [0.503-0.603] | 0.598 [0.586-0.610] | 0.627 [0.602-0.651] | 1.22 [1.12-1.33] | 1.44 [1.41-1.47] | 1.78 [1.68-1.88] | <0.0001 | <0.0001 | <0.0001 |
| **Weight^1.5** | 0.575 [0.527-0.624] | 0.615 [0.603-0.627] | 0.634 [0.611-0.658] | 1.21 [1.13-1.30] | 1.49 [1.46-1.52] | 2.05 [1.92-2.19] | <0.0001 | <0.0001 | <0.0001 |
| **Height*Weight** | 0.568 [0.518-0.617] | 0.607 [0.595-0.619] | 0.637 [0.612-0.661] | 1.24 [1.14-1.35] | 1.46 [1.43-1.50] | 1.80 [1.70-1.90] | <0.0001 | <0.0001 | <0.0001 |
| **BSA[M]^1.5** | 0.552 [0.502-0.602] | 0.595 [0.583-0.608] | 0.630 [0.605-0.654] | 1.24 [1.13-1.36] | 1.44 [1.41-1.47] | 1.70 [1.62-1.79] | <0.0001 | <0.0001 | <0.0001 |
| **BSA[DB]^1.5** | 0.552 [0.502-0.602] | 0.594 [0.582-0.606] | 0.631 [0.606-0.656] | 1.25 [1.14-1.37] | 1.43 [1.40-1.46] | 1.67 [1.59-1.75] | <0.0001 | <0.0001 | <0.0001 |
| **BSA[M]** | 0.537 [0.486-0.588] | 0.582 [0.570-0.594] | 0.621 [0.596-0.646] | 1.24 [1.12-1.37] | 1.41 [1.37-1.44] | 1.62 [1.54-1.70] | <0.0001 | <0.0001 | <0.0001 |
| **BSA[DB]** | 0.537 [0.486-0.588] | 0.581 [0.569-0.593] | 0.622 [0.597-0.647] | 1.25 [1.12-1.39] | 1.41 [1.37-1.44] | 1.60 [1.53-1.68] | <0.0001 | <0.0001 | <0.0001 |
| **LBM[H]** | 0.548 [0.498-0.598] | 0.586 [0.574-0.598] | 0.629 [0.605-0.654] | 1.26 [1.13-1.39] | 1.42 [1.39-1.45] | 1.63 [1.55-1.71] | <0.0001 | <0.0001 | <0.0001 |
| **LBM[B]** | 0.547 [0.496-0.597] | 0.584 [0.572-0.596] | 0.627 [0.603-0.652] | 1.25 [1.13-1.39] | 1.41 [1.38-1.45] | 1.62 [1.54-1.70] | <0.0001 | <0.0001 | <0.0001 |
| **LBM[J]** | 0.548 [0.498-0.598] | 0.585 [0.572-0.597] | 0.622 [0.597-0.646] | 1.23 [1.12-1.36] | 1.42 [1.39-1.45] | 1.47 [1.41-1.52] | <0.0001 | <0.0001 | <0.0001 |
| **BMI** | 0.526 [0.476-0.576] | 0.577 [0.565-0.589] | 0.603 [0.578-0.628] | 1.17 [1.07-1.29] | 1.38 [1.35-1.41] | 1.67 [1.57-1.78] | 0.0005 | <0.0001 | <0.0001 |
| **Height^2.7** | 0.548 [0.498-0.599] | 0.581 [0.569-0.594] | 0.632 [0.607-0.656] | 1.32 [1.17-1.49] | 1.43 [1.40-1.46] | 1.55 [1.48-1.61] | <0.0001 | <0.0001 | <0.0001 |
| **Height^2.13** | 0.540 [0.489-0.590] | 0.576 [0.564-0.588] | 0.627 [0.602-0.651] | 1.31 [1.15-1.48] | 1.41 [1.38-1.45] | 1.54 [1.48-1.61] | <0.0001 | <0.0001 | <0.0001 |
| **Height^2** | 0.538 [0.487-0.588] | 0.575 [0.563-0.587] | 0.625 [0.600-0.650] | 1.30 [1.15-1.48] | 1.41 [1.38-1.44] | 1.54 [1.48-1.61] | <0.0001 | <0.0001 | <0.0001 |
| **Height^1.5** | 0.530 [0.479-0.581] | 0.570 [0.558-0.582] | 0.620 [0.595-0.645] | 1.29 [1.13-1.46] | 1.39 [1.36-1.43] | 1.53 [1.47-1.60] | 0.0001 | <0.0001 | <0.0001 |
| **Height** | 0.524 [0.473-0.575] | 0.565 [0.553-0.577] | 0.614 [0.589-0.639] | 1.27 [1.11-1.45] | 1.38 [1.34-1.41] | 1.51 [1.45-1.58] | 0.0004 | <0.0001 | <0.0001 |
| ***Unindexed*** | *0.512 [0.461-0.563]* | *0.556 [0.544-0.568]* | 0.601 [0.576-0.626] | *1.24 [1.07-1.42]* | *1.34 [1.30-1.37]* | *1.48 [1.41-1.55]* | *0.0033* | *<0.0001* | *<0.0001* |

**Supplementary Table 13.** Statistical analyses for 5-year cardiovascular mortality for LV mass indexed by different metrics.

BSA = body surface area, [M] = Mosteller, [DB] = Du Bois, LBM = lean body mass, [H] = Hume, [B] = Boer, [J] = James, BMI = body mass index.

| | C statistic | | | Hazard Ratio per 1 SD increment | | | p value | | |
|---|---|---|---|---|---|---|---|---|---|
| | **BMI <18.5 kg/m$^2$** | **BMI 18.5-30 kg/m$^2$** | **BMI ≥30 kg/m$^2$** | **BMI <18.5 kg/m$^2$** | **BMI 18.5-30 kg/m$^2$** | **BMI ≥30 kg/m$^2$** | **BMI <18.5 kg/m$^2$** | **BMI 18.5-30 kg/m$^2$** | **BMI ≥30 kg/m$^2$** |
| **Weight** | 0.697 [0.677-0.717] | 0.723 [0.718-0.728] | 0.664 [0.655-0.674] | 1.42 [1.37-1.47] | 1.77 [1.75-1.80] | 1.83 [1.77-1.88] | <0.0001 | <0.0001 | <0.0001 |
| **Weight^1.5** | 0.686 [0.667-0.706] | 0.728 [0.723-0.732] | 0.660 [0.651-0.670] | 1.25 [1.22-1.29] | 1.77 [1.75-1.79] | 1.97 [1.91-2.04] | <0.0001 | <0.0001 | <0.0001 |
| **Height*Weight** | 0.701 [0.682-0.721] | 0.728 [0.723-0.733] | 0.670 [0.661-0.679] | 1.42 [1.37-1.47] | 1.74 [1.72-1.76] | 1.69 [1.66-1.73] | <0.0001 | <0.0001 | <0.0001 |
| **BSA[M]^1.5** | 0.707 [0.687-0.727] | 0.722 [0.717-0.727] | 0.670 [0.661-0.679] | 1.53 [1.47-1.59] | 1.77 [1.75-1.79] | 1.74 [1.69-1.79] | <0.0001 | <0.0001 | <0.0001 |
| **BSA[DB]^1.5** | 0.711 [0.691-0.731] | 0.721 [0.716-0.726] | 0.671 [0.662-0.681] | 1.58 [1.51-1.65] | 1.77 [1.75-1.79] | 1.70 [1.65-1.74] | <0.0001 | <0.0001 | <0.0001 |
| **BSA[M]** | 0.709 [0.689-0.730] | 0.710 [0.705-0.715] | 0.665 [0.655-0.674] | 1.66 [1.59-1.74] | 1.77 [1.75-1.80] | 1.68 [1.64-1.73] | <0.0001 | <0.0001 | <0.0001 |
| **BSA[DB]** | 0.712 [0.691-0.732] | 0.709 [0.704-0.714] | 0.666 [0.657-0.675] | 1.71 [1.63-1.79] | 1.78 [1.76-1.80] | 1.67 [1.63-1.72] | <0.0001 | <0.0001 | <0.0001 |
| **LBM[H]** | 0.719 [0.698-0.739] | 0.715 [0.710-0.720] | 0.669 [0.660-0.678] | 1.67 [1.60-1.74] | 1.75 [1.73-1.77] | 1.63 [1.59-1.67] | <0.0001 | <0.0001 | <0.0001 |
| **LBM[B]** | 0.719 [0.698-0.739] | 0.712 [0.707-0.717] | 0.662 [0.653-0.671] | 1.67 [1.60-1.75] | 1.73 [1.71-1.75] | 1.60 [1.56-1.64] | <0.0001 | <0.0001 | <0.0001 |
| **LBM[J]** | 0.703 [0.682-0.723] | 0.714 [0.709-0.719] | 0.659 [0.650-0.668] | 1.59 [1.52-1.66] | 1.82 [1.80-1.85] | 1.09 [1.08-1.09] | <0.0001 | <0.0001 | <0.0001 |
| **BMI** | 0.678 [0.657-0.698] | 0.693 [0.688-0.698] | 0.637 [0.627-0.646] | 1.38 [1.33-1.44] | 1.72 [1.69-1.74] | 1.68 [1.63-1.74] | <0.0001 | <0.0001 | <0.0001 |
| **Height^2.7** | 0.726 [0.706-0.747] | 0.706 [0.701-0.711] | 0.672 [0.663-0.681] | 1.99 [1.88-2.11] | 1.82 [1.80-1.85] | 1.46 [1.44-1.49] | <0.0001 | <0.0001 | <0.0001 |
| **Height^2.13** | 0.724 [0.704-0.745] | 0.702 [0.697-0.707] | 0.669 [0.660-0.678] | 2.03 [1.91-2.15] | 1.83 [1.80-1.85] | 1.55 [1.52-1.59] | <0.0001 | <0.0001 | <0.0001 |
| **Height^2** | 0.724 [0.703-0.744] | 0.701 [0.696-0.706] | 0.668 [0.659-0.677] | 2.04 [1.92-2.16] | 1.83 [1.80-1.85] | 1.56 [1.53-1.60] | <0.0001 | <0.0001 | <0.0001 |
| **Height^1.5** | 0.720 [0.699-0.740] | 0.695 [0.690-0.700] | 0.663 [0.654-0.672] | 2.05 [1.93-2.18] | 1.81 [1.79-1.84] | 1.59 [1.55-1.63] | <0.0001 | <0.0001 | <0.0001 |
| **Height** | 0.715 [0.694-0.736] | 0.688 [0.683-0.693] | 0.657 [0.648-0.666] | 2.06 [1.93-2.19] | 1.79 [1.76-1.82] | 1.58 [1.54-1.62] | <0.0001 | <0.0001 | <0.0001 |
| ***Unindexed*** | *0.702 [0.681-0.723]* | *0.670 [0.665-0.676]* | 0.641 [0.632-0.651] | *2.02 [1.89-2.15]* | *1.72 [1.69-1.74]* | *1.52 [1.48-1.56]* | *<0.0001* | *<0.0001* | *<0.0001* |

**Supplementary Table 14.** Statistical analyses for 5-year cardiovascular mortality for RA area indexed by different metrics.

BSA = body surface area, [M] = Mosteller, [DB] = Du Bois, LBM = lean body mass, [H] = Hume, [B] = Boer, [J] = James, BMI = body mass index.

| | **C statistic** | | | **Hazard Ratio per 1 SD increment** | | | **p value** | | |
|---|---|---|---|---|---|---|---|---|---|
| | **BMI <18.5 kg/m²** | **BMI 18.5-30 kg/m²** | **BMI ≥30 kg/m²** | **BMI <18.5 kg/m²** | **BMI 18.5-30 kg/m²** | **BMI ≥30 kg/m²** | **BMI <18.5 kg/m²** | **BMI 18.5-30 kg/m²** | **BMI ≥30 kg/m²** |
| **Weight** | 0.704 [0.666-0.742] | 0.668 [0.658-0.679] | 0.591 [0.574-0.608] | 1.42 [1.33-1.50] | 1.64 [1.61-1.68] | 1.61 [1.52-1.71] | <0.0001 | <0.0001 | <0.0001 |
| **Weight^1.5** | 0.708 [0.671-0.746] | 0.671 [0.661-0.681] | 0.589 [0.573-0.606] | 1.37 [1.29-1.44] | 1.63 [1.60-1.67] | 1.75 [1.63-1.88] | <0.0001 | <0.0001 | <0.0001 |
| **Height*Weight** | 0.705 [0.667-0.743] | 0.672 [0.662-0.682] | 0.593 [0.576-0.610] | 1.40 [1.32-1.48] | 1.63 [1.60-1.67] | 1.61 [1.52-1.70] | <0.0001 | <0.0001 | <0.0001 |
| **BSA[M]^1.5** | 0.702 [0.664-0.740] | 0.669 [0.659-0.679] | 0.593 [0.576-0.610] | 1.44 [1.35-1.53] | 1.64 [1.60-1.68] | 1.56 [1.48-1.65] | <0.0001 | <0.0001 | <0.0001 |
| **BSA[DB]^1.5** | 0.701 [0.662-0.739] | 0.669 [0.658-0.679] | 0.594 [0.577-0.610] | 1.45 [1.36-1.54] | 1.64 [1.60-1.67] | 1.54 [1.46-1.62] | <0.0001 | <0.0001 | <0.0001 |
| **BSA[M]** | 0.695 [0.656-0.734] | 0.661 [0.651-0.672] | 0.591 [0.575-0.608] | 1.49 [1.39-1.60] | 1.63 [1.60-1.67] | 1.51 [1.44-1.59] | <0.0001 | <0.0001 | <0.0001 |
| **BSA[DB]** | 0.695 [0.655-0.734] | 0.661 [0.651-0.671] | 0.592 [0.575-0.608] | 1.50 [1.40-1.61] | 1.64 [1.60-1.67] | 1.50 [1.43-1.58] | <0.0001 | <0.0001 | <0.0001 |
| **LBM[H]** | 0.694 [0.655-0.733] | 0.663 [0.652-0.673] | 0.588 [0.572-0.605] | 1.47 [1.37-1.57] | 1.63 [1.59-1.66] | 1.49 [1.41-1.57] | <0.0001 | <0.0001 | <0.0001 |
| **LBM[B]** | 0.695 [0.656-0.734] | 0.659 [0.649-0.669] | 0.579 [0.562-0.595] | 1.47 [1.38-1.58] | 1.62 [1.58-1.66] | 1.43 [1.36-1.50] | <0.0001 | <0.0001 | <0.0001 |
| **LBM[J]** | 0.697 [0.658-0.735] | 0.660 [0.649-0.670] | 0.577 [0.560-0.593] | 1.47 [1.37-1.57] | 1.66 [1.62-1.70] | 1.15 [1.12-1.17] | <0.0001 | <0.0001 | <0.0001 |
| **BMI** | 0.687 [0.647-0.727] | 0.647 [0.637-0.658] | 0.579 [0.563-0.596] | 1.45 [1.35-1.55] | 1.60 [1.57-1.64] | 1.52 [1.43-1.62] | <0.0001 | <0.0001 | <0.0001 |
| **Height^2.7** | 0.692 [0.653-0.731] | 0.662 [0.652-0.672] | 0.594 [0.578-0.611] | 1.60 [1.47-1.74] | 1.67 [1.63-1.71] | 1.43 [1.37-1.50] | <0.0001 | <0.0001 | <0.0001 |
| **Height^2.13** | 0.690 [0.651-0.730] | 0.659 [0.648-0.669] | 0.593 [0.577-0.610] | 1.62 [1.48-1.76] | 1.67 [1.63-1.71] | 1.45 [1.38-1.52] | <0.0001 | <0.0001 | <0.0001 |
| **Height^2** | 0.690 [0.650-0.729] | 0.658 [0.647-0.668] | 0.593 [0.577-0.609] | 1.62 [1.49-1.77] | 1.66 [1.62-1.70] | 1.45 [1.38-1.52] | <0.0001 | <0.0001 | <0.0001 |
| **Height^1.5** | 0.687 [0.647-0.727] | 0.653 [0.643-0.664] | 0.591 [0.575-0.608] | 1.63 [1.50-1.79] | 1.65 [1.61-1.69] | 1.45 [1.38-1.52] | <0.0001 | <0.0001 | <0.0001 |
| **Height** | 0.683 [0.643-0.723] | 0.648 [0.638-0.658] | 0.589 [0.572-0.605] | 1.65 [1.50-1.81] | 1.64 [1.60-1.68] | 1.44 [1.37-1.51] | <0.0001 | <0.0001 | <0.0001 |
| ***Unindexed*** | *0.672 [0.630-0.713]* | *0.635 [0.625-0.645]* | 0.582 [0.565-0.598] | *1.68 [1.52-1.85]* | *1.59 [1.55-1.63]* | *1.40 [1.34-1.47]* | *<0.0001* | *<0.0001* | *<0.0001* |

**Supplementary Table 15.** Statistical analyses for 5-year cardiovascular mortality for RV diameter indexed by different metrics.

BSA = body surface area, [M] = Mosteller, [DB] = Du Bois, LBM = lean body mass, [H] = Hume, [B] = Boer, [J] = James, BMI = body mass index.

| | C statistic | | | Hazard Ratio per 1 SD increment | | | p value | | |
|---|---|---|---|---|---|---|---|---|---|
| | **BMI <18.5 kg/m$^2$** | **BMI 18.5-30 kg/m$^2$** | **BMI ≥30 kg/m$^2$** | **BMI <18.5 kg/m$^2$** | **BMI 18.5-30 kg/m$^2$** | **BMI ≥30 kg/m$^2$** | **BMI <18.5 kg/m$^2$** | **BMI 18.5-30 kg/m$^2$** | **BMI ≥30 kg/m$^2$** |
| **Weight** | 0.599 [0.481-0.717] | 0.652 [0.613-0.691] | 0.656 [0.597-0.714] | 1.04 [0.79-1.38] | 1.64 [1.49-1.82] | 2.29 [1.71-3.07] | 0.77 | <0.0001 | <0.0001 |
| **Weight^1.5** | 0.424 [0.310-0.539] | 0.644 [0.605-0.682] | 0.643 [0.584-0.703] | 0.96 [0.75-1.23] | 1.68 [1.50-1.88] | 2.70 [1.80-4.04] | 0.76 | <0.0001 | <0.0001 |
| **Height*Weight** | 0.594 [0.475-0.713] | 0.650 [0.611-0.688] | 0.652 [0.593-0.710] | 1.05 [0.79-1.40] | 1.60 [1.45-1.78] | 2.11 [1.59-2.81] | 0.75 | <0.0001 | <0.0001 |
| **BSA[M]^1.5** | 0.609 [0.488-0.731] | 0.654 [0.615-0.693] | 0.659 [0.601-0.717] | 1.13 [0.82-1.54] | 1.60 [1.45-1.76] | 2.02 [1.58-2.59] | 0.46 | <0.0001 | <0.0001 |
| **BSA[DB]^1.5** | 0.611 [0.488-0.734] | 0.653 [0.614-0.692] | 0.660 [0.602-0.718] | 1.18 [0.84-1.64] | 1.58 [1.44-1.74] | 1.92 [1.53-2.42] | 0.34 | <0.0001 | <0.0001 |
| **BSA[M]** | 0.613 [0.487-0.738] | 0.650 [0.610-0.690] | 0.666 [0.609-0.723] | 1.24 [0.88-1.76] | 1.58 [1.44-1.73] | 1.89 [1.53-2.33] | 0.23 | <0.0001 | <0.0001 |
| **BSA[DB]** | 0.617 [0.493-0.741] | 0.650 [0.610-0.690] | 0.668 [0.611-0.724] | 1.29 [0.90-1.85] | 1.57 [1.43-1.72] | 1.84 [1.50-2.25] | 0.17 | <0.0001 | <0.0001 |
| **LBM[H]** | 0.623 [0.496-0.751] | 0.644 [0.604-0.684] | 0.655 [0.597-0.712] | 1.37 [0.93-2.01] | 1.55 [1.41-1.71] | 1.76 [1.43-2.17] | 0.11 | <0.0001 | <0.0001 |
| **LBM[B]** | 0.621 [0.492-0.751] | 0.636 [0.597-0.676] | 0.636 [0.577-0.694] | 1.40 [0.94-2.08] | 1.54 [1.39-1.70] | 1.61 [1.31-1.97] | 0.10 | <0.0001 | <0.0001 |
| **LBM[J]** | 0.606 [0.482-0.730] | 0.637 [0.597-0.677] | 0.635 [0.577-0.692] | 1.13 [0.83-1.54] | 1.57 [1.41-1.74] | 1.47 [1.22-1.78] | 0.45 | <0.0001 | <0.0001 |
| **BMI** | 0.566 [0.431-0.702] | 0.632 [0.592-0.673] | 0.646 [0.586-0.705] | 1.02 [0.77-1.36] | 1.64 [1.47-1.82] | 2.15 [1.62-2.85] | 0.88 | <0.0001 | <0.0001 |
| **Height^2.7** | 0.657 [0.532-0.782] | 0.642 [0.603-0.681] | 0.662 [0.605-0.718] | 1.67 [1.06-2.63] | 1.57 [1.41-1.75] | 1.57 [1.33-1.86] | 0.028 | <0.0001 | <0.0001 |
| **Height^2.13** | 0.666 [0.542-0.791] | 0.642 [0.602-0.682] | 0.666 [0.609-0.722] | 1.66 [1.07-2.57] | 1.58 [1.42-1.75] | 1.62 [1.37-1.91] | 0.025 | <0.0001 | <0.0001 |
| **Height^2** | 0.668 [0.544-0.792] | 0.642 [0.602-0.682] | 0.667 [0.611-0.724] | 1.65 [1.07-2.56] | 1.58 [1.42-1.75] | 1.62 [1.37-1.92] | 0.024 | <0.0001 | <0.0001 |
| **Height^1.5** | 0.668 [0.544-0.792] | 0.640 [0.599-0.680] | 0.669 [0.612-0.727] | 1.63 [1.07-2.51] | 1.57 [1.42-1.74] | 1.65 [1.39-1.95] | 0.024 | <0.0001 | <0.0001 |
| **Height** | 0.665 [0.536-0.793] | 0.634 [0.593-0.675] | 0.670 [0.612-0.728] | 1.62 [1.06-2.47] | 1.56 [1.41-1.73] | 1.65 [1.40-1.96] | 0.026 | <0.0001 | <0.0001 |
| ***Unindexed*** | *0.650 [0.510-0.790]* | *0.617 [0.576-0.659]* | 0.666 [0.606-0.725] | *1.58 [1.02-2.43]* | *1.53 [1.37-1.70]* | *1.61 [1.36-1.89]* | *0.039* | *<0.0001* | *<0.0001* |
